# Simulation based composite likelihood

**DOI:** 10.1007/s11222-025-10584-z

**Published:** 2025-02-25

**Authors:** Lorenzo Rimella, Chris Jewell, Paul Fearnhead

**Affiliations:** 1 ESOMAS, University of Turin, Via Verdi 8, 10124 Turin, Italy; 2 Statistics Initiative, Collegio Carlo Alberto, Piazza Arbarello 8, 10122 Turin, Italy; 3 Mathematical Sciences, Lancaster University, Lancaster, LA14YF UK

**Keywords:** Hidden Markov model, Composite likelihood, Monte Carlo approximation, Individual-based models

## Abstract

Inference for high-dimensional hidden Markov models is challenging due to the exponential-in-dimension computational cost of calculating the likelihood. To address this issue, we introduce an innovative composite likelihood approach called “Simulation Based Composite Likelihood” (SimBa-CL). With SimBa-CL, we approximate the likelihood by the product of its marginals, which we estimate using Monte Carlo sampling. In a similar vein to approximate Bayesian computation (ABC), SimBa-CL requires multiple simulations from the model, but, in contrast to ABC, it provides a likelihood approximation that guides the optimization of the parameters. Leveraging automatic differentiation libraries, it is simple to calculate gradients and Hessians to not only speed up optimization but also to build approximate confidence sets. We present extensive empirical results which validate our theory and demonstrate its advantage over SMC, and apply SimBa-CL to real-world Aphtovirus data.

**Supplementary Information:**

The online version contains supplementary material available at 10.1007/s11222-025-10584-z.

## Introduction

Discrete-state hidden Markov models (HMMs) are common in many applications, such as epidemics (Keeling and Rohani 2011), systems biology (Wilkinson 2018) and ecology (Glennie et al. 2023). Increasingly there is interest in individual-based models (e.g. Rimella et al. 2023b), in which the HMM explicitly describes the state of each individual agent in a population. For example, an individual-based epidemic model may characterize each person in a population as having a latent state, being either susceptible, infected, or recovered. This state is typically observed noisily, with a sample of individuals being detected as infected with a possibly imperfect diagnostic test (e.g. Cocker et al. 2023). Thus, whilst there may be only a small number of states for each individual, this corresponds to a latent state-space that grows exponentially with the number of individuals.

In theory, likelihood calculations for such discrete-state HMMs are tractable using the forward-backward recursions (Scott 2002). However, the computational cost of these recursions is at least linear in the size of the state-space of the HMM: this means that they are infeasible for individual-based models with moderate or larger population sizes. This has led to a range of approximate inference methods. These include Monte Carlo methods such as Markov chain Monte Carlo (MCMC) and sequential Monte Carlo (SMC). Whilst such methods can work well, often they scale poorly with the population size—which may lead to poor mixing of MCMC algorithms or large Monte Carlo variance of the weights in SMC. An alternative approach is approximate Bayesian computation (ABC), where one simulates from the model for different parameter values, and then approximates the posterior for the parameter based on how similar each simulated dataset is to the true data. Such a method needs informative, low-dimensional, summary statistics to be available so that one can accurately measure how close a simulated dataset is to the true data. Furthermore, ABC can struggle with complex models with many parameters, as the number of summary statistics needs to increase with the number of parameters (Fearnhead and Prangle 2012).

In this paper, we consider individual-based HMMs where we have individual-level observations. We present a computationally efficient method for inference that is based on the simple observation: if we fix the state of all members of the population except one, then we can analytically calculate the conditional likelihood of that one individual using forward-backward recursions. This idea has been used before within MCMC algorithms that update the state of each individual in turn conditional on the states of the other individuals (Fintzi et al. 2017; Touloupou et al. 2020). Here we use it in a different way. By simulating multiple realizations of the states of the other individuals we can average the conditional likelihood to obtain a Monte Carlo estimate of the likelihood of the data for a given individual. We then sum the log of these estimated likelihoods over individuals to obtain a composite log-likelihood (Varin 2008) that can be maximized using, for example, stochastic gradient ascent, to estimate the parameters.

We introduce the general class of models we consider in Sect. 2. We then show how to obtain a Monte Carlo estimate of the likelihood for the observations associated with a single individual, which can be used as the basis of a composite likelihood for our model. The calculation of the likelihood for each individual involves accounting for feedback between the state of the individual in question, and the probability distribution of future states of the rest of the population. A computationally more efficient method can be obtained by ignoring this feedback—and we present theory that bounds the error of this approach, and shows that it can decay to zero as the population size tends to infinity. Then in Sect. 4 we show how we can get confidence regions around estimators based on maximizing our composite likelihood. We then demonstrate the efficiency for individual-based epidemic models both on simulated data and on data from the 2001 UK foot and mouth outbreak.

## Model

### Notation

Given the integer $t \in \mathbb {N}$, we denote the set of integers from 1 to *t* as [*t*], and we use [0 : *t*] if we want to include 0. Additionally, we use [*t*] as shorthand for indexing, for instance $x_{[t]}$ denotes the collection $x_1,\dots , x_t$. If *x* is an *N*-dimensionsal vector, then given an index $n \in [N]$, we use $x^n$ to denote the *n*th component of *x* and $x^{\setminus n}$ to denote the $\left( N-1\right)$-dimensional vector obtained by removing the *n*th component from *x*. If required, we augment the superscript notation and use $x^{(i)}$ to refer to the vector $x^{(i)}$ with components $x^{(i),n}$. For a finite and discrete set $\mathcal {S}$, we represent the cardinality of $\mathcal {S}$ as ${\textbf {card}}\left( S\right)$, and we use the shorthand $\sum _{x}$ to express the sum over all elements of $\mathcal {S}$. We use bold font to denote random variables and regular font for deterministic quantities. For the underlying probability measure, we commonly use *p* and, for the sake of clarity, we focus on its functional form, for instance, we use $p\left( x_t|x_{t-1},\theta \right)$ for the probability of $\textbf{x}_t=x_t$ given $\textbf{x}_{t-1}=x_{t-1}$ and the parameters $\theta$.

**Fig. 1:**
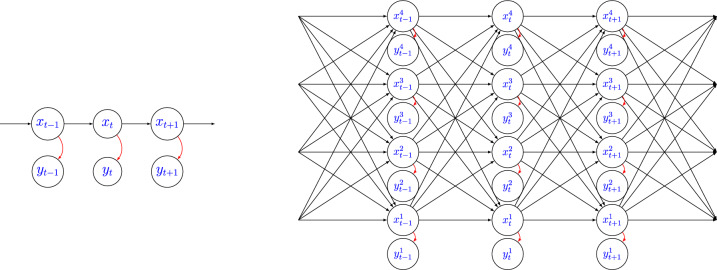
Left: conditional independence structure of a standard HMM. Right: conditional independence structure of an HMM satisfying (2), with $N=4$

### Hidden Markov models and likelihood computation

A hidden Markov model (HMM) $\left( \textbf{x}_0, \left( \textbf{x}_t, \textbf{y}_t\right) _{t \ge 1}\right)$ is a stochastic process where the unobserved process $\left( \textbf{x}_t\right) _{t \ge 0}$ is a Markov chain, and the observed process $\left( \textbf{y}_t\right) _{t \ge 1}$ is such that, for any $t\ge 1$, $\textbf{y}_t$ is conditionally independent of all the other variables given $\textbf{x}_t$. See Chopin et al. (2020) for a comprehensive review of HMMs.

Within this paper, we focus on HMMs with finite-dimensional state-spaces. Precisely, we consider $\left( \textbf{x}_t\right) _{t \ge 0}$ to take values on the state-space $\mathcal {X}^N$, which satisfies a product form, , where $\mathcal {X}$ is finite and discrete. We also consider $\left( \textbf{y}_t\right) _{t \ge 1}$ to take values in the space $\mathcal {Y}^N$, which also satisfies a product form, , but here $\mathcal {Y}$ can be of any form.

Given a collection of parameters $\theta$, an HMM is fully defined through its components: the initial distribution $p\left( x_0|\theta \right)$, which is the distribution of $\textbf{x}_0$; the transition kernel $p\left( x_t|x_{t-1},\theta \right)$, which is the distribution of $\textbf{x}_t$ given $\textbf{x}_{t-1}$; and the emission distribution $p\left( y_t|x_t,\theta \right)$, which is the distribution of $\textbf{y}_t$ given $\textbf{x}_t$. For a given time horizon $T \in \mathbb {N}$, we may assume that the data sequence $y_1,\dots ,y_T$ is generated from the aforementioned hidden Markov model with parameters $\theta ^\star$. Our primary interest is in inferring the parameter $\theta ^\star$, responsible for generating the data or, in cases where the model is not fully identifiable, a set of parameters that are equally likely. Given the assumption that $\mathcal {X}$ is finite and discrete, the probability distribution $p\left( x_0|\theta \right)$ takes the form of a probability vector with ${\textbf {card}}\left( \mathcal {X}\right) ^N$ elements, while $p\left( x_t|x_{t-1}, \theta \right)$ corresponds to a ${\textbf {card}}\left( \mathcal {X}\right) ^N \times {\textbf {card}}\left( \mathcal {X}\right) ^N$ stochastic matrix. The computation of the likelihood for HMMs with discrete state-space is relatively straightforward and involves marginalization over the entire state-space:

1 $$\begin{aligned} \begin{aligned}&p\left( y_{[T]}|\theta \right) = \sum _{x_{[0:T]}} p\left( x_0|\theta \right) \prod _{t \in [T]} p\left( x_t|x_{t-1},\theta \right) p\left( y_t|x_t, \theta \right) . \end{aligned} \end{aligned}$$

In practice, to avoid marginalizing on an exponential-in-time state-space, the likelihood is recursively computed using the forward algorithm, which recursively computes the filtering distribution $p(x_t|y_{t},\theta )$ and the likelihood increments $p(y_t|y_{[t-1]},\theta )$. The $t+1$ step of the forward algorithm comprises two operations, namely, prediction:

$$\begin{aligned} \begin{aligned}&\begin{Bmatrix} p\left( x_{t+1}|x_{t},\theta \right) \\ p\left( x_{t}|y_{[t]},\theta \right) \end{Bmatrix} \overset{\text {prediction}}{\longrightarrow } \begin{Bmatrix} p\left( x_{t+1}|y_{[t]},\theta \right) = \sum \limits _{x_{t}} p\left( x_{t+1}|x_{t},\theta \right) p\left( x_{t}|y_{[t]},\theta \right) \end{Bmatrix}, \end{aligned} \end{aligned}$$

where the transition kernel is applied to the previous filtering distribution, and correction:

$$\begin{aligned} \begin{aligned}&\begin{Bmatrix} p\left( y_{t+1}|x_{t+1},\theta \right) \\ p\left( x_{t+1}|y_{[t]},\theta \right) \end{Bmatrix} \overset{\text {correction}}{\longrightarrow }\begin{Bmatrix} p\left( x_{t+1}|y_{[t+1]},\theta \right) = \frac{p(y_{t+1}|x_{t+1},\theta ) p\left( x_{t+1}|y_{[t]},\theta \right) }{p\left( y_{t+1}|y_{[t]},\theta \right) }\\ p\left( y_{t+1}|y_{[t]},\theta \right) \hspace{-0.125cm}= \hspace{-0.25cm}\sum \limits _{x_{t+1}} p(y_{t+1}|x_{t+1},\theta ) p\left( x_{t+1}|y_{[t]},\theta \right) \end{Bmatrix} \end{aligned} \end{aligned}$$

from which the likelihood increments $p\left( y_{t}|y_{[t-1]},\theta \right)$, with $p\left( y_{1}|y_{[0]},\theta \right) {:}{=}p\left( y_{1}|\theta \right)$, are then combined to compute the likelihood:

$$\begin{aligned} p\left( y_{[T]}|\theta \right) =\prod _{t \in [T]} p\left( y_{t}|y_{[t-1]},\theta \right) . \end{aligned}$$

Despite its simplicity, the forward algorithm necessitates a marginalization on the full state-space, incurring a computational cost that is, at worst, quadratic in the cardinality of the state-space. This translates to a complexity of $\mathcal {O}\left( \textbf{card}\left( \mathcal {X}\right) ^{2N}\right)$, making the forward algorithm unfeasible for large values of *N*.

### Factorial structure

We consider HMMs with initial distribution, transition kernel, and emission distribution that satisfy the following factorizations:

2 $$\begin{aligned} \begin{aligned}&p\left( x_0|\theta \right) = \prod _{n \in [N]} p\left( x_0^n|\theta \right) ,\quad p\left( x_t|x_{t-1}, \theta \right) = \prod _{n \in [N]} p\left( x^n_t|x_{t-1},\theta \right) ,\quad p\left( y_t|x_{t}, \theta \right) = \prod _{n \in [N]} p\left( y^n_t|x_{t}^n,\theta \right) , \end{aligned} \end{aligned}$$

which essentially says that we can decompose the initial distribution in *N* probability vectors of size $\textbf{card}\left( \mathcal {X}\right)$, the transition kernel in *N* stochastic matrices that are $\textbf{card}\left( \mathcal {X}\right) \times \textbf{card}\left( \mathcal {X}\right)$, whose elements also depend on $x_{t-1}$, and the observation *n* is conditionally independent of all the other variables given $\textbf{x}_t^n$, see Fig. 1. Note that the introduced factorization does not resolve our problems as the $p\left( x^n_t|x_{t-1},\theta \right)$ still depends on the whole space at time $t-1$, not allowing a full decoupling across the dimensions. Rather, it serves as an essential foundation upon which we construct our approximation. Furthermore, the factorization given by (2) is natural in several real-world applications, including epidemics (Rimella et al. 2023a, b), traffic modelling (Silva et al. 2015) and finance (Samanidou et al. 2007).

It is important to note that (2), apart from holding for many real-world discrete-time models, does not require evenly spaced observation. Indeed, we could equivalently work on a more general version of (2), where data are observed at $0=\tau _0<\tau _1<\dots <\tau _t$, and both the transition kernel and the emission distribution are time inhomogeneous (Rimella et al. 2023b; Duffield et al. 2023):

$$\begin{aligned} \begin{aligned}&p_0\left( x_0|\theta \right) = \prod _{n \in [N]} p_0\left( x_0^n|\theta \right) ,\quad p_{\tau _t}\left( x_{\tau _t}|x_{\tau _{t-1}}, \theta \right) = \prod _{n \in [N]} p_{\tau _t}\left( x_{\tau _t}^n|x_{\tau _{t-1}},\theta \right) ,\quad p_{\tau _t}\left( y_{\tau _t}|x_{\tau _t}, \theta \right) = \prod _{n \in [N]} p_{\tau _t}\left( y^n_{\tau _t}|x_{\tau _t}^n,\theta \right) . \end{aligned} \end{aligned}$$

To avoid cumbersome notation we use the formulation from (2) throughout the paper.

## Simulation based composite likelihood: SimBa-CL

From the model structure shown in Sect. 2.3, if we fix all but one component of the latent process, $x^{\backslash n}_{[T]}$ say, we can leverage the factorization and calculate probabilities related to the time-trajectory of the remaining state, $x^n_{[T]}$, with a computational cost that is $\mathcal {O}\left( \textbf{card}\left( \mathcal {X}\right) \right)$. This idea has been used within Gibbs-style MCMC updates for epidemics see Fintzi et al. (2017); Touloupou et al. (2020). We show how to use this idea, together with using Monte Carlo to average over $x^{\backslash n}_{[T]}$, to calculate the marginal likelihoods $p (y^n_{[T]}|\theta )$. We can then use the product of these marginal likelihoods, $\prod _{n \in [N]} p (y^n_{[T]}|\theta )$, as a composite likelihood (Varin 2008) that can be maximized to estimate $\theta$.

Using $p\left( y_{[T]}|\theta \right) \approx \prod _{n \in [N]} p\left( y^n_{[T]}|\theta \right)$ still falls short, as the computation of $p\left( y_{[T]}^n|\theta \right)$ continues to require a recursive marginalization on $\mathcal {X}^N$. Yet, we can express the marginal likelihood $p\left( y_{[T]}^n|\theta \right)$ as:

3 $$\begin{aligned} \begin{aligned}&p\left( y_{[T]}^n|\theta \right) = \sum _{x_{[0:T-1]}^{\setminus n}} p\left( x_{[0:T-1]}^{\setminus n}|\theta \right) p\left( y_{[T]}^n|x_{[0:T-1]}^{\setminus n}, \theta \right) , \end{aligned} \end{aligned}$$

where:

$$\begin{aligned} \begin{aligned}&p\left( y_{[T]}^n|x_{[0:T-1]}^{\setminus n}, \theta \right) = \sum _{x_{[0:T]}^n} p\left( x_T^n|x_{T-1}, \theta \right) p\left( x_{[0:T-1]}^{n}|x_{[0:T-1]}^{\setminus n}, \theta \right) \prod _{t \in [T]} p\left( y_t^n|x_t^n, \theta \right) . \end{aligned} \end{aligned}$$

We have two necessary ingredients for calculating $p\left( y_{[T]}^n|\theta \right)$: firstly, $p\left( y_{[T]}^n|x_{[0:T-1]}^{\setminus n}, \theta \right)$, which demands *T* recursive marginalizations on $\mathcal {X}$ given $x_{[0:T-1]}^{\setminus n}$; secondly, a marginalization on $\mathcal {X}^{N-1}$ through $p\left( x_{[0:T-1]}^{\setminus n}|\theta \right)$, see (3). (3) using Monte Carlo sampling.

We refer to this procedure as “Simulation Based Composite Likelihood”, or “SimBa-CL” in short. In the following sections, we give an in-depth discussion on SimBa-CL and show how we can target the true marginals of the likelihood and build a likelihood approximation in $\mathcal {O}\left( N^2\right)$, see Sect. 3.1, how to approximate the marginals of the likelihood and build an approximation of the likelihood in $\mathcal {O}\left( N\right)$, see Sect. 3.2, and how to generalize SimBa-CL, see Sect. 3.4. For the sake of presentation, we remove the dependence on the parameter $\theta$ and focus on the filtering aspects of the algorithms for a fixed $\theta$.

### SimBa-CL with feedback

Given efficient sampling from $p \left( x_{[0:T-1]}^{\setminus n} \right)$ and low-cost evaluation of $p \left( y_{[T]}^n|x_{[0:T-1]}^{\setminus n} \right)$ for a given $x_{[0:T-1]}^{\setminus n}$, we can obtain a Monte Carlo estimate of the marginal likelihood from (3):

4 $$\begin{aligned} \begin{aligned} p\left( y^n_{[T]}\right)&\approx \frac{1}{P} \sum _{i \in [P]} p\left( y_{[T]}^n|x_{[0:T-1]}^{(i), \setminus n} \right) , \end{aligned} \end{aligned}$$

where $P \in \mathbb {N}$ is the number of Monte Carlo samples and $x_{[0:T-1]}^{(i),\setminus n} \sim p\left( x_{[0:T-1]}^{\setminus n}\right)$. Repeating (4) for all $n \in [N]$ and computing the product across *n* of these Monte Carlo estimates represents a reasonable strategy for approximating the likelihood of the model.

Two ingredients are pivotal in the computation of (4): (i) sampling from the model and (ii) calculating $p \left( y_{[T]}^n|x_{[0\!:\!T\!-\!1]}^{\setminus n} \right)$. Sampling from $p \left( x_{[0:T-1]}^{\setminus n} \right)$ can be achieved by sampling $x_{[0:T-1]}$ from $p \left( x_{[0:T-1]} \right)$ and then selecting the subset $x_{[0:T-1]}^{\setminus n}$. Sampling from the entire process is generally straightforward.

For the computation of $p \left( y_{[T]}^n|x_{[0:T-1]}^{\setminus n} \right)$, it is important to recognize that $p \left( x_{[0:T-1]}^{n}|x_{[0:T-1]}^{\setminus n} \right)$ can be reformulated as a product between the transition dynamics and the probability of observing a certain simulation outcome:

5 $$\begin{aligned} \begin{aligned}&p\left( x_{[0:T-1]}^n|x_{[0:T-1]}^{\setminus n}\right) = p\left( x_{0}^n\right) \prod _{t \in [T-1]} p\left( x_{t}^n|x_{t-1}\right) f \left( x_{t-1}^n, x_{[0:t]}^{\setminus n} \right) , \end{aligned} \end{aligned}$$

where we refer to $f(x_{t-1}^n, x_{[0:t]}^{\setminus n} ){:}{=}p ( x_{t}^{\setminus n}|x_{t-1}^n, x_{[0:t-1]}^{\setminus n} )$ as the simulation feedback, and so:

6 $$\begin{aligned} \begin{aligned}&f \left( x_{t-1}^n, x_{[0:t]}^{\setminus n} \right) = \frac{ \prod \limits _{\bar{n} \in [N] \setminus n} p\left( x_{t}^{\bar{n}}|x_{t-1}\right) }{\sum \limits _{\bar{x}_{t-1}^n} \prod \limits _{\bar{n} \in [N] \setminus n} p\left( x_{t}^{\bar{n}}|\bar{x}_{t-1}^n, x_{[t-1]}^{\setminus n}\right) p\left( \bar{x}_{t-1}^n| x_{[0:t-1]}^{\setminus n}\right) }, \end{aligned} \end{aligned}$$

where $p\left( {x}_{0}^n| x_{[0:0]}^{\setminus n}\right) = p\left( x_0^n\right)$ for the factorization of the initial distribution. The intuition is that this term accounts for how changing $x^n_{t-1}$ affects the probability of $x^{\setminus n}_t$. More details on the factorization (5) and the derivation of the simulation feedback (6) are available in Section A.1 of the supplementary material.

By reformulating $p\left( x_{[0:T-1]}^n|x_{[0:T-1]}^{\setminus n}\right)$ as depicted in (5), we arrive at the following expression:

7 $$\begin{aligned} \begin{aligned}&p\left( y_{[T]}^n|x_{[0:T-1]}^{\setminus n}\right) = \sum _{x_{[0:T]}^{n}} p\left( x_{0}^n\right) \prod _{t \in [T-1]} f \left( x_{t-1}^n, x_{[0:t]}^{\setminus n} \right) \prod_{t \in [T]} p\left( x_{t}^n|x_{t-1}\right) p\left( y_t^n|x_t^n\right) , \end{aligned} \end{aligned}$$

which resembles the likelihood of an HMM, see (1). Specifically, it comprises the usual transition dynamic term $p\left( x_t^n|x_{t-1}\right)$ accompanied by two likelihood terms: one originating from the simulation outcome $f \left( x_{t-1}^n, x_{[0:t]}^{\setminus n} \right)$, and another concerning the observation $p\left( y_t^n|x_t^n\right)$. We can then establish a forward algorithm involving two corrections, one that is correcting according to the emission distribution:

8 $$\begin{aligned} \begin{aligned}&\begin{Bmatrix} p\left( y_{t}^n | x_t^n \right) \\ p\left( x_{t}^n|y_{[t-1]}^n,x_{[0:t]}^{\setminus n}\right) \end{Bmatrix} \overset{\begin{array}{c} \text {observation}\\ \text {correction} \end{array}}{\longrightarrow }\begin{Bmatrix} p\left( x_{t}^n|y_{[t]}^n,x_{[0:t]}^{\setminus n}\right) = \frac{p\left( y_t^n | x_t^n \right) p\left( x_{t}^n|y_{[t-1]}^n,x_{[0:t]}^{\setminus n}\right) }{p\left( y_{t}^n|y_{[t-1]}^n,x_{[0:t]}^{\setminus n}\right) }\\ p\left( y_{t}^n|y_{[t-1]}^n,x_{[0:t]}^{\setminus n}\right) \qquad \qquad \qquad \qquad \qquad \\= \sum \limits _{x_t^n} p\left( y_t^n | x_t^n \right) p\left( x_{t}^n|y_{[t-1]}^n,x_{[0:t]}^{\setminus n}\right) \end{Bmatrix}, \end{aligned} \end{aligned}$$

and the other that is correcting according to the simulation feedback:

$$\begin{aligned} \begin{aligned}&\begin{Bmatrix} f\left( {x}_{t}^n, x_{[0:t+1]}^{\setminus n}\right) \\ p\left( x_{t}^n|y_{[t]}^n,x_{[0:t]}^{\setminus n}\right) \end{Bmatrix} \overset{\begin{array}{c} \text {feedback}\\ \text {correction} \end{array}}{\longrightarrow } \begin{Bmatrix} p\left( x_{t}^n|y_{[t]}^n,x_{[0:t+1]}^{\setminus n}\right) \qquad \qquad \qquad \qquad \qquad \quad \\ = \frac{f\left( {x}_{t}^n, x_{[0:t+1]}^{\setminus n}\right) p\left( x_{t}^n|y_{[t]}^n,x_{[0:t]}^{\setminus n}\right) }{p\left( x_{t+1}^{\setminus n}|y_{[t]}^n,x_{[0:t]}^{\setminus n}\right) }\\ p\left( x_{t+1}^{\setminus n}|y_{[t]}^n,x_{[0:t]}^{\setminus n}\right) \qquad \qquad \qquad \qquad \qquad \quad \\ = \sum \limits _{x_{t}^n} f\left( {x}_{t}^n, x_{[0:t+1]}^{\setminus n}\right) p\left( x_{t}^n|y_{[t]}^n,x_{[0:t]}^{\setminus n}\right) \end{Bmatrix}. \end{aligned} \end{aligned}$$

The prediction follows as is in the basic HMM scenario with $p(x_t^n|x_{t-1})$ as transition kernel and $p\left( x_{t-1}^n|y_{[t-1]}^n,x_{[0:t]}^{\setminus n}\right)$ for the distribution to update:

9 $$\begin{aligned} \begin{aligned}&\begin{Bmatrix} p(x_t^n|x_{t-1})\\ p\left( x_{t-1}^n|y_{[t-1]}^n,x_{[0:t]}^{\setminus n}\right) \end{Bmatrix} \overset{\begin{array}{c} \text {feedback}\\ \text {prediction} \end{array}}{\longrightarrow } \begin{Bmatrix} p\left( x_{t}^n|y_{[t-1]}^n,x_{[0:t]}^{\setminus n}\right) \qquad \qquad \qquad \qquad \\= \sum \limits _{x_{t-1}^n} p(x_t^n|x_{t-1}) p\left( x_{t-1}^n|y_{[t-1]}^n,x_{[0:t]}^{\setminus n}\right) \end{Bmatrix}. \end{aligned} \end{aligned}$$

The computation of the simulation feedback $f\left( x_{t-1}^n, x_{[0:t-1]}^{\setminus n}\right)$ relies on $p\left( x_{t-1}^n|x_{[0:t-1]}^{\setminus n}\right)$, the posterior distribution of ${x}_{t-1}^n$ given the simulation output as observations. Consequently, this interpretation enables the employment of another forward algorithm to compute recursively these intermediate quantities, where the correction step is given by:

10 $$\begin{aligned} \begin{aligned}&\begin{Bmatrix} \prod \limits _{\bar{n} \in [N] \setminus n} p\left( x_t^{\bar{n}}| x_{t-1} \right) \\ p\left( x_{t-1}^n|x_{[0:t-1]}^{\setminus n}\right) \end{Bmatrix} \overset{\text {correction}}{\longrightarrow } \begin{Bmatrix} p\left( x_{t-1}^n|x_{[0:t]}^{\setminus n}\right) \qquad \qquad \qquad \qquad \qquad \qquad \\= \frac{\prod \limits _{\bar{n} \in [N] \setminus n} p\left( x_t^{\bar{n}}| x_{t-1} \right) p\left( x_{t-1}^n|x_{[0:t-1]}^{\setminus n}\right) }{p(x_t^{\setminus n}|x_{[0:t-1]}^{\setminus n})}\\ p(x_t^{\setminus n}|x_{[0:t-1]}^{\setminus n})\qquad \qquad \qquad \qquad \qquad \qquad \\ = \sum \limits _{x_{t-1}^n} \prod \limits _{\bar{n} \in [N] \setminus n} p\left( x_t^{\bar{n}}| x_{t-1} \right) p\left( x_{t-1}^n|x_{[0:t-1]}^{\setminus n}\right) \end{Bmatrix}, \end{aligned} \end{aligned}$$

and the prediction follows:

11 $$\begin{aligned} \begin{aligned}&\begin{Bmatrix} p\left( x_t^n|x_{t-1}\right) \\ p\left( x_{t-1}^n|x_{[0:t]}^{\setminus n}\right) \end{Bmatrix} \overset{\text {prediction}}{\longrightarrow } \begin{Bmatrix} p\left( x_{t}^n|x_{[0:t]}^{\setminus n}\right) = \sum \limits _{x_{t-1}^n} p\left( x_t^n|x_{t-1}\right) p\left( x_{t-1}^n|x_{[0:t]}^{\setminus n}\right) \end{Bmatrix}. \end{aligned} \end{aligned}$$

An iterative application of the aforementioned steps provides a collection of likelihood increments on both the simulation output and the observations, enabling the computation of $p\left( y_{[T]}^n|x_{[0:T-1]}^{\setminus n}\right)$ as follows:

12 $$\begin{aligned} \begin{aligned}&p\left( y_{[T]}^n|x_{[0:T-1]}^{\setminus n}\right) = p\left( y_T^n|y^n_{[T-1]},x_{[0:T-1]}^{\setminus n}\right) \prod _{t \in [T-1]} p\left( y_t^n|y^n_{[t-1]},x_{[0:t]}^{\setminus n}\right) p\left( x_t^{\setminus n}|y^n_{[t-1]},x_{[0:t-1]}^{\setminus n}\right) , \end{aligned} \end{aligned}$$

where $$p\left( y_1^n|y^n_{[0]},x_{[0:0]}^{\setminus n}\right) {:}{=}p\left( y_1^n|x_{0}^{\setminus n}\right)$$ and$$p\left( x_1^{\setminus n}|y^n_{[0]},x_{[0]}^{\setminus n}\right) {:}{=}p\left( x_1^{\setminus n}|x_{[0]}^{\setminus n}, \theta \right)$$.

**Algorithm 1:**
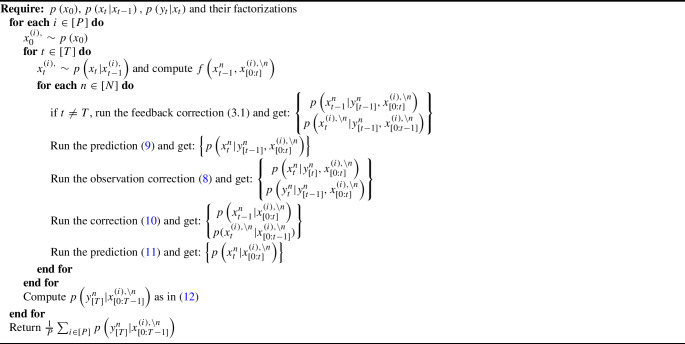
SimBa-CL with feedback

The final algorithm, named “SimBa-CL with feedback”, is presented in Algorithm 1 and it shows a computational complexity of $\mathcal {O}\left( T N^2 P \textbf{card}\left( \mathcal {X} \right) ^2\right)$, wherein *P*, *T*, *N* come from looping over number of simulations, time steps and dimensions, $\textbf{card}\left( \mathcal {X}\right) ^2$ comes from marginalizing over the state-space $\mathcal {X}$, and the extra *N* terms refers to the simulation feedback computation.

This cost can potentially be reduced to$\mathcal {O}\left( T N P \max _n \{ \textbf{card}\right. \left. \left( \textbf{Neig}\left( n\right) \right) \} \textbf{card}\left( \mathcal {X}\right) ^2\right)$ if the transition kernel $p\left( x_t^n|x_{t-1},\theta \right)$ presents some local structure. Precisely, if $p\left( x_t^n|x_{t-1},\theta \right) =p\left( x_t^n|\bar{x}_{t-1},\theta \right)$ for any $x^{\textbf{Neig}\left( n\right) }_{t-1} = \bar{x}^{\textbf{Neig}\left( n\right) }_{t-1}$, where $\textbf{Neig}$ represents a function mapping any $n \in [N]$ onto a set in the power set of [*N*]. This indicates that computing the simulation feedback can be computationally cheaper if the inter-dimension interactions are sparse. Also, the algorithm can be readily parallelized across both the dimensions and the simulations, rendering the dependence concerning (*P*, *N*) less heavy in the presence of suitable hardware.

### SimBa-CL without feedback

An important aspect of SimBa-CL with feedback is that it targets the true marginals of the likelihood. However, one could contemplate a strategy involving the removal of the simulation feedback and design a SimBa-CL that is more computationally efficient, while being “close” to SimBa-CL with feedback.

Looking back at (7), omitting the simulation feedback yields to:

13 $$\begin{aligned} \begin{aligned}&p\left( y_{[T]}^n|x_{[0:T-1]}^{\setminus n}\right) \approx \sum _{x_{[0:T]}^{n}} p\left( x_{0}^n\right) \prod _{t \in [T]} p\left( x_{t}^n|x_{t-1}\right) p\left( y_t^n|x_t^n\right){=}{:}\tilde{p}\left( y_{[T]}^n|x_{[0:T-1]}^{\setminus n}\right) , \end{aligned} \end{aligned}$$

from which we have the following marginal likelihood approximation:

$$\begin{aligned} \begin{aligned} p\left( y_{[T]}^n\right)&\approx \sum _{x_{[0:T-1]}^{\setminus n}} p\left( x_{[0:T-1]}^{\setminus n}\right) \tilde{p}\left( y_{[T]}^n|x_{[0:T-1]}^{\setminus n}\right){=}{:}\tilde{p}\left( y_{[T]}^n\right) , \end{aligned} \end{aligned}$$

where we emphasized that we are relying on an approximation by using the notation $\tilde{p}$. It can be seen that $\tilde{p}\left( y_{[T]}^n\right)$ is still a proper marginal likelihood, as it sums to one when marginalizing on $\mathcal {Y}$, but it is not the true marginal likelihood.

Upon scrutinizing (13), we can recognize the same likelihood structure as (1). This time, we are isolating our calculation to a single component *n* and fixing the others through simulation from the model. This suggests that a simple forward algorithm can be run in isolation on each dimension. Concretely, the corresponding forward algorithm will require a prediction step:

14 $$\begin{aligned} \begin{aligned}&\begin{Bmatrix} p\left( x_{t+1}^n|x_{t}\right) \\ \tilde{p}\left( x^n_{t}|y^n_{[t]}, x_{[0:t-1]}^{\setminus n}\right) \end{Bmatrix} \overset{\text {prediction}}{\longrightarrow } \begin{Bmatrix} \tilde{p}\left( x^n_{t+1}|y^n_{[t]}, x_{[0:t]}^{\setminus n}\right) \qquad \qquad \qquad \qquad \\ = \sum \limits _{x_{t}^n} p\left( x_{t+1}^n|x_{t}\right) \tilde{p}\left( x^n_{t}|y^n_{[t]}, x_{[0:t-1]}^{\setminus n}\right) \end{Bmatrix}, \end{aligned} \end{aligned}$$

and a correction step:

15 $$\begin{aligned} \begin{aligned}&\begin{Bmatrix} p\left( y_{t+1}^n|x_{t+1}^n\right) \\ \tilde{p}\left( x^n_{t+1}|y^n_{[t]}, x_{[0:t]}^{\setminus n}\right) \end{Bmatrix} \overset{\text {correction}}{\longrightarrow } \begin{Bmatrix} \tilde{p}\left( x^n_{t+1}|y^n_{[t+1]}, x_{[0:t]}^{\setminus n}\right) \qquad \qquad \qquad \qquad \\ = \frac{p\left( y_{t+1}^n|x_{t+1}^n\right) \tilde{p}\left( x^n_{t+1}|y^n_{[t]}, x_{[0:t]}^{\setminus n}\right) }{\tilde{p}\left( y^n_{t+1}|y^n_{[t]}, x_{[0:t]}^{\setminus n}\right) }\\ \tilde{p}\left( y^n_{t+1}|y^n_{[t]}, x_{[0:t]}^{\setminus n}\right) \qquad \qquad \qquad \qquad \\ = \sum \limits _{x_{t+1}^n} p\left( y_{t+1}^n|x_{t+1}^n\right) \tilde{p}\left( x^n_{t+1}|y^n_{[t]}, x_{[0:t]}^{\setminus n}\right) \end{Bmatrix}. \end{aligned} \end{aligned}$$

**Algorithm 2:**
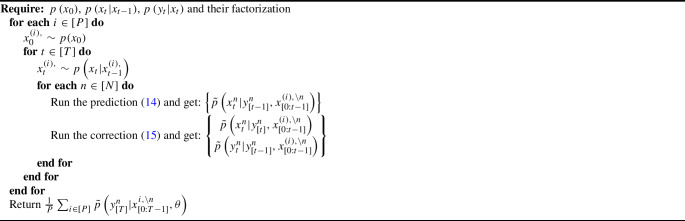
Simulation likelihood without feedback

Recursively applying (14) and (15) provides a sequence of approximate marginal likelihood increments which can be then used to approximate the marginal likelihood for a fixed simulation in the usual way:

$$\begin{aligned} \tilde{p}\left( y_{[T]}^n|x_{[0:T-1]}^{\setminus n}\right) = \prod _{t \in [T]} \tilde{p}\left( y_t^n|y^n_{[t-1]},x_{[0:t-1]}^{\setminus n}\right) , \end{aligned}$$

where $\tilde{p}\left( y_1^n|y^n_{[0]},x_{[0:0]}^{\setminus n}\right) {:}{=}\tilde{p}\left( y_1^n|x_{[0]}^{\setminus n}\right)$. The marginal likelihood approximation is then obtained as the mean of the Monte Carlo approximations, and we named this final algorithm SimBa-CL without feedback, see Algorithm 2.

When comparing Algorithm 2 with Algorithm 1, the simplicity of the latter becomes evident. The computational cost is reduced from $\mathcal {O}\left( T N^2 P \textbf{card}\left( \mathcal {X}\right) ^2 \right)$ to $\mathcal {O}\left( T N P \textbf{card}\left( \mathcal {X}\right) ^2\right)$. As with Algorithm 1, our new SimBa-CL procedure is parallelizable on both *N* and *P*.

### KL-bound for SimBa-CL with and without feedback

To evaluate the impact of excluding the simulation feedback from SimBa-CL, and so understand when is worth including the feedback, a natural approach is to compare the two estimates of the marginal likelihood:

$$\begin{aligned}&p\left( y_{[T]}^n\right) = \sum _{x_{[0:T]}^{\setminus n}} p\left( x_{[0:T]}^{\setminus n}\right) \sum _{x_{[0:T]}^n} p\left( x_{[0:T]}^{n}|x_{[0:T]}^{\setminus n}\right) \prod _{t \in [T]} p\left( y_t^n|x_t^n\right) ,\\&\tilde{p}\left( y_{[T]}^n\right) = \sum _{x_{[0:T]}^{\setminus n}} p\left( x_{[0:T]}^{\setminus n}\right) ) \sum _{x_{[0:T]}^n} p\left( x_0^{n}\right) \prod _{t \in [T]} p\left( x_t^n|x_{t-1},\right) p\left( y_t^n|x_t^n\right) . \end{aligned}$$

As both $p\left( y_{[T]}^n\right)$ and $\tilde{p}\left( y_{[T]}^n\right)$ are probability distributions on $\mathcal {Y}^{T}$, a simple way of comparing them is to measure the Kullback–Leibler divergence, which we denote with $\textbf{KL}\left[ p\left( \textbf{y}_{[T]}^n\right) || \tilde{p}\left( \textbf{y}_{[T]}^n\right) \right]$. The objective of this section is then to establish an upper bound for the $\textbf{KL}$, demonstrating that under suitable assumptions, in the large-*N* limit, the “without feedback” approximate marginal likelihood is a good approximation of the true marginal likelihood.

Naturally, we must rely on technical assumptions for theoretical results, which we will strive to explain from an intuitive perspective as much as possible. Our result relies on some assumptions, which we now explain.

#### Assumption 1

For any $n, \bar{n} \in [N]$ and for any $x_t^{\bar{n}} \in \mathcal {X}$, if $x_{t-1}, \bar{x}_{t-1} \in \mathcal {X}^N$ are such that $x_{t-1}^{\setminus {n}}= \bar{x}_{t-1}^{\setminus {n}}$ then:

$$\begin{aligned} \begin{aligned}&\left| p\left( x_t^{\bar{n}}|x_{t-1}\right) - p\left( x_t^{\bar{n}}|\bar{x}_{t-1}\right) \right| \le \frac{1}{N} \left| d_{n,\bar{n}}\left( x_{t-1}^{{n}}\right) - d_{n,\bar{n}}\left( \bar{x}_{t-1}^{{n}}\right) \right| , \end{aligned} \end{aligned}$$

where $$d_{n,\bar{n}}:\mathcal {X} \rightarrow \mathbb {R}_+$$.

Assumption 1 ensures the boundness of the transition dynamic when altering the states of only ${n} \in [N]$ at time $t-1$. This essentially asserts that changing the state of a single component at time $t-1$ minimally impacts the dynamics of the other components. In essence, the impact is measured in terms of the function $d_{n,\bar{n}}$, which shows how changes in *n* affect any other dimension $\bar{n}$. This concept is similar in flavour to the decay of correlation property explained in Rebeschini and Van Handel (2015) and Rimella and Whiteley (2022), which ensures a weak sensitivity of the conditional distributions on any *n* given a perturbation on any other dimension. Conceptually, the main emphasis is that the dynamics are only impacted by single dimension-level perturbations at the scale $N^{-1}$. In systems of increasing size with interconnected dimensions, we expect that single dimension-level changes have diminishing effects on the dimension as a whole, which is also intuitively plausible in numerous real-world applications, like individual-based models for epidemiology (Rimella et al. 2023b; Cocker et al. 2023). Observe that we could redefine $d_{n,\bar{n}}$ as $d_{n,\bar{n}} /N$ and essentially mask the $N^{-1}$ scaling. However, we made the $N^{-1}$ dependence explicit. Our theoretical results will depend on the variability of $d_{n,\bar{n}}$, and will give $O(N^{-1})$ error bounds for models where the $d_{n,\bar{n}}$ functions have variance that is bounded as *N* increases.

#### Assumption 2

For any $n, \bar{n} \in [N]$, if $x_{t-1}, \bar{x}_{t-1} \in \mathcal {X}^N$ are such that $x_{t-1}^{\setminus \bar{n}}= \bar{x}_{t-1}^{\setminus \bar{n}}$ then there exists $0< \epsilon < 1$ such that:

$$\begin{aligned} \begin{aligned}&\sum _{x^{{n}}_t} p\left( x_t^{{n}}|x_{t-1}\right) \frac{1}{p\left( x_t^{n}|\bar{x}_{t-1}\right) ^2} \le \frac{1}{\epsilon ^2}, \quad \text{and} \quad\sum _{x^{{n}}_t} p\left( x_t^{{n}}|x_{t-1}\right) \frac{1}{p\left( x_t^{n}|\bar{x}_{t-1}\right) ^3} \le \frac{1}{\epsilon ^3}. \end{aligned} \end{aligned}$$

Assumption 2 states that given two initial states $x_{t-1}, \bar{x}_{t-1}$ which differ only in their *n*th element, any transition which is possible under $x_{t-1}$ is not too unlikely under $\bar{x}_{t-1}$. Note that this assumption is not ruling out absorbing states, but rather ensures that the non-zero elements of $p\left( x_t^{{n}}|x_{t-1}\right)$ will stay not-zero in $p\left( x_t^{n}|\bar{x}_{t-1}\right)$.

Under assumptions 1 and 2 and the additional assumption that the effect of an interaction on a single dimension does not exceed *N*, we can state the following theorem.

#### Theorem 1

If $\left| d_{n,\bar{n}} \left( x^{n} \right) - d_{n,\bar{n}} \left( \bar{x}^{n} \right) \right| < N$ for any $x^n,\bar{x}^n \in \mathcal {X}$ and assumptions 1, 2 hold, then for any $n \in [N]$:

$$\begin{aligned} \begin{aligned}&\textbf{KL} \left[ p\left( \textbf{y}_{[T]}^n\right) ||\tilde{p}\left( \textbf{y}_{[T]}^n\right) \right] \le \frac{a(\epsilon )}{N} \sum _{t \in [T]} \mathbb {E} \left\{ \frac{1}{N} \sum _{\bar{n} \in [N], \bar{n} \ne n} \mathbb {V}ar \left[ d_{n,\bar{n}} \left( \textbf{x}_{t-1}^{n} \right) \Big | \textbf{x}_{[0:t-1]}^{\setminus n} \right] \right\} , \end{aligned} \end{aligned}$$

where $$a(\epsilon ) {:}{=}2\left[ \frac{1}{2 \epsilon ^2} + \frac{1}{3 \epsilon ^3} \right]$$.

#### Proof

The proof requires the Data Processing inequality, a Taylor expansion of the function $f\left( z\right) =\log \left( 1+z\right)$, and Jensen’s inequality. The full proof is available in Section A.2 of the supplementary material. $\square$

From Theorem 1 we can observe that the approximation improves when: (i) *N* increases; and (ii) the expected variance of the interaction term across dimensions decreases. Hence, our SimBa-CL without feedback will be more or less the same as the SimBa-CL with feedback whenever we are considering a sufficiently large *N* and when changing the state on one dimension does not affect the other dimensions too much.

### SimBa-CL on general partitions

Up until now, we have implicitly assumed that approximating $p\left( y_{[T]}\right)$ involves a product of marginals across all dimensions. However, it is worth considering that a complete factorization across [*N*] might not be the optimal choice. For example, when considering an epidemic model with households, it may be better to factorize over households rather than individuals, due to the strong dependencies within each household.

Consider a partition $\mathcal {K}$ on [*N*] and reformulate our likelihood approximation as follows:

$$\begin{aligned} \begin{aligned}&p\left( y_{[T]}\right) \approx \prod _{ K \in \mathcal {K}} p\left( y_{[T]}^K\right) , \quad \text {or} \quad p\left( y_{[T]}\right) \approx \prod _{ K \in \mathcal {K}} \tilde{p}_{\mathcal {K}}\left( y_{[T]}^K\right) , \end{aligned} \end{aligned}$$

where on the top we have the actual product of the true marginals and on the bottom $\tilde{p}_{\mathcal {K}}$ denotes the generalization of $\tilde{p}$. As for SimBa-CL with and without feedback we can reformulate our marginals and approximate marginals as a simulation from the model followed by an HMM likelihood:

16 $$\begin{aligned} \begin{aligned}&p\left( y_{[T]}^K\right) {:}{=}\sum _{x_{[0:T]}^{\setminus K}} p\left( x_{[0:T]}^{\setminus K}\right) \sum _{x_{[0:T]}^K} p\left( x_{[0:T]}^{K}|x_{[0:T]}^{\setminus K}\right) \prod _{t \in [T]} \prod _{n \in K} p\left( y_t^n|x_t^n\right) , \end{aligned} \end{aligned}$$

which corrects according to the interactions inside *K*, and

17 $$\begin{aligned} \begin{aligned}&\tilde{p}_\mathcal {K}\left( y_{[T]}^K\right) {:}{=}\sum _{x_{[0:T]}^{\setminus K}} p\left( x_{[0:T]}^{\setminus K}\right) \sum _{x_{[0:T]}^K} \prod _{n \in K} p\left( x_0^{n}\right) \prod _{t \in [T]} p\left( x_t^n|x_{t-1}\right) p\left( y_t^n|x_t^n\right) , \end{aligned} \end{aligned}$$

which does not use any feedback.

As seen in Sects. 3.1 and 3.2, (16) aims to approximate the true marginals over $K \in \mathcal {K}$, while (17) only offers approximations. Once again, akin to SimBa-CL with feedback, if we want to approximate the true marginals of the likelihood we need some form of simulation feedback. This time, the simulation feedback will be from $x_{[0:t]}^{\setminus K}$ onto $x_{t-1}^K$, as we are considering probability distributions on *K*.

**Table 1:** Computational cost for a single time step. We denote with $\mathcal {C}_{\mathcal {M}}(1)$ the cost of simulating from a single dimension of the model. For the SMC, $\mathcal {C}_q(1)$ is the cost of computing the proposal distribution *q* (see Rimella et al. (2023a) for an example). For SimBa-CL, we use “WF” to indicate “with feedback”, “WOF” to indicate “without feedback”, and $K_{max} {:}{=}\max _{K \in \mathcal {K}} \textbf{card}(K)$

Method	Computational cost
Simulation	$\mathcal {O}(N \mathcal {C}_{\mathcal {M}}(1))$
SMC	$\mathcal {O}(N \mathcal {C}_{\mathcal {M}}(P) + N \mathcal {C}_q(P))$
SimBa-CL WF	$\mathcal {O}(N \mathcal {C}_{\mathcal {M}}(P) + N^2 P \textbf{card}(\mathcal {X})^2)$
SimBa-CL WOF	$\mathcal {O}(N \mathcal {C}_{\mathcal {M}}(P) + N P \textbf{card}(\mathcal {X})^2)$
SimBa-CL WF on $\mathcal {K}$	$\mathcal {O}(N \mathcal {C}_{\mathcal {M}}(P) + \textbf{card}(\mathcal {K}) (N-K_{max}) P \textbf{card}(\mathcal {X})^{2 K_{max}})$
SimBa-CL WOF on $\mathcal {K}$	$\mathcal {O}(N \mathcal {C}_{\mathcal {M}}(P) + \textbf{card}(\mathcal {K}) P \textbf{card}(\mathcal {X})^{2 K_{max}})$

We can then easily adapt Algorithms 1 and 2, transitioning from a full factorization to a factorization on the partition $\mathcal {K}$. The algorithm requires operations on the space $\mathcal {X}^K$ and so a computational cost that is exponential in the maximum number of components contained in *K*. More details and theoretical results are available in Section A.3 of the supplementary material.

We refer to Table 1 for a comparison of computational costs for a single time step. The final computational cost of each algorithm is then obtained by multiplying the values in Table 1 by *T*. SMC is used as the computational baseline. We observe that SMC is more expensive to run compared to SimBa-CL whenever the time required to compute the proposal distribution exceeds the time required to run the recursion (with or without feedback) in SimBa-CL. When considering SimBa-CL on partitions, the computational cost becomes linear in the number of partition elements, but exponential in the size of the largest element of the partition (in the worst-case scenario). This is because the dimensions within each element of the partition are now considered jointly. When including the feedback in SimBa-CL on partitions, we must iterate over all the elements external to the partition, i.e. $[N]\setminus K$, requiring $N-\max _{K \in \mathcal {K}} \textbf{card}(K)$ (in the worst-case scenario).

## Inference and confidence sets

Composite likelihoods are generally obtained as the product of likelihood components, whose structure is dependent on the considered model, see Varin et al. (2011) for a review of composite likelihood methods. Both SimBa-CL with and without feedback calculate a composite likelihood as the product of the marginals of the likelihood. We can use asymptotic theory for composite likelihood to get approximate confidence regions around the maximum composite likelihood estimator.

The first step is to find the maximum composite likelihood estimator $\hat{\theta }_{CL}$ by maximizing the composite likelihood $\mathcal {L}_{CL}\left( \theta ;y_{[T]}\right) = \prod _{K \in \mathcal {K}} \mathcal {L}^K_{CL}\left( \theta ;y^K_{[T]}\right)$ or, equivalently, the composite log-likelihood $\ell _{CL}\left( \theta ;y_{[T]}\right) = \sum _{K \in \mathcal {K}}\ell ^K_{CL}\left( \theta ;y^K_{[T]}\right)$, where $\mathcal {L}^K_{CL}\left( \theta ;y^K_{[T]}\right)$ and $\ell ^K_{CL}\left( \theta ;y^K_{[T]}\right)$ depends on the considered SimBa-CL.

The second step is to notice that in the composite likelihood literature, we have some form of asymptotic normality for $\hat{\theta }_{CL}$ (Varin 2008):

$$\begin{aligned} \hat{\theta }_{CL} \overset{d}{\approx }\ \mathcal {N} \left( \theta , G\left( \theta \right) ^{-1} \right) , \end{aligned}$$

where $G\left( \theta \right)$ is the Godambe information matrix (Godambe 1960). It then follows that, upon estimating the Godambe information matrix, we can build confidence sets for $\hat{\theta }_{CL}$ as multidimensional ellipsoids.

The final step is to estimate the Godambe information matrix, which is given by $G\left( \theta \right) = S\left( \theta \right) V\left( \theta \right) ^{-1} S\left( \theta \right)$, decomposed in terms of the sensitivity matrix and the variability matrix:

$$\begin{aligned} \begin{aligned}&S\left( \theta \right) = \mathbb {E}_{\theta } \left\{ -\text {Hess}_{\theta } \left[ \ell _{CL}\left( \theta ;\textbf{y}_{[T]}\right) \right] \right\} \quad \text {and} \quad \left( \theta \right) = \mathbb {V}\text {ar}_{\theta } \left\{ \nabla _{\theta } \left[ \ell _{CL}\left( \theta ;\textbf{y}_{[T]}\right) \right] \right\} , \end{aligned} \end{aligned}$$

where $\text {Hess}_{\theta }$ and $\nabla _{\theta }$ are the Hessian and the gradient with respect to $\theta$. Given that we can compute the Hessian and the gradient given $\theta ,{y}_{[T]}$, we can also estimate expectation and the variance via simulations from the model (expected information). More details on this aspect of SimBa-CL along with approximating $S\left( \theta \right)$ and $V\left( \theta \right)$ with the actual observations (observed information) are discussed in Section B of the supplementary material, with also more experiments available in Section C.

### Bartlett identities

The computation of the Hessian can be resource-intensive and variance estimation might be noisy. We can then simplify the form of the sensitivity and variability matrix by invoking the first and second Bartlett identities. When considering SimBa-CL with feedback the identities hold asymptotically in the number of Monte Carlo samples *P*, while for SimBa-CL without feedback they hold only approximately. The sensitivity matrix and variability matrix can be then reformulated as:

$$\begin{aligned} \begin{aligned}&S\left( \theta \right) = \sum _{K \in \mathcal {K}} \mathbb {E}_{\theta } \left[ \nabla _{\theta } \ell ^K_{CL}\left( \theta ;y^K_{[T]}\right) \nabla _{\theta } \ell ^K_{CL}\left( \theta ;y^K_{[T]}\right) ^\top \right], \quad V\left( \theta \right) = \sum _{K, \tilde{K} \in \mathcal {K}} \mathbb {E}_{\theta } \left[ \nabla _{\theta } \ell ^K_{CL}\left( \theta ;y^K_{[T]}\right) \nabla _{\theta } \ell ^{\tilde{K}}_{CL}\left( \theta ;y^{\tilde{K}}_{[T]}\right) ^\top \right] , \end{aligned} \end{aligned}$$

where $=$ becomes $\approx$ if we do not include the feedback, and where both matrices can be once again estimated via simulation. More details are available in Section B.3 of the supplementary material.

## Limitations and extensions

It is evident from Table 1 that considering SimBa-CL on a partition $\mathcal {K}$ results in a computational cost that is linear in the number of elements in the partition but exponential in the size of the elements within the partition, which quickly becomes infeasible. As shown in Sects. 6.1.2 and 6.1.3, choosing a coarser partition does not affect performance when considering a model with dimensions that exhibit low correlation. This is the case, for instance, when using an individual-based model in epidemiology, where individuals have similar behaviours, e.g. they are homogeneous mixing (Ju et al. 2021; Rimella et al. 2023a). However, it is common for dimensions to cluster together in terms of correlation. For example, again in an individual-based model for epidemiology, individuals are often assigned to households and have a high likelihood of being infected by others within the same household (Rimella et al. 2023b). In such scenarios, the SimBa-CL approach described in Sect. 3.4 with $\mathcal {K}$ representing the partition induced by the households is preferable, but comes with an increased computational cost. As a solution, one could further partition households into smaller subsets and implement localized feedback to adjust accordingly. This hybrid SimBa-CL would eliminate feedback across elements of the partition while retaining feedback within the elements of the partition, which could potentially correct the approximation.

The factorial structure shown in (2) restricts SimBa-CL to discrete-time models with possibly unevenly timed observations. If we are interested in continuous-time models and the instantaneous dynamics are independent across dimensions given the current state, an Euler approximation of the continuous-time dynamics can be used to construct the discrete-time model. However, if the time between observations is large, such an Euler approximation may not be accurate. In such cases, we can use a sufficiently small discrete-time step to ensure the accuracy of the Euler approximation, with the additional time steps corresponding to points where no observations are available. This approach results in several prediction steps without correction, followed by a correction step when data are observed.

Unconditional simulation from the model has the benefit of making SimBa-CL almost as computationally efficient as simulating directly from the model. Indeed, after the simulation phase, only simple marginalizations on $\mathcal {X}$ are required, which can be even parallelized across the *N* dimensions. However, the simulation procedure does not incorporate information from the data, which makes it possible for SimBa-CL to generate realizations of $\textbf{x}_{[0:t]}$ that are inconsistent with the data and might affect its performance via the transition kernel. Fortunately, Assumption 2 guarantees that all the non-zero elements of the transition kernel remain non-zero, ruling out the possibility of SimBa-CL collapsing to zero likelihood estimates. That said, mismatches can still occur if there is significant uncertainty about the trajectory of the latent process. However, increasing the number of simulations *P* reduces the probability of such mismatches. Note that we do not imply that there are no advantages to incorporating data information during the simulation process. On the contrary, the closer the simulations are to the actual hidden trajectories, the better the performance (Whiteley and Lee 2014; Rimella et al. 2023a).

One option, for example, could be to simulate from ${p}\left( x^n_{t}|y^n_{[t]}, x_{[0:t]}^{\setminus n}, \theta \right)$ for SimBa-CL with feedback, or from $\tilde{p}\left( x^n_{t}|y^n_{[t]}, x_{[0:t-1]}^{\setminus n}, \theta \right)$ for SimBa-CL without feedback. Including the observations should make the simulations more consistent with the data, possibly reducing both the bias and the variance of SimBa-CL. From a theoretical perspective, this could even result in a better bound compared to the one from Theorem 1, which currently increases linearly with time. We provide a small experiment on this “conditional” SimBa-CL in Section C.6 of the supplementary material.

Alternatively, one could even attempt to avoid simulations from the model altogether by performing the prediction step with the modified transition kernel $\prod _{n \in [N]} p\Bigg (x^n_t|x_{t-1}^n, \mathbb {E}_{\rho ^{\setminus n}}\Bigg [\textbf{x}_{t-1}^{\setminus n} \Bigg ], \theta \Bigg )$, i.e. substituting the contribution from ${x}_{t-1}^{\setminus n}$ with its expected contribution under a distribution $\rho ^{\setminus n}$, which could be either unconditional or conditional on the data. This approach would still exploit the factorial structure (2), and the computational cost could be further reduced whenever closed-form solutions are available (Rimella et al. 2025).

It can be observed from Sects. 3.1 and 3.2 that both SimBa-CL with and without feedback require simple operations that combine the initial distribution, transition kernel, and emission distribution. If these three quantities are continuous with respect to the parameters $\theta$, then, given the Monte Carlo simulations, it is possible to compute gradients via automatic differentiation. This process is readily implemented in multiple Machine Learning libraries and can be combined with any gradient descent technique to optimize the parameters (Zeiler 2012; Kingma et al. 2014). However, the Monte Carlo simulation itself depends on the parameters $\theta$, which complicates the differentiation process. To be more precise, we consider quantities of the form $\sum _{n \in [N]} \log \left( p(y_{[T]}^n|\theta )\right)$, with $\tilde{p}$ for SimBa-CL without feedback, meaning that we need to compute:

$$\begin{aligned} \begin{aligned}&\frac{\partial p(y_{[T]}^n|\theta )}{\partial \theta } = \frac{\partial }{\partial \theta } \mathbb {E}_{\mathcal {M}} \left[ p\left( y_{[T]}^n|\textbf{x}_{[0:T-1]}^{\setminus n},\theta \right) \right] , \end{aligned} \end{aligned}$$

where $\mathcal {M}$ stands for simulating from the model. In our experiments we consider the approximation:

$$\begin{aligned} \begin{aligned} \frac{\partial p(y_{[T]}^n|\theta )}{\partial \theta }&\approx \mathbb {E}_{\mathcal {M}} \left[ \frac{\partial }{\partial \theta } p\left( y_{[T]}^n|\textbf{x}_{[0:T-1]}^{\setminus n},\theta \right) \right] . \end{aligned} \end{aligned}$$

which computes derivatives by conditioning on the results of the Monte Carlo simulation, and essentially approximates the derivative of an expectation with the expectation of a derivative. On the one hand, this approximation reduces computational complexity by ignoring part of the derivative. On the other hand, it produces biased estimates of the gradient.

As we require simulation from a finite state-space and therefore simulation from categorical random variables, we could alternatively use a continuous relaxation of categorical random variables known as the Gumbel-Softmax trick (Maddison et al. 2016; Jang et al. 2016). This technique substitutes the $\arg \max$ of i.i.d. Gumbel random variables (Gumbel 1954) with a Softmax function. In this way, sampling from a categorical distribution is expressed as a continuous function of i.i.d. random variables and suitable to the reparameterization trick (Blundell et al. 2015), which allows to exchange expectations with derivatives without any approximation. In our case we get:

$$\begin{aligned} \begin{aligned} \frac{\partial p(y_{[T]}^n|\theta )}{\partial \theta }&\approx \mathbb {E}_{\mathcal {G}} \left[ \frac{\partial }{\partial \theta }p\left( y_{[T]}^n|s(\textbf{G},\theta ,\tau ),\theta \right) \right] , \end{aligned} \end{aligned}$$

where $\textbf{G}$ is a collection of i.i.d. Gumbel random variables with distribution $\mathcal {G}$, and *s* is a composition of continuous functions involving the Gumbel random variables, the parameter $\theta$, and the temperature parameter $\tau$.

Even though this approximation produces unbiased gradient estimates as $\tau \rightarrow 0$, it is computationally heavier and challenging to use when considering large *P* and when computing confidence regions. We refer to Section C.6 of the supplementary material for a detailed discussion and additional experiments.

## Experiments

The experiments centre on the field of epidemiological modelling, and specifically they focus on individual-based models (IBMs). Individual-based models arise when we want to describe an epidemic from an individual perspective. The complexity of IBMs lies in their high-dimensional state-space, making closed-form likelihood computationally unfeasible. However, these models satisfy the factorization outlined in (2), making them the perfect candidates for our SimBa-CL.

SimBa-CL is implemented in Python using the library TensorFlow and is available at the GitHub repository: https://github.com/LorenzoRimella/SimBa-CL. All the experiments were run on a 32gb Tesla V100 GPU available on “The High End Computing” (HEC) facility at Lancaster University. For gradient computation we use TensorFlow’s default automatic differentiation, see Sect. 5 for a deeper discussion.

In Section C of the supplementary material, interested readers can find more details about the experiments reported in the paper, as well as additional experiments on the following topics: a spatial SIS individual-based model; a comparison between SimBa-CL and its extensions; the role of *P* in terms of the quality of the approximation.

**Table 2:** Comparing empirical KL between SimBa-CL and under different scenarios. All the numerical values have been multiplied by $10^9$ to improve visualization

*N*	1000	100	100	100	100	10
$\beta _0$	Base	High	Low	Base	Base	Base
$\iota$	Base	Base	Base	Base	Low	Base
$\textbf{KL}$ on feedback	0.3 (0.2)	7.5 (3.2)	0.8 (0.4)	5.9 (2.9)	1.4 (1)	49.6 (17.2)
$\textbf{KL}$ on partition	1.4 (0.3)	36.1 (6.6)	3 (0.9)	35.1 (7)	5.6 (2)	177.3 (36.5)

### The effect of feedback and partition’s choice on SimBa-CL

In this section we perform a cross-comparison on a susceptible-infected-susceptible (SIS) IBM on SimBa-CL with feedback on $\mathcal {K}=\{\{1\},\dots ,\{N\}\}$ (“fully factorized SimBa-CL with feedback”), SimBa-CL without feedback on $\mathcal {K}=\{\{1\},\dots ,\{N\}\}$ (“fully factorized SimBa-CL without feedback”), and SimBa-CL without feedback on $\mathcal {K}=\{\{1,2\},\dots ,\{N-1,N\}\}$ (“coupled SimBa-CL without feedback”), where *N* is assumed to be even.

#### Model

Building upon the framework introduced by Ju et al. (2021) and Rimella et al. (2023a), where *n* represents an individual and $w_n$ is an observed vector of covariates. We consider a $p\left( x_0^n|\theta \right)$ with an initial probability of infection of ${1}/{1+\exp {\left( -\beta _0^\top w_n\right) }}$ and a transition kernel $p\left( x_t^n|x_{t-1}, \theta \right)$ with a probability of transitioning from S to I of $1- \exp \left[ { - \lambda _n \left( {\sum _{n \in [N]} \mathbb {I}\left( x_{t-1}^n=2\right) }/{N} + \iota \right) } \right]$ and a probability of transitioning from I to S of $1-\exp \left( - \gamma _n \right)$, where $\lambda _n = {1}/{(1+\exp {\left( -\beta _{\lambda }^\top w_n\right) })}$ and $\gamma _n = {1} /{(1+\exp {\left( -\beta _{\gamma }^\top w_n\right) })}$ and with $w_n, \beta _0,\beta _\lambda ,\beta _\gamma \in \mathbb {R}^2$. Moreover we consider $p\left( y_t^n|x_{t}^n, \theta \right) = q^{x_t^n} \mathbb {I}\left( y_t^n \ne 0\right) +\left( 1- q^{x_t^n}\right) \mathbb {I}\left( y_t^n = 0\right)$, with $q \in [0,1]^2$. Unless specify otherwise, our baseline model employs $N=1000$, $T= 100$, $w_n$ to be such that $w_n^1 = 1$ and $w_n^2 \sim \textbf{Normal}\left( 0,1\right)$, and the data-generating parameters $\beta _0 = [-\log \left( \left( 1 /0.01\right) -1\right) , 0]^\top$, $\beta _{\lambda } = [-1, 2]^\top$, $\beta _{\gamma } = [-1, -1]^\top$, $q = [0.6, 0.4]^\top$ and $\iota =0.001$. It is also important to mention that the considered SIS satisfies Assumption 1 and Assumption 2, see Section A.1 of the supplementary material.

**Fig. 2:**
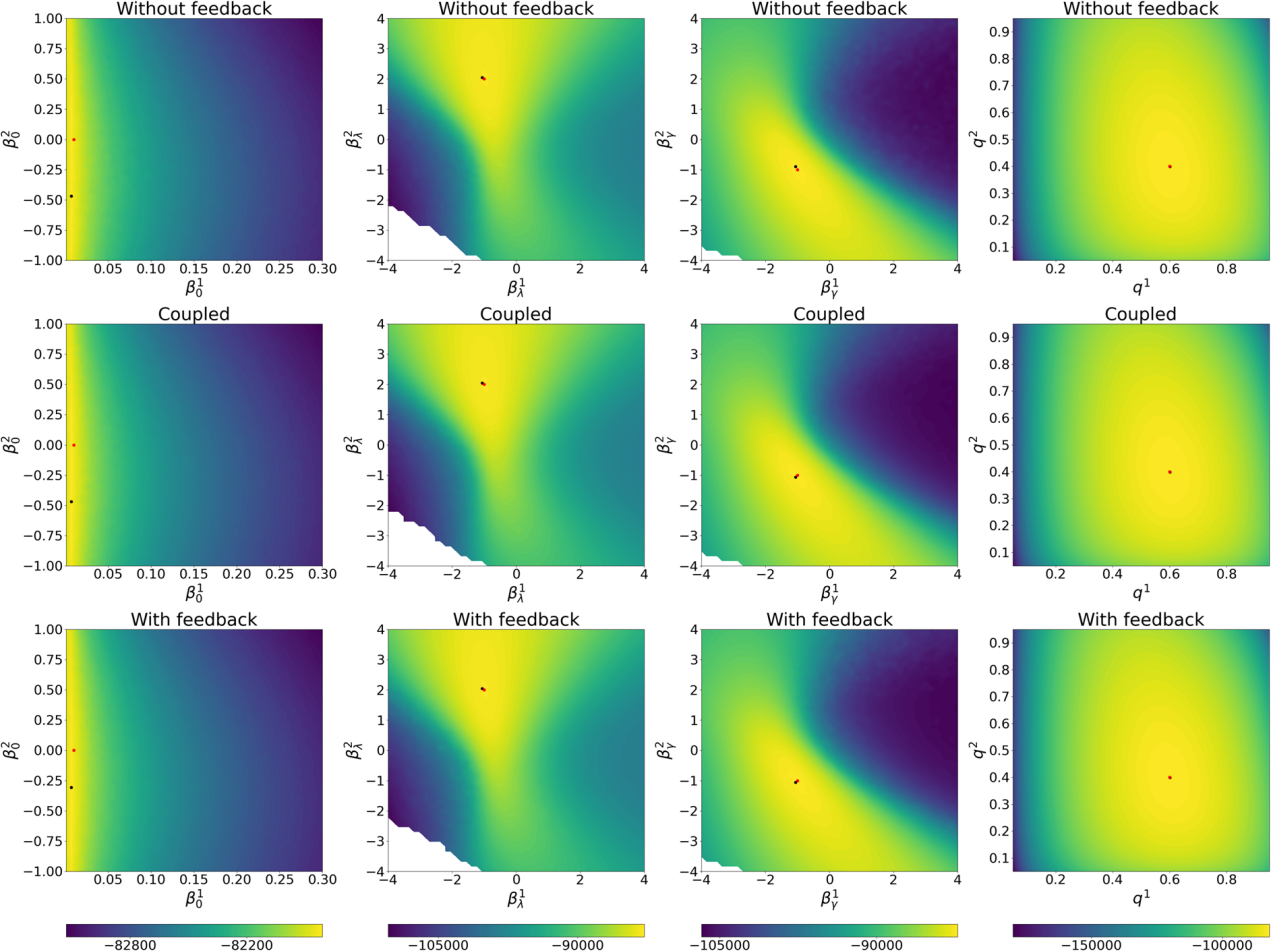
Profile log-likelihood surfaces for $\beta _0,\beta _\lambda ,\beta _\gamma ,q$. From top to bottom, fully factorized SimBa-CL without feedback, coupled SimBa-CL without feedback, and fully factorized SimBa-CL with feedback. Dots locate the data-generating parameter and the maximum on the grid

#### Empirical evaluation of the Kullback–Leibler divergence

We start by comparing our SimBa-CL methods in terms of empirical Kullback–Leibler divergence (KL) on a set of simulated data. Different settings are considered: an increasing population size $N=[10,100,1000]$; either “high” $\beta _0 = [-6.9, 0]^\top$ or “base” $\beta _0 = [-4.60, 0]^\top$ or “low” $\beta _0 = [-2.20, 0]^\top$, i.e. around either $0.1\%$ or $1\%$ or $10\%$ of initial infected; and “base” and “low” $\iota =[0.001,0.01]$. Note that different $\beta _0$ and $\iota$ control the variance of the process, as having more infected at the beginning of the epidemic or including more environmental effects results in an epidemic that is closer to the equilibrium. We set $P=1024$ for all scenarios and for both SimBa-CL with feedback, SimBa-CL without feedback and coupled SimBa-CL without feedback. All the versions of SimBa-CL were run under the data-generating parameter.

Table 2 reports the mean and standard deviations of the empirical KL per each scenario. Focusing on the fourth row, which contrasts the fully factorized SimBa-CL with and without feedback, we can notice that: increasing *N* decreases the KL, and decreasing the variance decreases the KL. Similar conclusions can be drawn for the fifth row, which compares the fully factorized SimBa-CL without feedback with the coupled SimBa-CL without feedback. These comments suggest less and less differences across the methods when increasing *N* and decreasing the variance, which is in line with our theoretical results.

#### Comparing likelihood surfaces

We proceed to undertake a comparison of profile likelihood surfaces for the baseline SIS IBM using the following protocol: (i) choose one among $\beta _0$, $\beta _{\lambda }$, $\beta _{\gamma }$ and *q*; (ii) simulate using the baseline model, and ensure at least 10 infected in the epidemic realization; (iii) create a bi-dimensional grid on the chosen parameter; (iv) per each element of the grid compute our SimBa-CL methods by fixing the other parameters to their real values. As for the previous section, we set $P=1024$ when computing all surfaces and for both SimBa-CL with feedback, SimBa-CL without feedback and coupled SimBa-CL without feedback.

The outcomes of this experiment are illustrated in Fig. 2. Interestingly, all the considered SimBa-CL exhibit a consistent shape, meaning that, in the SIS scenario, including the feedback or choosing a coarser partition has a limited impact on the overall likelihood. Furthermore, it becomes evident that all these methods effectively recover the data-generating parameter, except for $\beta _0$. Here there is an obvious identifiability issue with $\beta _0$ as $\beta _0^2=0$ implies covariate $w_n^2$ to not be used. However, given that $w_n^2$ is random, we could have more initial infection associated with $w_n^2<0$, which gives a higher likelihood to models where $\beta _0^2<0$. Similar reasoning can be replicated for $w_n^2>0$, which explains the symmetry of the likelihood surface of $\beta _0$ on the vertical axis.

**Fig. 3:**
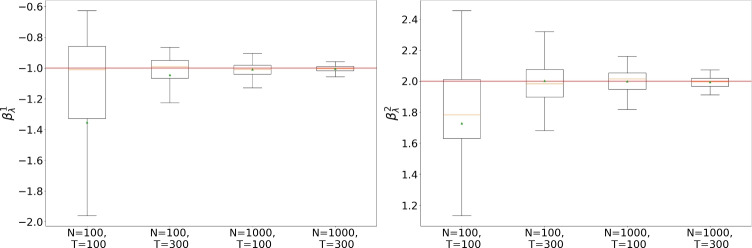
Box plots on the optimized $\beta _\lambda$. On the left, $\beta _\lambda ^1$, on the right, $\beta _\lambda ^2$. Horizontal solid lines within the boxes show the medians and triangles are used for the means. Horizontal solid lines show the true parameters

### Asymptotic properties of SimBa-CL

We can now turn to the problem of computing the maximum composite likelihood estimator and the corresponding confidence sets. Remark that for a sufficiently “regular” model and a sufficiently large *N*, including the simulation feedback and using a coarser partition bears marginal significance for SimBa-CL. We hence narrow our studies to the asymptotic properties of the fully factorized SimBa-CL without feedback. As a toy model, we consider again the SIS IBM described in Sect. 6.1.1.

Crucially, it is worth emphasizing once more that gradients and Hessians can be computed through automatic differentiation as we have implemented SimBa-CL using TensorFlow, see Sect. 5. This computational capability grants us access to the estimates of the variability and sensitivity matrix, as from Sect. 4, thereby avoiding any manual derivation of derivatives.

Remark that in both Sects. 6.2.1 and 6.2.2, we set $P=500$ for parameter optimization and $P=100$ for computing confidence regions. These values of *P* were selected to avoid out-of-memory errors.

#### Maximum Simba-CL convergence and coverage in two dimensions

We start our exploration by looking at the bi-dimensional parameter $\beta _\lambda$, given all the other parameters fixed to their baseline values. The hope is that $\beta _\lambda$ is easily identifiable as it highly influences the evolution of the epidemics, and so we can test the asymptotic properties on a well-behaved parameter.

To explore the asymptotics of SimBa-CL we investigate four scenarios with an increasing amount of data: (i) $N=100, T=100$; (ii) $N=100, T=300$; (iii) $N=1000, T=100$; (iv) $N=1000, T=300$. Per each scenario, we simulate 100 epidemics and per each dataset we optimize $\beta _\lambda$ through Adam optimization (Kingma et al. 2014), aiming to minimize the negative log-likelihood. After optimization, we have a sample of 100 bi-dimensional parameters per each scenario, which can be turned into box plots as shown in Fig. 3. As both *T* and *N* increase, we can observe an evident shrinkage towards the true parameter, suggesting consistency of the maximum SimBa-CL estimator when *N* and *T* increase.

**Fig. 4:**
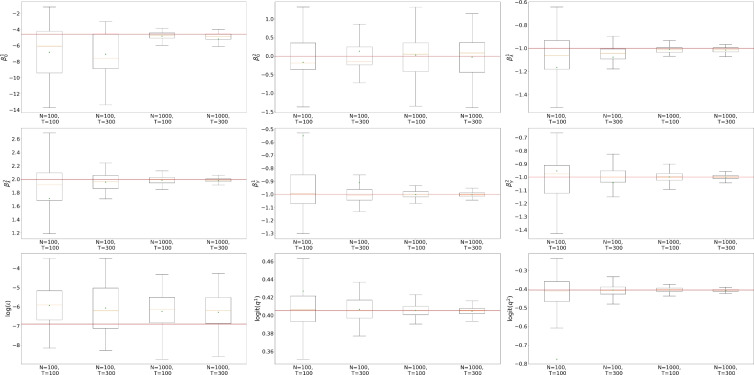
Box plots on the optimized $\theta = \left( \beta _0, \beta _\lambda , \beta _\gamma , q, \iota \right)$. Parameter labels are reported on the y-axis. Horizontal solid lines within the boxes show the medians and triangles are used for the means. Horizontal solid lines show the true parameters

**Table 3:** Empirical coverage per each parameter when computing the Godambe information matrix with and without the approximate Bartlett identities. Whenever the parameter is bi-dimensional the coverage per each component is reported in the same cell separated by “$\text {and}$”

Parameter	$\beta _0$	$\beta _\lambda$	$\beta _\gamma$	*q*	$\iota$
Without Bartlett	$0.17 \text { and }0.05$	$0.61 \text { and }0.87$	$0.8 \text { and }1.$	$0.87 \text { and } 0.5$	0.02
With Bartlett	$0.98 \text { and } 0.89$	$0.99 \text { and } 0.75$	$0.97 \text { and } 0.97$	$1. \text { and } 0.98$	0.92

Taking the investigation a step further, we analyse the empirical coverage of confidence sets built as explained in Sect. 4. We consider $N=1000, T=300$, and the optimized parameters from the previous experiment. We start by calculating the Godambe information matrix without using the Bartlett identities, and we build $95\%$ 2-dimensional confidence sets for our parameter $\beta _\lambda$. The procedure results in a coverage of 1 and so an overestimation of uncertainty. However, when repeating the same procedure using the Bartlett identities, the coverage now aligns with the theoretical coverage of 0.95. This favourable outcome can be attributed to less noisy estimates, as we are exploiting the factorization in the model and so computing expectations on lower dimensional spaces, see Section C.3 of the supplementary materials.

#### Maximum Simba-CL convergence and coverage in nine dimensions

Transitioning to a substantially more intricate scenario, we now estimate all the parameters of the model $\theta = \left( \beta _0, \beta _\lambda , \beta _\gamma , q, \iota \right)$. Analogous to the 2-dimensional case, we simulate from the model 100 times, and per each simulation, we optimize the parameters using Adam optimizer. The outcomes are reported in Fig. 4.

Foremost, it becomes apparent that increasing the value of *T* does not influence $\beta _0$. This arises because observations in the later periods carry scarce information on the initial condition. Moreover, recall that $w_n^2 \sim \textbf{Normal}\left( 0, 1\right)$ and $\beta _0^2=0$. This makes the parameter not identifiable, as commented in Sect. 6.1. This ill-posed model definition implies that increasing *N* will not improve the uncertainty around our estimate as $\beta _0^2<0$ and $\beta _0^2>0$ can be equally likely, while the unbiasedness is preserved due to the symmetry of the set of equally likely parameters. At the same time, the parameters $\iota$ and $\beta _\lambda$ are also hard to identify as smaller (or bigger) estimates of $\beta _\lambda$ will lead to bigger (or smaller) estimates of $\iota$. Indeed, $\beta _\lambda$ governs the infection rates from the community, while $\iota$ represents the environmental effect. It is then clear that generating an epidemic from $\beta _\lambda ,\iota$ is equivalent to generating one by decreasing $\beta _\lambda$ and increasing accordingly $\iota$. This correlation is especially vivid in Fig. 4, as an overestimation of $\iota$ (log-scale) leads to an underestimation of $\beta _\lambda$.

Clearly, in a 9-dimensional scenario, the process of recovering empirically the theoretical coverage is substantially more complicated. Jointly, we find 0 coverage of the 9-dimensional $95\%$ confidence sets, irrespective of whether the Bartlett identities are employed or not. We then compute the confidence intervals on single parameters by marginalizing the 9-dimensional Gaussian distribution. Marginally, we find that using the approximate Bartlett identities improves the coverages, see Table 3 for numerical values, with comments similar to the previous section for the quality of the estimates.

### Comparing SimBa-CL with sequential Monte Carlo

As SimBa-CL methods provide biased estimates of the likelihood, the objective of this section is to compare SimBa-CL methods with both sequential Monte Carlo (SMC) and block sequential Monte Carlo (BSMC) algorithms. While SMC provides an unbiased particle estimate of the likelihood (Chopin et al. 2020), this quantity can suffer high variance in high-dimensional scenarios. On the other hand, BSMC deals with the curse of dimensionality by providing a factorized, albeit biased, particle estimate of the likelihood (Rebeschini and Van Handel 2015).

Building upon the previous sections, we compare SMC and BSMC with fully factorized SimBa-CL without feedback. Regarding the SMC comparison, we consider two approaches: the auxiliary particle filter (APF) and the SMC with proposal distribution given by Rimella et al. (2023a). Due to the curse of dimensionality, we expect poor performances of the APF, hence we include the Block APF in our analysis. The block APF works as the Block particle filter (Rebeschini and Van Handel 2015), a BSMC algorithm, but it proposes particles according to the transition kernel informed by the current observation.

#### Model

In the subsequent sections, we work again on the SIS IBM from Sect. 6.1.1. However, we also analyse a susceptible-exposed-infected-removed (SEIR) IBM. Specifically, we still have some bi-dimensional covariates $w_n$, while $p(x_0^n|\theta )$ is now 4-dimensional with the second and the fourth components being zero and the first and the third components being as in SIS IBM. Similarly, the transition kernel $p(x_t^n|x_{t-1}, \theta )$ is a 4 by 4 matrix with the same dynamics of SIS IBM when considering transitions from S to E and from I to R, with the addition of a transition E to I with probability $1-\exp (-\rho )$. Unless specified otherwise, we consider as the baseline model the one with $N=1000$, $T= 100$ and the data-generating parameters set to $\beta _0 = [-\log \left( \left( 1 /0.01\right) -1\right) , 0]^\top$, $\beta _{\lambda } = [-1, 2]^\top$, $\rho =0.2$, $\beta _{\gamma } = [-1, -1]^\top$, $q = [0,0,0.6, 0.4]^\top$. Observe, that the first two elements of *q* are both zero, meaning that we do not observe any susceptible and exposed.

#### SimBa-CL and SMC for an individual-based SIS model

We consider the baseline SIS model with $N=1000$, and precisely: we generate the data; we run SimBa-CL and the baseline algorithms 100 times on the given data using the data-generating parameters; and we estimate the mean and standard deviation of the log-likelihood. The results are reported in Table 4.

**Table 4:** Log-likelihood means and log-likelihood standard deviations for the baseline SIS model with $N=1000$. *h* is the number of future observations included in Rimella et al. (2023a) ($h=0$ correspond to APF)

P	512	1024	2048	Time (s)
APF	$-$81103.37 (46.04)	$-$81046.57 (49.65)	$-$80976.34 (36.08)	1.05
h = 5	$-$79551.92 (1.79)	$-$79552.24 (1.6)	$-$79552.81 (1.57)	3.78
h = 10	$-$79551.9 (1.81)	$-$79552.22 (1.47)	$-$79553.01 (1.56)	5.61
Block APF	$-$79565.69 (5.84)	$-$79558.44 (3.95)	Out of memory	2.97
SimBa-CL	$-$79612.74 (3.4)	$-$79612.31 (2.37)	$-$79612.34 (1.55)	1.03

**Fig. 5:**
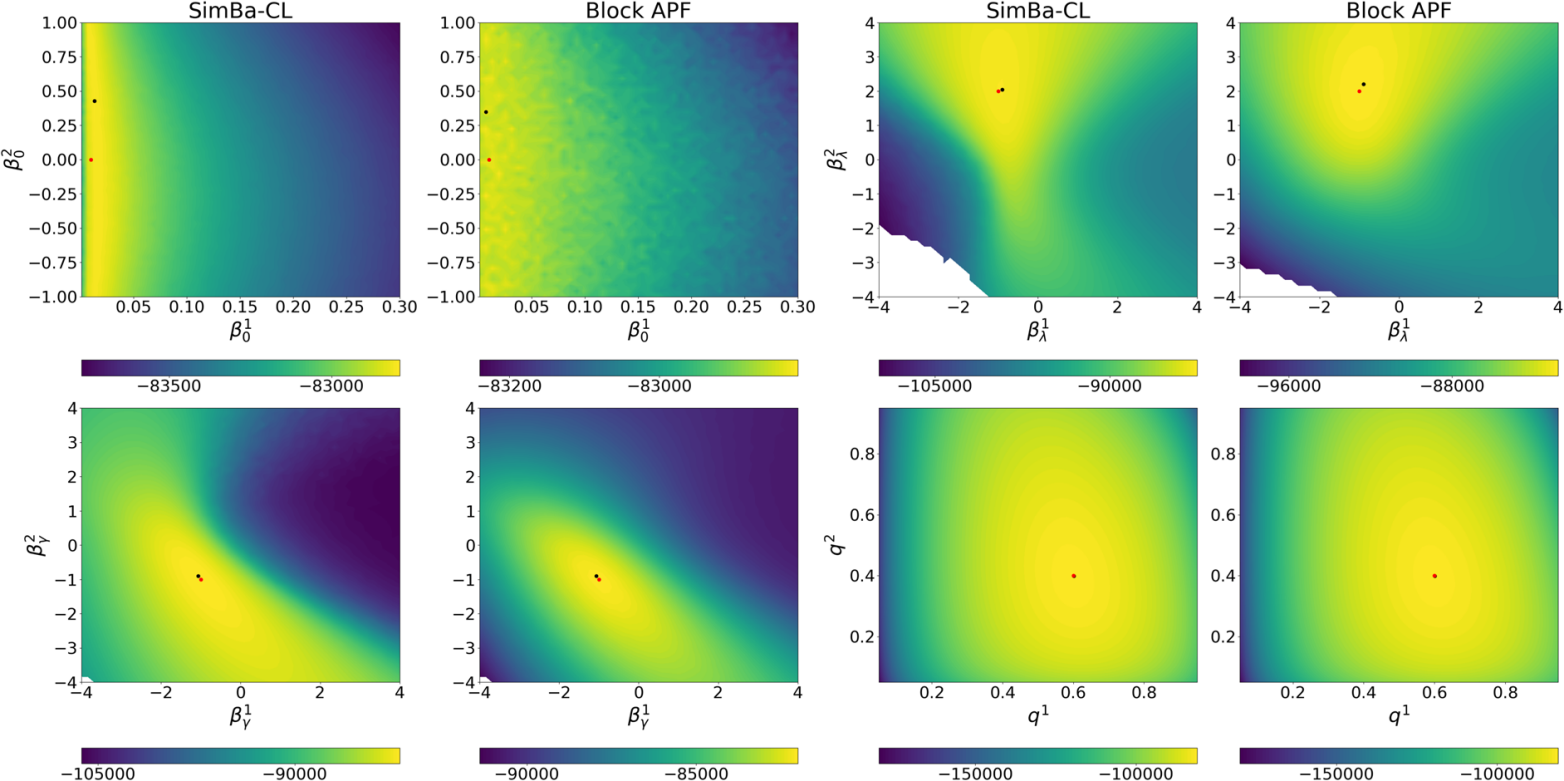
Profile log-likelihood surfaces for $\beta _0,\beta _\lambda ,\beta _\gamma ,q$ from fully factorized SimBa-CL without feedback (first and third column) and Block APF (second and the fourth columns). Dots are used for the data-generating parameter and the maximum on the grid

**Table 5:** Log-likelihood means and log-likelihood standard deviations for the baseline SEIR model with $N=1000$. *h* is the number of future observations included in Rimella et al. (2023a) ($h=0$ correspond to APF)

P	512	1024	2048	Time (s)
APF	Failed	Failed	Failed	1.2
h = 5	$-$43447.56 (52.04)	$-$43419.52 (51.08)	$-$43391.0 (52.41)	4.44
h = 20	$-$43004.55 (5.38)	$-$43001.9 (4.65)	$-$42999.76 (3.7)	11.08
h = 50	$-$42999.93 (3.44)	$-$42998.13 (2.72)	$-$42996.74 (2.39)	20.88
Block APF	Failed	Failed	Failed	2.09
SimBa	$-$43683.85 (9.54)	$-$43683.67 (7.35)	$-$43683.76 (5.16)	1.25

Notably, the method proposed by Rimella et al. (2023a) emerges as the best method in terms of log-likelihood mean and variance, as it yields unbiased estimates of the likelihood and reduces the variance. Our SimBa-CL exhibits superior computational efficiency, with a running time that is also almost three times faster than vanilla Block APF. It is worth noting that even though the bias of SimBa-CL is significant the log-likelihood variance is considerably lower than vanilla APF and comparable with the Block APF. It can be observed that the Block APF run out of memory when increasing the number of particles. This is attributed to the necessity of maintaining *P* particles for each individual and, at the same time, performing a multinomial resampling for each of these particles.

We also run a paired comparison on the profile likelihood surfaces as in Sect. 6.1, and report them in Fig. 5. It can be noticed that both SimBa-CL and Block APF generate similar likelihood surfaces whose maxima are close to the data-generating parameter. Both *P* and the number of particles were set to 1024. Unfortunately, we could not include in our studies the SMC from Rimella et al. (2023a) for computational reasons.

#### SimBa-CL and SMC for an individual-based SEIR model

The section concludes with a comparison of the baseline SEIR IBM. As for the SIS model, we simulate synthetic data, we run our SimBa-CL along with the baseline algorithms using the data-generating parameter, and we estimate the mean and variance of the resulting log-likelihood computations. The outcomes are reported in Table 5.

Note that the SEIR scenario is considerably more complex than the SIS scenario. In the SEIR case, observing only infected and removed makes it difficult for the SMC algorithms to prevent particle failure, without the use of very informative proposal distributions. The underlying intuition is that if we propose a susceptible individual at time *t* and then we observe that individual to be infected, or removed, at time $t+1$, it becomes impossible to recover from our incorrect proposal at time *t*.

Table 5 clearly shows that, to avoid failure of the SMC, we need a smart proposal distribution as the one proposed by Rimella et al. (2023a), and also a large *h* to reach a reasonable log-likelihood variance. On the other hand, our SimBa-CL is able to reach comparable log-likelihood variance almost ten times faster than the SMC.

### 2001 UK foot and mouth disease outbreak

In the year 2001, the United Kingdom experienced an outbreak of foot and mouth disease, a highly contagious virus affecting cloven-hoofed animals. Over an 8-month period, 2026 farms out of 188361 in the UK were infected, concentrated in the North and South West of England, and costing an estimated $\pounds$8 billion to public and private sectors (UK National Audit Office 2022).

**Fig. 6:**
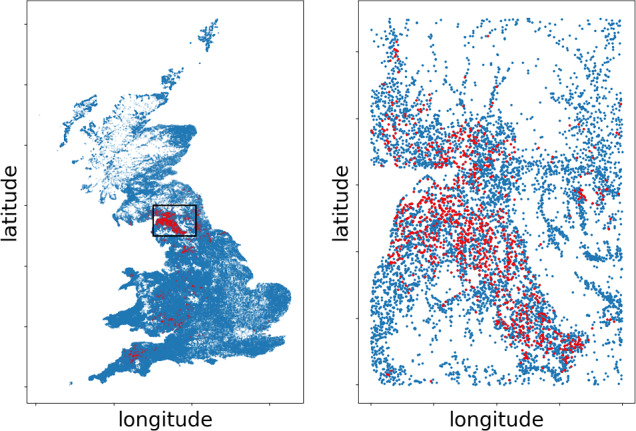
Graphical representation of the FMD outbreak. Top: the full outbreak in Great Britain. Bottom: the analysed farms. Red dots are used to indicate farms that got infected during the outbreak. The black rectangle encloses the analysed farms in the full plot

Data on the outbreak are covered by copyright, see Defra (Department for environment, food and rural affairs) website (http://www.defra.gov.uk). From the full dataset we extracted: farm coordinates, farm notification status with date, number of cattle and number of sheep in the farm. We also localised our studies in the surroundings of Cumbria and selected a total of 8791 farms, which integrates the studies by Jewell et al. (2009), see Fig. 6 for a graphical representation.

#### Model

Similar to previous models, we consider an IBM with farms as the individual. We assume farms exist in Susceptible, Infected, Notified (i.e. quarantined on detection), and Removed states (the SINR model). Transitions from S to I and I to N follow a discrete-time stochastic process, with infected farms immediately quarantined, and N to R (farm culling) occurs deterministically after 1 day.

We consider an initial probability of infection for farm *n* of $1-\exp \left\{ -\tau {\sum _{\tilde{n} \in [N]} \lambda _{\tilde{n}, n}} /{N }\right\}$, with parameter $\tau> 0$. We assume transition probabilities from I to N of $1-\exp \{-\gamma \}, \; \gamma> 0$, and from N to R of 1. We assume individual infection probabilities, i.e. transitions from S to I, of $1-\exp \left\{ - {\sum _{\tilde{n} \in [N]} \lambda _{\tilde{n}, n} \mathbb {I}\left( x_{t-1}^{\tilde{n}}=2\right) }\right\}$, where $\lambda _{\tilde{n}, n}$ is the infection pressure exerted by an infected farm $\tilde{n}$ to a susceptible farm *n* and formulated as:

18 $$\begin{aligned} \begin{aligned} \lambda _{\tilde{n},n} = \frac{\delta }{N}&\left[ \zeta \left( w_{\tilde{n}}^c\right) ^{\chi } + \left( w_{\tilde{n}}^s\right) ^{\chi } \right] \left[ \xi \left( w_n^c\right) ^{\chi } + \left( w_n^s\right) ^{\chi } \right] \frac{\psi }{E_{\tilde{n},n}^2 + \psi ^2}, \end{aligned} \end{aligned}$$

with $\delta , \xi , \zeta , \chi , \psi$ positive parameters, $w_n^c$ number of cattle in the *n*-th farm, $w_n^s$ number of sheep in the *n*-th farm and $E_{\tilde{n},n}$ the Euclidean distance in kilometres between farm $\tilde{n}$ and farm *n*. This SINR model is an example of heterogeneous mixing IBM as the infectious contacts are not homogeneous in space. The emission distribution follows the usual formulation $p\left( y_t^n|x_{t}^n, \theta \right) = q^{x_t^n} \mathbb {I}\left( y_t^n \ne 0\right) +\left( 1- q^{x_t^n}\right) \mathbb {I}\left( y_t^n = 0\right)$, with $q=[0,0,1,0]^\top$ as we observe perfectly the notified.

We set $P=500$ when performing the optimization and $P=200$ when computing confidence regions.

**Fig. 7:**
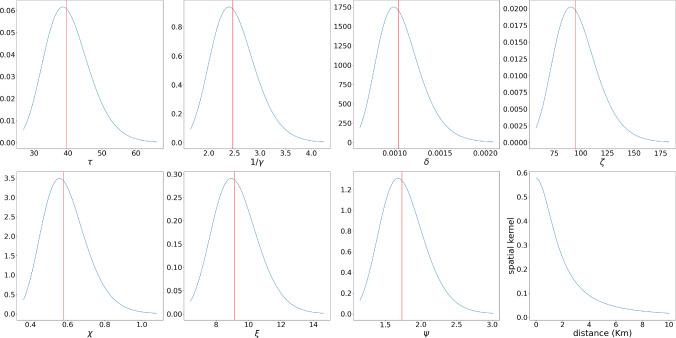
FMD parameters’ distributions and spatial kernel decay. Parameters’ labels are reported on the x-axis. Solid lines represent the maximum SimBa-CL estimator. The last plot of the second row shows the spatial decay of infectivity in Km

#### Inference

We run 100 optimization using Adam on our fully factorized SimBa-CL without feedback and select the “best” simulation according to its final SimBa-CL score. We then estimate the Godambe information matrix using the approximate Bartlett identities. As we learned the parameters in a log-scale, we need to use log-Normal when plotting the parameters distributions, see Fig. 7.

The parameter $\tau$ can be intuitively understood as the time interval between the first infection and the first notified infections, marking the onset of the notification process. In Fig. 7 we can recognize an optimal $\tau$ of about 40, suggesting a relatively slow start in notifying farms. Furthermore, we can observe a mean time before notification of about 2.5 days, leading to an estimate of 3.5 days for the mean infection period, encompassing the period from farm infection to culling. This implies a relatively fast intervention once the notification process is implemented. Also, from the last row of Fig. 7, we can notice a decrease of over $60\%$ of the infectivity after just 2 km, which could be used to define containment zones around infected farms.

Parameters $\zeta , \chi , \xi$ are more difficult to interpret as they regulate the susceptibility and infectivity of farms according to the number of animals. To help visualize their effect we produce Table 6, which shows the average susceptibility and infectivity of a medium-sized farm with only cattle, a medium-sized farm with only sheep, a large-sized farm, and a small-sized farm. From Table 6 we can deduce that the effect of owing cattle is significantly higher than the one of owing sheep for both infectivity and susceptibility, and that even small farms can affect the epidemic spread. This agrees with the study from Jewell et al. (2013) and also with the Directive of the Council of the European Union (2003).

## Discussion

In this work, we propose SimBa-CL, a novel composite likelihood approach for inference in high-dimensional HMM, which relies on simulations from the model and basic forward recursions to compute Monte Carlo approximations of the marginals of the likelihood. The computational cost of the algorithm is quadratic in the dimension *N* when considering simulations with feedback, targeting the marginals of the likelihood exactly, and can be reduced to linear when ignoring them, albeit by introducing an approximation. For well-mixing models where the effect of changing the state of one individual has a decreasing impact on the dynamics of the other individuals, we provide Kullback–Leibler divergence bounds showing that the effect of the feedback becomes negligible as *N* increases. We demonstrate that SimBa-CL is competitive with state-of-the-art SMC algorithms in terms of likelihood variance while being significantly faster and suited to automatic differentiation, allowing straightforward optimization of the parameters via Machine Learning libraries.

**Table 6:** Mean susceptibility and mean infectivity for four farms conformations

Nr. cattle	Nr. sheep	Mean susc	Mean infec
100	0	131.83	1364.99
0	1000	54.69	54.69
50	500	124.84	950.17
2	6	16.49	144.37

Although our experiments with SimBa-CL are limited to individual-based models for epidemiology, we have presented the methodology in the context of general HMMs. Indeed, SimBa-CL could be applied to standard compartmental models for epidemics (Chowell et al. 2004; Lekone and Finkenstädt 2006), where individual counts are modelled instead of individual states. Here, conjugacy properties of the model could be exploited to simplify the SimBa-CL filter and feedback (Whitehouse et al. 2023). Furthermore, state-space models and HMMs are used to model unknown skill levels in competitive sports based on game outcomes (Herbrich et al. 2006; Štrumbelj and Vračar 2012; Duffield et al. 2023). For instance, SimBa-CL on a partition of football teams could be applied to estimate the skilled players across teams over time. Also, the factorization in (2) can be recognized in traffic congestion applications (Silva et al. 2015; Rimella and Whiteley 2022), where business states are estimated using data from preallocated sensors. In this context, SimBa-CL appears to be a promising approach, in particular, SimBa-CL with feedback would allow spatial correlations to be properly accounted for, ensuring that no information is lost.

Our empirical results show good performance for parameter estimation, particularly for large *N*. We believe this stems from the fact that the approximation of ignoring feedback decreases as $N\rightarrow \infty$ and composite likelihood methods have good asymptotic properties, albeit these are shown for simpler data models (Varin 2008; Varin et al. 2011). In essence, we are conjecturing that the likelihood surface induced by SimBa-CL might have some asymptotic shape whose maximum is coalescing around the data-generating parameter or a set of equally likely parameters. However, a rigorous proof of consistency is beyond the scope of this article and we leave it to future works.

## Supplementary information

Code is open-source and available at the Github repository https://github.com/LorenzoRimella/SimBa-CL. Proofs and additional details are available in the supplementary material.

## Supplementary Information

Below is the link to the electronic supplementary material.

Supplementary file 1 (pdf 5098 KB)

## Data Availability

The data and the code to reproduce the study are open-source and available at the Github repository https://github.com/LorenzoRimella/SimBa-CLThe real data are subject to copyright.
