# Supplementary material for: Simulation based composite likelihood
Source: Stat Comput. 2025 Feb 25;35(3):58. doi: 10.1007/s11222-025-10584-z (PMC11861035; doi:10.1007/s11222-025-10584-z)
Supplement: Supplementary file 1 — (pdf 5098 KB) [file 11222_2025_10584_MOESM1_ESM.pdf]

Simulation Based Composite Likelihood: supplementary material

Lorenzo Rimella[1,2*], Chris Jewell[3] and Paul Fearnhead[3]

[1*]ESOMAS, University of Turin, Via Verdi 8, Turin, 10124, Italy.
[2]Statistics Initiative, Collegio Carlo Alberto, Piazza Arbarello 8, Turin, 10122 , Italy.
[3]Mathematical Sciences, Lancaster University, Lancaster, LA14YF, United Kingdom.

*Corresponding author(s). E-mail(s): lorenzo.rimella@unito.it;
Contributing authors: c.jewell@lancaster.ac.uk; p.fearnhead@lancaster.ac.uk;

Appendix A Simulation based composite likelihood: assumptions and proofs

In this section we provide the details about the theoretical results from the main paper. Apart from giving full proofs of the results, we will also discuss the validity of the assumptions.

A.1 Discussion on the assumptions and the simulation feedback

Here we show that our assumptions from the main paper are valid for a specific class of models. Specifically, we consider an SIS individual-based model as from the experimental section, and so:

$$p\left(x_0^n\right) = \begin{bmatrix} 1 - \frac{1}{1+\exp\left(-\beta_0^\top w_n\right)} \\ \frac{1}{1+\exp\left(-\beta_0^\top w_n\right)} \end{bmatrix};$$

$$p\left(x_t^n|x_{t-1}\right) = \begin{bmatrix} e^{-\lambda_n\left(\frac{\sum_{\bar{n}\in[N]}\mathbb{I}\left(x_{t-1}^{\bar{n}}=2\right)}{N}+\iota\right)} & 1-e^{-\lambda_n\left(\frac{\sum_{\bar{n}\in[N]}\mathbb{I}\left(x_{t-1}^{\bar{n}}=2\right)}{N}+\iota\right)} \\ 1-e^{-\gamma_n} & e^{-\gamma_n} \end{bmatrix};$$

$$p\left(y_t^n|x_t^n\right) = q^{x_t^n}\mathbb{I}\left(y_t^n \neq 0\right) + \left(1-q^{x_t^n}\right)\mathbb{I}\left(y_t^n = 0\right),$$

where λ_n, γ_n are positive and $q \in [0,1]^2$.

The first assumption we consider is reported below.

Assumption 1. *For any $n, \bar{n} \in [N]$ and for any $x_t^{\bar{n}} \in \mathcal{X}$, if $x_{t-1}, \bar{x}_{t-1} \in \mathcal{X}^N$ are such that $x_{t-1}^{\backslash n} = \bar{x}_{t-1}^{\backslash n}$ then:*

$$\left|p\left(x_t^{\bar{n}}|x_{t-1}\right) - p\left(x_t^{\bar{n}}|\bar{x}_{t-1}\right)\right| \leq \frac{1}{N}\left|d_{n,\bar{n}}\left(x_{t-1}^n\right) - d_{n,\bar{n}}\left(\bar{x}_{t-1}^n\right)\right|,$$

where $d_{n,\bar{n}} : \mathcal{X} \to \mathbb{R}_+$.

This is one of the key assumptions to prove our first result on SimBa-CL and precisely provide bounds on the Kullback-Leibler divergence between the fully factorised SimBa-CL with and without feedback.

To prove the validity of the bound we simply consider the difference between $p\left(x_t^{\bar{n}}|x_{t-1}\right)$ and $p\left(x_t^{\bar{n}}|\bar{x}_{t-1}\right)$ when $x_{t-1}, \bar{x}_{t-1} \in \mathcal{X}^N$ are such that $x_{t-1}^{\backslash n} = \bar{x}_{t-1}^{\backslash n}$.

$$\begin{aligned} & p\left(x_t^{\bar{n}}|x_{t-1}\right) - p\left(x_t^{\bar{n}}|\bar{x}_{t-1}\right) \\ &= \begin{cases} e^{-\lambda_{\bar{n}}\left(\frac{\sum_{m\in[N]}\mathbb{I}\left(x_{t-1}^{m}=2\right)}{N}+\iota\right)} - e^{-\lambda_n\left(\frac{\sum_{m\in[N]}\mathbb{I}\left(\bar{x}_{t-1}^{m}=2\right)}{N}+\iota\right)} & \text{if } x_{t-1}^{\bar{n}}=1, x_t^{\bar{n}}=1 \\ -e^{-\lambda_{\bar{n}}\left(\frac{\sum_{m\in[N]}\mathbb{I}\left(x_{t-1}^{m}=2\right)}{N}+\iota\right)} + e^{-\lambda_n\left(\frac{\sum_{m\in[N]}\mathbb{I}\left(\bar{x}_{t-1}^{m}=2\right)}{N}+\iota\right)} & \text{if } x_{t-1}^{\bar{n}}=1, x_t^{\bar{n}}=2 \\ 0 & \text{if } x_{t-1}^{\bar{n}}=2, x_t^{\bar{n}}=1 \\ 0 & \text{if } x_{t-1}^{\bar{n}}=2, x_t^{\bar{n}}=2 \end{cases} \end{aligned}$$

hence:

$$\left|p\left(x_t^{\bar{n}}|x_{t-1}\right) - p\left(x_t^{\bar{n}}|\bar{x}_{t-1}\right)\right| \leq \left|e^{-\lambda_{\bar{n}}\left(\frac{\sum_{m\in[N]}\mathbb{I}\left(x_{t-1}^{m}=2\right)}{N}+\iota\right)} - e^{-\lambda_n\left(\frac{\sum_{m\in[N]}\mathbb{I}\left(\bar{x}_{t-1}^{m}=2\right)}{N}+\iota\right)}\right|.$$

Consider the function $\exp(-x)$, from the mean value theorem we have that:

$$e^{-a} - e^{-b} = (-e^{-c})(a-b),$$

with $c \in [a, b]$ provide $a < b$. Then we can apply the mean value theorem on the domain $[0, +\infty]$ and get:

$$\left|p\left(x_t^{\bar{n}}|x_{t-1}\right) - p\left(x_t^{\bar{n}}|\bar{x}_{t-1}\right)\right| \leq \left|e^{-\lambda_{\bar{n}}\left(\frac{\sum_{m\in[N]}\mathbb{I}\left(x_{t-1}^m=2\right)}{N}+\iota\right)} - e^{-\lambda_n\left(\frac{\sum_{m\in[N]}\mathbb{I}\left(\bar{x}_{t-1}^m=2\right)}{N}+\iota\right)}\right|$$

$$= \left|e^{-c}\right|\left|\lambda_{\bar{n}}\left(\frac{\sum_{m\in[N]}\mathbb{I}\left(x_{t-1}^m=2\right)}{N}+\iota\right) - \lambda_n\left(\frac{\sum_{m\in[N]}\mathbb{I}\left(\bar{x}_{t-1}^m=2\right)}{N}+\iota\right)\right|$$

$$\leq \lambda_{\bar{n}}\left|\left(\frac{\sum_{m\in[N]}\mathbb{I}\left(x_{t-1}^m=2\right)}{N}+\iota\right) - \left(\frac{\sum_{m\in[N]}\mathbb{I}\left(\bar{x}_{t-1}^m=2\right)}{N}+\iota\right)\right|,$$

where the last step follows from c and $\lambda_{\bar{n}}$ being positive. Now remark that $x_{t-1}^{\setminus n} = \bar{x}_{t-1}^{\setminus n}$, hence:

$$\left|p\left(x_t^{\bar{n}}|x_{t-1}\right) - p\left(x_t^{\bar{n}}|\bar{x}_{t-1}\right)\right| \leq \lambda_{\bar{n}}\left|\left(\frac{\sum_{m\in[N]}\mathbb{I}\left(x_{t-1}^m=2\right)}{N}+\iota\right) - \left(\frac{\sum_{m\in[N]}\mathbb{I}\left(\bar{x}_{t-1}^m=2\right)}{N}+\iota\right)\right|$$

$$\leq \lambda_{\bar{n}}\left|\frac{\sum_{m\in[N]}\mathbb{I}\left(x_{t-1}^m=2\right)}{N} - \frac{\sum_{m\in[N]}\mathbb{I}\left(\bar{x}_{t-1}^m=2\right)}{N}\right|$$

$$\leq \frac{\lambda_{\bar{n}}}{N}\left|\mathbb{I}\left(x_{t-1}^n=2\right) - \mathbb{I}\left(\bar{x}_{t-1}^n=2\right)\right|,$$

and so our assumption is satisfied for $d_{n,\bar{n}}(x) = \lambda_{\bar{n}}\mathbb{I}(x=2)$. Note that the proof is straightforward to generalise for transition kernels that include infectivity and spatial kernels. Indeed, instead of $\lambda_{\bar{n}}\sum_{m\in[N]}\mathbb{I}\left(x_{t-1}^m=2\right)$ we could have $\lambda_{\bar{n}}\sum_{m\in[N]}\xi_m\psi_{\bar{n},m}\mathbb{I}\left(x_{t-1}^m=2\right)$ where ξ_m is the infectivity and $\psi_{\bar{n},m}$ is the spatial kernel telling how individuals $\bar{n}$ and m are connected. This alternative transition kernel will similarly satisfy our assumption with for $d_{n,\bar{n}}(x) = \lambda_{\bar{n}}\xi_n\psi_{n,\bar{n}}\mathbb{I}(x=2)$.

We now conclude by proving the validity of the second assumption, reported for completeness below.

Assumption 2. *For any $n, \bar{n} \in [N]$, if $x_{t-1}, \bar{x}_{t-1} \in \mathcal{X}^N$ are such that $x_{t-1}^{\setminus\bar{n}} = \bar{x}_{t-1}^{\setminus\bar{n}}$ then there exists $0 < \epsilon < 1$ such that:*

$$\sum_{x_t^n} p\left(x_t^n|x_{t-1}\right)\frac{1}{p\left(x_t^n|\bar{x}_{t-1}\right)^2} \leq \frac{1}{\epsilon^2}, \quad \text{and} \quad \sum_{x_t^n} p\left(x_t^n|x_{t-1}\right)\frac{1}{p\left(x_t^n|\bar{x}_{t-1}\right)^3} \leq \frac{1}{\epsilon^3}.$$

Again if we consider our SIS individuals-based model we can notice that 2 is simply requiring that the matrix obtained by doing the ratio element by element of the transition kernel computed in x_{t-1} with the square/cube of itself computed in $\bar{x}_{t-1}$

has the sum of the rows bounded. This is straightforward to prove by noting that:

$$p\left(x_t^n|x_{t-1}\right) = \begin{bmatrix} e^{-\lambda_n\left(\frac{\sum_{\bar{n}\in[N]}\mathbb{I}\left(x_{t-1}^{\bar{n}}=2\right)}{N}+\iota\right)} & 1-e^{-\lambda_n\left(\frac{\sum_{\bar{n}\in[N]}\mathbb{I}\left(x_{t-1}^{\bar{n}}=2\right)}{N}+\iota\right)} \\ 1-e^{-\gamma_n} & e^{-\gamma_n} \end{bmatrix}$$
$$\geq \begin{bmatrix} e^{-\lambda_n(1+\iota)} & 1-e^{-\lambda_n\iota} \\ 1-e^{-\gamma_n} & e^{-\gamma_n} \end{bmatrix},$$

hence if we choose $\epsilon = \max_{n\in[N]}\max\{\exp(-\lambda_n(1+\iota)), 1-\exp(-\lambda_n\iota), 1-\exp(-\gamma_n), \exp(-\gamma_n)\}$, the validity of the assumption follows trivially.

There is still an additional assumption that used for the proof of theorem, and precisely: $|d_{n,\bar{n}}(x^n) - d_{n,\bar{n}}(\bar{x}^n)| < N$. Again for the specific case of SIS we have $d_{n,\bar{n}}(x) = \lambda_{\bar{n}}\mathbb{I}(x=2)$ and so $\lambda_{\bar{n}}|\mathbb{I}(x=2) - \mathbb{I}(\bar{x}=2)|$ which is less or equal to 1 when $\lambda_{\bar{n}} < 1$ and so smaller than N. Remark that in practice $\lambda_{\bar{n}}$ is a logistic regression of some covariates of individual $\bar{n}$, guaranteeing $\lambda_{\bar{n}} < 1$.

Our assumptions are not limited to assumptions 1-2, and we need some adjusted versions of them when working with SimBa-CL for general partitions, which are again reported below for completeness.

Assumption 3. *For any $K \in \mathcal{K}$ and for any $x_t^{\bar{n}} \in \mathcal{X}$ with $\bar{n} \notin K$, if $x_{t-1}, \bar{x}_{t-1} \in \mathcal{X}^N$ are such that $x_{t-1}^{\setminus K} = \bar{x}_{t-1}^{\setminus K}$ then:*

$$\left|p\left(x_t^{\bar{n}}|x_{t-1}\right) - p\left(x_t^{\bar{n}}|\bar{x}_{t-1}\right)\right| \leq \frac{1}{N}\left|d_{K,\bar{n}}\left(x_{t-1}^n\right) - d_{K,\bar{n}}\left(\bar{x}_{t-1}^n\right)\right|.$$

where $d_{K,\bar{n}} : \mathcal{X}^K \to \mathbb{R}_+$.

Assumption 4. *For any $K, \bar{K} \in \mathcal{K}$, if $x_{t-1}, \bar{x}_{t-1} \in \mathcal{X}^N$ are such that $x_{t-1}^{\setminus \bar{K}} = \bar{x}_{t-1}^{\setminus \bar{K}}$ then there exists $0 < \epsilon < 1$ such that:*

$$\sum_{x_t^n} p\left(x_t^n|x_{t-1}\right)\frac{1}{p\left(x_t^n|\bar{x}_{t-1}\right)^2} \leq \frac{1}{\epsilon^2}, \quad \textit{and} \quad \sum_{x_t^n} p\left(x_t^n|x_{t-1}\right)\frac{1}{p\left(x_t^n|\bar{x}_{t-1}\right)^3} \leq \frac{1}{\epsilon^3}.$$

Again both assumptions are valid for the SIS case. Indeed, Assumption 3 can be proven to be valid for SIS by following the same steps and by observing that:

$$\left|p\left(x_t^{\bar{n}}|x_{t-1}\right) - p\left(x_t^{\bar{n}}|\bar{x}_{t-1}\right)\right| \leq \frac{\lambda_{\bar{n}}}{N}\left|\sum_{n\in K}\mathbb{I}\left(x_{t-1}^n = 2\right) - \mathbb{I}\left(\bar{x}_{t-1}^n = 2\right)\right|,$$

and so our $d_{K,\bar{n}}(x^K) = \lambda_{\bar{n}}\sum_{n\in K}\mathbb{I}(x^n = 2)$, from which we can also notice $|d_{K,\bar{n}}(x^K) - d_{K,\bar{n}}(\bar{x}^K)| < N$. At the same time, the proof of Assumption 4 does not change.

We now discuss the simulation feedback. Note that we have a recursive formula for $p(x^n_{[0:T]}|x^{\backslash n}_{[0:T]})$:

$$\begin{aligned}
p(x^n_{[0:T]}|x^{\backslash n}_{[0:T]}) &= p(x_T|x_{[0:T-1]})\frac{p(x^n_{[0:T-1]}|x^{\backslash n}_{[0:T-1]})}{p(x^{\backslash n}_{[T]}|x^{\backslash n}_{[0:T-1]})} \\
&= p(x^n_T|x_{T-1})\frac{p(x^{\backslash n}_T|x_{T-1})}{p(x^{\backslash n}_{[T]}|x^{\backslash n}_{[0:T-1]})}p(x^n_{[0:T-1]}|x^{\backslash n}_{[0:T-1]}) \\
&= p(x^n_T|x_{T-1}) \\
&\quad \frac{\prod_{\bar{n}\in[N],\bar{n}\neq n} p(x^{\bar{n}}_T|x_{T-1})}{\sum_{\bar{x}^n_{T-1}}\prod_{\bar{n}\in[N],\bar{n}\neq n} p(x^{\bar{n}}_T|\bar{x}^n_{T-1}, x^{\backslash n}_{[T-1]})p(\bar{x}^n_{t-1}|x^{\backslash n}_{[0:T-1]})} \\
&\quad p(x^n_{[0:T-1]}|x^{\backslash n}_{[0:T-1]}),
\end{aligned}$$

and so if we follow the same step down to $t = 1$ we obtain:

$$\begin{aligned}
p(x^n_{[0:T]}|x^{\backslash n}_{[0:T]}) &= \prod_{t\in[T]} p(x^n_t|x_{t-1}) \\
&\quad \prod_{t\in[T]} \frac{\prod_{\bar{n}\in[N],\bar{n}\neq n} p(x^{\bar{n}}_t|x_{t-1})}{\sum_{\bar{x}^n_{t-1}}\prod_{\bar{n}\in[N],\bar{n}\neq n} p(x^{\bar{n}}_t|\bar{x}^n_{t-1}, x^{\backslash n}_{[t-1]})p(\bar{x}^n_{t-1}|x^{\backslash n}_{[0:T-1]})} \\
&\quad p(x^n_0|x^{\backslash n}_0) \\
&= p(x^n_0)\prod_{t\in[T]} p(x^n_t|x_{t-1}) \\
&\quad \prod_{t\in[T]} \frac{\prod_{\bar{n}\in[N],\bar{n}\neq n} p(x^{\bar{n}}_t|x_{t-1})}{\sum_{\bar{x}^n_{t-1}}\prod_{\bar{n}\in[N],\bar{n}\neq n} p(x^{\bar{n}}_t|\bar{x}^n_{t-1}, x^{\backslash n}_{[t-1]})p(\bar{x}^n_{t-1}|x^{\backslash n}_{[0:T-1]})}.
\end{aligned} \tag{A1}$$

Here we recognise our simulation feedback indeed in the main paper we have:

$$f\left(x^n_{t-1}, x^{\backslash n}_{[0:t]}\right) = \frac{\prod_{\bar{n}\in[N]\backslash n} p\left(x^{\bar{n}}_t|x_{t-1},\right)}{\sum_{\bar{x}^n_{t-1}}\prod_{\bar{n}\in[N]\backslash n} p\left(x^{\bar{n}}_t|\bar{x}^n_{t-1}, x^{\backslash n}_{[t-1]},\right)p\left(\bar{x}^n_{t-1}|x^{\backslash n}_{[0:t-1]},\right)}.$$

We can then notice that removing the simulation feedback is like assuming some form of independence on the future as it is like we are recursively removing terms of the form $p(x^{\backslash n}_t|x_{t-1})/p(x^{\backslash n}_{[t]}|x^{\backslash n}_{[0:t-1]})$.

A.2 KL bounds for fully factorized SimBa-CL

To prove Theorem 1 we require a series of intermediate results.

We start by showing the Data processing inequality, which allows us to bound the KL between the marginals with the KL between the joints.

Lemma 1. (Data processing inequality) *Consider two joint distributions $p(x,y)$ and $q(x,y)$ and let $p_y(y)$ and $q_y(y)$ be their marginals over y, then:*

$$\mathbf{KL}(p_y(\mathbf{y})||q_y(\mathbf{y})) \leq \mathbf{KL}(p(\mathbf{x},\mathbf{y})||q(\mathbf{x},\mathbf{y})) \tag{A2}$$

provided that q is absolutely continuous with respect to p.

Proof. It can be easily proved that if q is absolutely continuous with respect to p then q_y is absolutely continuous with respect to p_y and so also the left-hand side of the statement is well-defined.

To prove (A2) we can simply use the chain rule:

$$\begin{aligned}
\mathbf{KL}(p(\mathbf{x},\mathbf{y})||q(\mathbf{x},\mathbf{y})) &= \sum_{x,y} p(x,y)\log\left(\frac{p(x,y)}{q(x,y)}\right) = \sum_{x,y} p_{x|y}(x|y)p_y(y)\log\left(\frac{p_{x|y}(x|y)p_y(y)}{q_{x|y}(x|y)q_y(y)}\right) \\
&= \sum_{x,y} p_{x|y}(x|y)p_y(y)\left[\log\left(\frac{p_{x|y}(x|y)}{q_{x|y}(x|y)}\right) + \log\left(\frac{p_y(y)}{q_y(y)}\right)\right] \\
&= \sum_{x,y} p_{x|y}(x|y)p_y(y)\log\left(\frac{p_{x|y}(x|y)}{q_{x|y}(x|y)}\right) + \sum_{x,y} p_{x|y}(x|y)p_y(y)\log\left(\frac{p_y(y)}{q_y(y)}\right) \\
&= \mathbb{E}_{p_y(\mathbf{y})}\left[\sum_x p_{x|y}(x|\mathbf{y})\log\left(\frac{p_{x|y}(x|\mathbf{y})}{q_{x|y}(x|\mathbf{y})}\right)\right] + \sum_y p_y(y)\log\left(\frac{p_y(y)}{q_y(y)}\right) \\
&= \mathbb{E}_{p_y(\mathbf{y})}\left[\mathbf{KL}(p_{x|y}(\mathbf{x}|\mathbf{y})||q_{x|y}(\mathbf{x}|\mathbf{y}))\right] + \mathbf{KL}(p_y(\mathbf{y})||q_y(\mathbf{y})) \\
&\geq \mathbf{KL}(p_y(\mathbf{y})||q_y(\mathbf{y})),
\end{aligned}$$

where the last step follows from the positivity of the KL divergence. □

The next proposition gives us a bound for the log of a ratio, which will be used in the proof of the main theorem to bound the KL.

Proposition 2. *Consider the function $f(x,y) = \log(x/y)$ with $x, y \in (0,1]$ then:*

$$f(x,y) \leq \begin{cases} -\frac{y-x}{x} + \frac{(y-x)^2}{2x^2} & y \geq x \\ -\frac{y-x}{x} + \frac{(y-x)^2}{2x^2} + \frac{(x-y)^3}{3x^2 y} & y < x \end{cases}.$$

Proof. Note that:

$$f(x,y) = log\left(\frac{x}{y}\right) = -log\left(\frac{y}{x}\right) = -log\left(1 + \frac{y-x}{x}\right)$$

and given that $y/x > 0$ also $1 + \frac{y-x}{x} > 0$, meaning that $\frac{y-x}{x} > -1$. We can then provide bounds for $h(z) = -\log(1+z)$ with $z \in (-1, +\infty)$. To do so we analyse $z \in (-1, 0)$ and $z \in [0, +\infty)$.

Let start with the case $z \in [0, +\infty)$, notice that $\tilde{h}(z) = h(z) + z - \frac{z^2}{2}$ is such that:

- $\tilde{h}(0) = h(0) = 0$;
- $\tilde{h}'(z) = h'(z) + 1 - z = \frac{-1+1-z^2}{1+z} = \frac{-z^2}{1+z} < 0$ for all $z \in [0, +\infty)$;

from which we conclude $\tilde{h}(z) \leq 0$ for all $z \in [0, +\infty)$, given that $\tilde{h}(0) = 0$ and the function is strictly decreasing on the domain. This implies $h(z) \leq -z + \frac{z^2}{2}$ on $z \in [0, +\infty)$.

Let us now analyse the case $z \in (-1, 0)$, notice that $\tilde{h}(z)$ is also decreasing on $z \in (-1, 0)$ and given that $\tilde{h}(0) = 0$, we can only prove $\tilde{h}(0) > 0$. To find an upper bound for $h(z)$ we use its Taylor expansion, and the fact that $-z \in (0, 1)$:

$$\begin{aligned}
h(z) = \sum_{i=0}^{\infty} \frac{h^{(i)}(0)z^i}{i!} &= \sum_{i=1}^{\infty} \frac{(-1)^i z^i}{i} \leq -z + \frac{z^2}{2} + \frac{1}{3}\sum_{i=3}^{\infty}(-z)^i \\
&= -z + \frac{z^2}{2} + \frac{1}{3}\left(\frac{1}{1+z} - 1 + z - z^2\right) \\
&= -z + \frac{z^2}{2} + \frac{1}{3}\left(\frac{1 - 1 + z^2 - z^2 - z^3}{1+z}\right) \\
&= -z + \frac{z^2}{2} - \frac{1}{3}\left(\frac{z^3}{1+z}\right),
\end{aligned}$$

which prove the upper bound for $z \in (-1, 0)$.

We can put everything together and complete the proof, consider $z = \frac{y-x}{x}$ we have then:

$$\begin{aligned}
f(x,y) &= -log\left(1 + \frac{y-x}{x}\right) \\
&\leq \begin{cases} -\frac{y-x}{x} + \frac{\left(\frac{y-x}{x}\right)^2}{2} & \frac{y-x}{x} \in [0, +\infty) \\ -\frac{y-x}{x} + \frac{\left(\frac{y-x}{x}\right)^2}{2} - \frac{1}{3}\left(\frac{\left(\frac{y-x}{x}\right)^3}{1+\left(\frac{y-x}{x}\right)}\right) & \frac{y-x}{x} \in (-1, 0) \end{cases} \\
&= \begin{cases} -\frac{y-x}{x} + \frac{\left(\frac{y-x}{x}\right)^2}{2} & \frac{y-x}{x} \geq 0 \\ -\frac{y-x}{x} + \frac{\left(\frac{y-x}{x}\right)^2}{2} - \frac{1}{3}\left(\frac{(y-x)^3}{x^3\left(1+\left(\frac{y-x}{x}\right)\right)}\right) & \frac{y-x}{x} < 0 \end{cases} \\
&= \begin{cases} -\frac{y-x}{x} + \frac{(y-x)^2}{2x^2} & y \geq x \\ -\frac{y-x}{x} + \frac{(y-x)^2}{2x^2} + \frac{(x-y)^3}{3x^2 y} & y < x \end{cases}.
\end{aligned}$$

□

The following corollary applies the above proposition to bound the KL between two distributions.

Corollary 3. *Consider two probability distribution $p, \tilde{p}$ on a finite state space, with p absolutely continuous with respect to $\tilde{p}$, then:*

$$
\begin{aligned}
\mathbf{KL}(p(\mathbf{x})||\tilde{p}(\mathbf{x})) \leq & \frac{1}{2}\sum_x p(x)\frac{1}{p(x)^2}\left(\tilde{p}(x)-p(x)\right)^2 \\
& + \frac{1}{3}\sum_x p(x)\frac{1}{\tilde{p}(x)^3}\left|p(x)-\tilde{p}(x)\right|^3
\end{aligned}
$$

.

Proof. The proof follows from Proposition 2 with the convention $0\log(0) = 0$ and $0\log(0/0) = 0$. Notice that whenever $p(x) = 0$ we have $0\log(0)$ and whenever $\tilde{p}(x) = 0$ we have $0\log(0/0)$ from absolute continuity, hence following the convention we can safely remove those x's from the sum and apply our proposition (apply Proposition 2 with $x = p(x)$ and $y = \tilde{p}(x) \in (0, 1]$). The bound follows from:

$$
\begin{aligned}
\mathbf{KL}(p(\mathbf{x})||\tilde{p}(\mathbf{x})) &= \sum_x p(x)\log\left(\frac{p(x)}{\tilde{p}(x)}\right) \leq \sum_x p(x)\left[-\frac{\tilde{p}(x)-p(x)}{p(x)} + \frac{(\tilde{p}(x)-p(x))^2}{2p(x)^2}\right] \\
&+ \sum_x \mathbb{I}(\tilde{p}(x) < p(x))p(x)\frac{(p(x)-\tilde{p}(x))^3}{3p(x)^2\tilde{p}(x)} \\
&= -\sum_x p(x)\left[\frac{\tilde{p}(x)-p(x)}{p(x)}\right] + \sum_x p(x)\left[\frac{(\tilde{p}(x)-p(x))^2}{2p(x)^2}\right] \\
&+ \sum_x \mathbb{I}(\tilde{p}(x) < p(x))p(x)\frac{(p(x)-\tilde{p}(x))^3}{3p(x)^2\tilde{p}(x)} \\
&= \sum_x p(x)\left[\frac{(\tilde{p}(x)-p(x))^2}{2p(x)^2}\right] + \sum_x \mathbb{I}(\tilde{p}(x) < p(x))p(x)\frac{(p(x)-\tilde{p}(x))^3}{3p(x)^2\tilde{p}(x)} \\
&= \frac{1}{2}\sum_x p(x)\frac{1}{p(x)^2}\left(\tilde{p}(x)-p(x)\right)^2 \\
&+ \frac{1}{3}\sum_x \mathbb{I}(\tilde{p}(x) < p(x))p(x)\frac{1}{p(x)^2\tilde{p}(x)}\left(p(x)-\tilde{p}(x)\right)^3 \\
&\leq \frac{1}{2}\sum_x p(x)\frac{1}{p(x)^2}\left(\tilde{p}(x)-p(x)\right)^2 \\
&+ \frac{1}{3}\sum_x \mathbb{I}(\tilde{p}(x) < p(x))p(x)\frac{1}{\tilde{p}(x)^3}\left(p(x)-\tilde{p}(x)\right)^3 \\
&\leq \frac{1}{2}\sum_x p(x)\frac{1}{p(x)^2}\left(\tilde{p}(x)-p(x)\right)^2 \\
&+ \frac{1}{3}\sum_x p(x)\frac{1}{\tilde{p}(x)^3}\left|p(x)-\tilde{p}(x)\right|^3
\end{aligned}
$$

□

We now provide the full proof of Theorem 1. We start by proving absolute continuity and so the validity of the KL computation. We then move to the Data processing inequality which we use to switch from a KL between $p(y^n_{[T]})$ and $\tilde{p}(y^n_{[T]})$ to a KL between $p(x_{[0:T]}, y^n_{[T]})$ and $\tilde{p}(x_{[0:T]}, y^n_{[T]})$. After some reformulation of the quantities, we can apply Jensen inequality and Corollary 3 which gives us a bound in terms of transition kernel difference and ratios. The final step is then the application of the assumptions along with recognizing the definition of the variance.

proof of Theorem 1. Remark that we want to compute the KL-divergence between:

- $p(y^n_{[T]}) = \sum_{x_{[0:T]}} p(x^{\setminus n}_{[0:T]}) p(x^n_{[0:T]} | x^{\setminus n}_{[0:T]}) \prod_{t\in[T]} p(y^n_t | x^n_t)$;
- $\tilde{p}(y^n_{[T]}) = \sum_{x_{[0:T]}} p(x^{\setminus n}_{[0:T]}) p(x^n_0 | \theta) \prod_{t\in[T]} p(x^n_t | x_{t-1}) p(y^n_t | x^n_t)$;

hence the first step is to prove that $p(y^n_{[T]})$ is absolutely continuous with respect to $\tilde{p}(y^n_{[T]})$, and so for a fixed $y^n_{[T]}$ we have $\tilde{p}(y^n_{[T]}) = 0$ implies $p(y^n_{[T]}) = 0$, this is necessary to ensure that the KL divergence is well-defined. Note that we have $\tilde{p}(y^n_{[T]}) = 0$ if and only if for any $x_{[0:T]}$:

1. $p(x^{\setminus n}_{[0:T]}) = 0$ or /and,
2. $p(x^n_0|\theta) \prod_{t\in[T]} p(x^n_t | x_{t-1}) = 0$ or /and,
3. $\prod_{t\in[T]} p(y^n_t | x^n_t) = 0$.

We can observe that conditions 1. and 3. implies also $p(y^n_{[T]}) = 0$, so it is enough to prove that 2. implies $p(y^n_{[T]}) = 0$ to ensure absolute continuity. Consider $p(x^n_{[0:T]} | x^{\setminus n}_{[0:T]}, \theta)$, given that from (A1) we have:

$$p(x^n_{[0:T]} | x^{\setminus n}_{[0:T]}, \theta) = p(x^n_0) \prod_{t\in[T]} p(x^n_t | x_{t-1}, \theta) f\left(x^n_{t-1}, x^{\setminus n}_{[0:t]}\right),$$

we can conclude that 2. implies $p(x^n_{[0:T]} | x^{\setminus n}_{[0:T]}, \theta) = 0$. Note that the proof of absolute continuity relies on the joint $p(x_{[0:T]}, y^n_{[T]})$ being absolutely continuous with respect to $\tilde{p}(x_{[0:T]}, y^n_{[T]})$. Given that the KL is well-defined we can now proceed with the proof.

Start by applying Lemma 1 and get:

$$\begin{aligned}\mathbf{KL}(p(\mathbf{y}^n_{[T]}) || \tilde{p}(\mathbf{y}^n_{[T]})) &\leq \mathbf{KL}(p(\mathbf{x}_{[0:T]}, \mathbf{y}^n_{[T]}) || \tilde{p}(\mathbf{x}_{[0:T]}, \mathbf{y}^n_{[T]})) \\ &= \sum_{y^n_{[T]}} \sum_{x_{[0:T]}} p(x_{[0:T]}, y^n_{[T]}) \log \left(\frac{p(x_{[0:T]}, y^n_{[T]})}{\tilde{p}(x_{[0:T]}, y^n_{[T]})} \right).\end{aligned}$$

Use now the definition of $\tilde{p}(x_{[0:T]}, y^n_{[T]})$:

$$\mathbf{KL}(p(\mathbf{y}^n_{[T]})||\tilde{p}(\mathbf{y}^n_{[T]})) \leq \sum_{y^n_{[T]}} \sum_{x_{[0:T]}} p(x_{[0:T]}, y^n_{[T]}) \log \left(\frac{p(x_{[0:T]}, y^n_{[T]})}{\tilde{p}(x_{[0:T]}, y^n_{[T]})} \right)$$

$$= \sum_{y^n_{[T]}} \sum_{x_{[0:T]}} p(x^{\setminus n}_{[0:T]}) p(x^n_{[0:T]} | x^{\setminus n}_{[0:T]}) \prod_{t \in [T]} p(y^n_t | x^n_t)$$

$$\log \left(\frac{p(x^{\setminus n}_{[0:T]}) p(x^n_{[0:T]} | x^{\setminus n}_{[0:T]}) \prod_{t \in [T]} p(y^n_t | x^n_t)}{p(x^{\setminus n}_{[0:T]}) p(x^n_0) \prod_{t \in [T]} p(x^n_t | x_{t-1}) p(y^n_t | x^n_t)} \right)$$

$$= \sum_{x_{[0:T]}} p(x^{\setminus n}_{[0:T]}) p(x^n_{[0:T]} | x^{\setminus n}_{[0:T]}) \sum_{y^n_{[T]}} \prod_{t \in [T]} p(y^n_t | x^n_t) \log \left(\frac{p(x^{\setminus n}_{[0:T]}) p(x^n_{[0:T]} | x^{\setminus n}_{[0:T]})}{p(x^{\setminus n}_{[0:T]}) p(x^n_0) \prod_{t \in [T]} p(x^n_t | x_{t-1})} \right)$$

from which we can also simplify $p(x^{\setminus n}_{[0:T]}$ and given that $\sum_{y^n_{[T]}} \prod_{t \in [T]} p(y^n_t | x^n_t) = 1$ we conclude:

$$\mathbf{KL}(p(\mathbf{y}^n_{[T]})||\tilde{p}(\mathbf{y}^n_{[T]}))$$

$$\leq \sum_{x_{[0:T]}} p(x^{\setminus n}_{[0:T]}) p(x^n_{[0:T]} | x^{\setminus n}_{[0:T]}) \sum_{y^n_{[T]}} \prod_{t \in [T]} p(y^n_t | x^n_t) \log \left(\frac{p(x^{\setminus n}_{[0:T]}) p(x^n_{[0:T]} | x^{\setminus n}_{[0:T]})}{p(x^{\setminus n}_{[0:T]}) p(x^n_0) \prod_{t \in [T]} p(x^n_t | x_{t-1})} \right)$$

$$= \sum_{x_{[0:T]}} p(x^{\setminus n}_{[0:T]}) p(x^n_{[0:T]} | x^{\setminus n}_{[0:T]}) \log \left(\frac{p(x^n_{[0:T]} | x^{\setminus n}_{[0:T]})}{p(x^n_0) \prod_{t \in [T]} p(x^n_t | x_{t-1})} \right).$$

Now we can use the recursive definition of $p(x^n_{[0:T]}|x^{\backslash n}_{[0:T]})$ in (A1):

$$\mathbf{KL}(p(\mathbf{y}^n_{[T]})||\tilde{p}(\mathbf{y}^n_{[T]})) \leq \sum_{x_{[0:T]}} p(x^{\backslash n}_{[0:T]})p(x^n_{[0:T]}|x^{\backslash n}_{[0:T]}) \log\left(\frac{p(x^n_{[0:T]}|x^{\backslash n}_{[0:T]})}{p(x^n_0)\prod_{t\in[T]} p(x^n_t|x_{t-1})}\right)$$
$$= \sum_{x_{[0:T]}} p(x_{[0:T]}) \log\left(\frac{p(x^n_{[0:T]}|x^{\backslash n}_{[0:T]})}{p(x^n_0)\prod_{t\in[T]} p(x^n_t|x_{t-1})}\right)$$
$$= \sum_{x_{[0:T]}} p(x_{[0:T]}) \log\left(\frac{p(x^n_0)\prod_{t\in[T]} p(x^n_t|x_{t-1}) f\left(x^n_{t-1}, x^{\backslash n}_{[0:t]}\right)}{p(x^n_0)\prod_{t\in[T]} p(x^n_t|x_{t-1})}\right)$$
$$= \sum_{x_{[0:T]}} p(x_{[0:T]}) \log\left(\prod_{t\in[T]} f\left(x^n_{t-1}, x^{\backslash n}_{[0:t]}\right)\right) = \sum_{x_{[0:T]}} p(x_{[0:T]}) \sum_{t\in[T]} \log\left(f\left(x^n_{t-1}, x^{\backslash n}_{[0:t]}\right)\right).$$

Note now that we can move the sum over time steps in front and, given that the argument of the logarithm depends only on $x_{[0:t-1]}, x^{\backslash n}_t$ we can also simplify $p(x_{[0:T]})$:

$$\begin{aligned}\mathbf{KL}(p(\mathbf{y}^n_{[T]})||\tilde{p}(\mathbf{y}^n_{[T]})) &\leq \sum_{x_{[0:T]}} p(x_{[0:T]}) \sum_{t\in[T]} \log\left(f\left(x^n_{t-1}, x^{\backslash n}_{[0:t]}\right)\right) \\ &= \sum_{t\in[T]} \sum_{x_{[0:t-1]}, x^{\backslash n}_t} p(x_{[0:t-1]}) \prod_{\bar{n}\in[N],\bar{n}\neq n} p(x^{\bar{n}}_t|x_{t-1}) \\ &\quad \log\left(\frac{\prod_{\bar{n}\in[N],\bar{n}\neq n} p(x^{\bar{n}}_t|x_{t-1})}{\sum_{\bar{x}^n_{t-1}} \prod_{\bar{n}\in[N],\bar{n}\neq n} p(x^{\bar{n}}_t|\bar{x}_{t-1}) p(\bar{x}^n_{t-1}|x^{\backslash n}_{[0:t-1]})}\right),\end{aligned} \tag{A3}$$

where $\bar{x}_{t-1}$ is such that $\bar{x}^{\backslash n}_{t-1} = x^{\backslash n}_{t-1}$ and $\bar{x}^n_{t-1} \neq x^n_{t-1}$.

Given that $-log(x)$ is a convex function and the denominator of our logarithm is an expectation we can apply Jensen inequality:

$$
\begin{aligned}
&\log\left(\frac{\prod_{\bar{n}\in[N],\bar{n}\neq n} p(x_t^{\bar{n}}|x_{t-1})}{\sum_{\bar{x}_{t-1}^n}\prod_{\bar{n}\in[N],\bar{n}\neq n} p(x_t^{\bar{n}}|\bar{x}_{t-1})p(\bar{x}_{t-1}^n|x_{[0:t-1]}^{\setminus n})}\right) \\
&= -\log\left(\sum_{\bar{x}_{t-1}^n} p(\bar{x}_{t-1}^n|x_{[0:t-1]}^{\setminus n})\frac{\prod_{\bar{n}\in[N],\bar{n}\neq n} p(x_t^{\bar{n}}|\bar{x}_{t-1})}{\prod_{\bar{n}\in[N],\bar{n}\neq n} p(x_t^{\bar{n}}|x_{t-1})}\right) \\
&\leq -\sum_{\bar{x}_{t-1}^n} p(\bar{x}_{t-1}^n|x_{[0:t-1]}^{\setminus n})\log\left(\prod_{\bar{n}\in[N],\bar{n}\neq n}\frac{p(x_t^{\bar{n}}|\bar{x}_{t-1})}{p(x_t^{\bar{n}}|x_{t-1})}\right) \\
&= \sum_{\bar{x}_{t-1}^n} p(\bar{x}_{t-1}^n|x_{[0:t-1]}^{\setminus n})\log\left(\prod_{\bar{n}\in[N],\bar{n}\neq n}\frac{p(x_t^{\bar{n}}|x_{t-1})}{p(x_t^{\bar{n}}|\bar{x}_{t-1})}\right).
\end{aligned}
$$

Joining (A3) with the above yields to:

$$
\begin{aligned}
\mathbf{KL}(p(\mathbf{y}_{[T]}^n)||\tilde{p}(\mathbf{y}_{[T]}^n)) \leq \sum_{t\in[T]}\sum_{x_{[0:t-1]},x_t^{\setminus n}} p(x_{[0:t-1]},x_t^{\setminus n})&\left[\sum_{\bar{x}_{t-1}^n} p(\bar{x}_{t-1}^n|x_{[0:t-1]}^{\setminus n})\right. \\
&\left.\log\left(\prod_{\bar{n}\in[N],\bar{n}\neq n}\frac{p(x_t^{\bar{n}}|x_{t-1})}{p(x_t^{\bar{n}}|\bar{x}_{t-1})}\right)\right] \\
= \sum_{t\in[T]}\sum_{x_{[0:t-1]},x_t^{\setminus n}} p(x_{[0:t-1]},x_t^{\setminus n})\left[\sum_{\bar{x}_{t-1}^n} p(\bar{x}_{t-1}^n|x_{[0:t-1]}^{\setminus n})\sum_{\bar{n}\in[N],\bar{n}\neq n}\log\left(\frac{p(x_t^{\bar{n}}|x_{t-1})}{p(x_t^{\bar{n}}|\bar{x}_{t-1})}\right)\right].&
\end{aligned}
$$

Similarly to what we did with the time steps sum, we can move the sum over the dimensions $\bar{n}$ and exchange the order with $\sum_{x_t^{\setminus n}}$, this will allow us to have a dependence on

$\bar{n}$ and so simplify $p(x_t^{\setminus n}|x_{t-1})$ to $p(x_t^{\bar{n}}|x_{t-1})$:

$$
\begin{aligned}
&\mathbf{KL}(p(\mathbf{y}^n_{[T]})||\tilde{p}(\mathbf{y}^n_{[T]})) \\
&\leq \sum_{t\in[T]} \sum_{x_{[0:t-1]},x_t^{\setminus n}} p(x_{[0:t-1]}, x_t^{\setminus n}) \left[\sum_{\bar{x}^n_{t-1}} p(\bar{x}^n_{t-1}|x^{\setminus n}_{[0:t-1]}) \sum_{\bar{n}\in[N],\bar{n}\neq n} \log\left(\frac{p(x_t^{\bar{n}}|x_{t-1})}{p(x_t^{\bar{n}}|\bar{x}_{t-1})} \right) \right] \\
&= \sum_{t\in[T]} \sum_{x_{[0:t-1]},x_t^{\setminus n}} p(x_{[0:t-1]}) p(x_t^{\setminus n}|x_{t-1}) \left[\sum_{\bar{x}^n_{t-1}} p(\bar{x}^n_{t-1}|x^{\setminus n}_{[0:t-1]}) \sum_{\bar{n}\in[N],\bar{n}\neq n} \log\left(\frac{p(x_t^{\bar{n}}|x_{t-1})}{p(x_t^{\bar{n}}|\bar{x}_{t-1})} \right) \right] \\
&= \sum_{t\in[T]} \sum_{x_{[0:t-1]}} \sum_{\bar{x}^n_{t-1}} p(\bar{x}^n_{t-1}|x^{\setminus n}_{[0:t-1]}) p(x_{[0:t-1]}) \left[\sum_{x_t^{\setminus n}} p(x_t^{\setminus n}|x_{t-1}) \sum_{\bar{n}\in[N],\bar{n}\neq n} \log\left(\frac{p(x_t^{\bar{n}}|x_{t-1})}{p(x_t^{\bar{n}}|\bar{x}_{t-1})} \right) \right] \\
&= \sum_{t\in[T]} \sum_{x_{[0:t-1]}} \sum_{\bar{x}^n_{t-1}} p(\bar{x}^n_{t-1}|x^{\setminus n}_{[0:t-1]}) p(x_{[0:t-1]}) \sum_{\bar{n}\in[N],\bar{n}\neq n} \left[\sum_{x_t^{\bar{n}}} p(x_t^{\bar{n}}|x_{t-1}) \log\left(\frac{p(x_t^{\bar{n}}|x_{t-1})}{p(x_t^{\bar{n}}|\bar{x}_{t-1})} \right) \right] \\
&= \sum_{t\in[T]} \sum_{x_{[0:t-1]}} \sum_{\bar{x}^n_{t-1}} p(\bar{x}^n_{t-1}|x^{\setminus n}_{[0:t-1]}) p(x_{[0:t-1]}) \sum_{\bar{n}\in[N],\bar{n}\neq n} \mathbf{KL}\left(p(\mathbf{x}_t^{\bar{n}}|\mathbf{x}_{t-1}) || p(\mathbf{x}_t^{\bar{n}}|\bar{\mathbf{x}}_{t-1}) \right).
\end{aligned}
$$

We can now use Corollary 3 and get:

$$
\begin{aligned}
&\mathbf{KL}(p(\mathbf{y}^n_{[T]})||\tilde{p}(\mathbf{y}^n_{[T]})) \\
&\leq \sum_{t\in[T]} \sum_{x_{[0:t-1]}} \sum_{\bar{x}^n_{t-1}} p(\bar{x}^n_{t-1}|x^{\setminus n}_{[0:t-1]}) p(x_{[0:t-1]}) \sum_{\bar{n}\in[N],\bar{n}\neq n} \mathbf{KL}\left(p(\mathbf{x}_t^{\bar{n}}|\mathbf{x}_{t-1}) || p(\mathbf{x}_t^{\bar{n}}|\bar{\mathbf{x}}_{t-1}) \right) \\
&\leq \sum_{t\in[T]} \sum_{x_{[0:t-1]}} \sum_{\bar{x}^n_{t-1}} p(\bar{x}^n_{t-1}|x^{\setminus n}_{[0:t-1]}) p(x_{[0:t-1]}) \\
&\qquad \sum_{\bar{n}\in[N],\bar{n}\neq n} \frac{1}{2} \left[\sum_{x_t^{\bar{n}}} p(x_t^{\bar{n}}|x_{t-1}) \frac{1}{p(x_t^{\bar{n}}|x_{t-1})^2} \left(p(x_t^{\bar{n}}|x_{t-1}) - p(x_t^{\bar{n}}|\bar{x}_{t-1}) \right)^2 \right] \\
&\qquad\qquad + \frac{1}{3} \left[\sum_{x_t^{\bar{n}}} p(x_t^{\bar{n}}|x_{t-1}) \frac{1}{p(x_t^{\bar{n}}|\bar{x}_{t-1})^3} \left| p(x_t^{\bar{n}}|x_{t-1}) - p(x_t^{\bar{n}}|\bar{x}_{t-1}) \right|^3 \right].
\end{aligned}
$$

We can then bound the differences between the kernels with our Assumption 1:

$$
\begin{aligned}
&\mathbf{KL}(p(\mathbf{y}^n_{[T]})||\tilde{p}(\mathbf{y}^n_{[T]})) \\
&\leq \sum_{t\in[T]} \sum_{x_{[0:t-1]}} \sum_{\bar{x}^n_{t-1}} p(\bar{x}^n_{t-1}|x^{\setminus n}_{[0:t-1]}) p(x_{[0:t-1]}) \\
&\qquad \sum_{\bar{n}\in[N],\bar{n}\neq n} \frac{1}{2}\left[\sum_{x^{\bar{n}}_t} p(x^{\bar{n}}_t|x_{t-1}) \frac{1}{p(x^{\bar{n}}_t|x_{t-1})^2} \left(p(x^{\bar{n}}_t|x_{t-1}) - p(x^{\bar{n}}_t|\bar{x}_{t-1})\right)^2\right] \\
&\qquad\qquad + \frac{1}{3}\left[\sum_{x^{\bar{n}}_t} p(x^{\bar{n}}_t|x_{t-1}) \frac{1}{p(x^{\bar{n}}_t|\bar{x}_{t-1})^3} \left|p(x^{\bar{n}}_t|x_{t-1}) - p(x^{\bar{n}}_t|\bar{x}_{t-1})\right|^3\right] \\
&\leq \sum_{t\in[T]} \sum_{x_{[0:t-1]}} \sum_{\bar{x}^n_{t-1}} p(\bar{x}^n_{t-1}|x^{\setminus n}_{[0:t-1]}) p(x_{[0:t-1]}) \\
&\qquad \sum_{\bar{n}\in[N],\bar{n}\neq n} \frac{1}{2}\left[\sum_{x^{\bar{n}}_t} p(x^{\bar{n}}_t|x_{t-1}) \frac{1}{p(x^{\bar{n}}_t|x_{t-1})^2} \frac{1}{N^2} \left|d_{n,\bar{n}}\left(x^n_{t-1}\right) - d_{n,\bar{n}}\left(\bar{x}^n_{t-1}\right)\right|^2\right] \\
&\qquad + \frac{1}{3}\left[\sum_{x^{\bar{n}}_t} p(x^{\bar{n}}_t|x_{t-1}) \frac{1}{p(x^{\bar{n}}_t|\bar{x}_{t-1})^3} \frac{1}{N^3} \left|d_{n,\bar{n}}\left(x^n_{t-1}\right) - d_{n,\bar{n}}\left(\bar{x}^n_{t-1}\right)\right|^3\right],
\end{aligned}
$$

and Assumption 2:

$$
\begin{aligned}
&\mathbf{KL}(p(\mathbf{y}^n_{[T]})||\tilde{p}(\mathbf{y}^n_{[T]})) \\
&\leq \sum_{t\in[T]} \sum_{x_{[0:t-1]}} \sum_{\bar{x}^n_{t-1}} p(\bar{x}^n_{t-1}|x^{\backslash n}_{[0:t-1]})p(x_{[0:t-1]}) \\
&\qquad \sum_{\bar{n}\in[N],\bar{n}\neq n} \frac{1}{2}\frac{1}{N^2}\left|d_{n,\bar{n}}\left(x^n_{t-1}\right) - d_{n,\bar{n}}\left(\bar{x}^n_{t-1}\right)\right|^2 \left[\sum_{x^{\bar{n}}_t} p(x^{\bar{n}}_t|x_{t-1})\frac{1}{p(x^{\bar{n}}_t|x_{t-1})^2}\right] \\
&\qquad + \frac{1}{3}\frac{1}{N^3}\left|d_{n,\bar{n}}\left(x^n_{t-1}\right) - d_{n,\bar{n}}\left(\bar{x}^n_{t-1}\right)\right|^3 \left[\sum_{x^{\bar{n}}_t} p(x^{\bar{n}}_t|x_{t-1})\frac{1}{p(x^{\bar{n}}_t|\bar{x}_{t-1})^3}\right] \\
&\leq \sum_{t\in[T]} \sum_{x_{[0:t-1]}} \sum_{\bar{x}^n_{t-1}} p(\bar{x}^n_{t-1}|x^{\backslash n}_{[0:t-1]})p(x_{[0:t-1]}) \\
&\qquad \sum_{\bar{n}\in[N],\bar{n}\neq n} \frac{1}{2}\frac{1}{\epsilon^2 N^2}\left|d_{n,\bar{n}}\left(x^n_{t-1}\right) - d_{n,\bar{n}}\left(\bar{x}^n_{t-1}\right)\right|^2 + \frac{1}{3}\frac{1}{\epsilon^3 N^3}\left|d_{n,\bar{n}}\left(x^n_{t-1}\right) - d_{n,\bar{n}}\left(\bar{x}^n_{t-1}\right)\right|^3 \\
&\leq \sum_{t\in[T]} \sum_{x_{[0:t-1]}} \sum_{\bar{x}^n_{t-1}} p(\bar{x}^n_{t-1}|x^{\backslash n}_{[0:t-1]})p(x_{[0:t-1]}) \\
&\qquad \sum_{\bar{n}\in[N],\bar{n}\neq n} \left[\frac{1}{2}\frac{1}{\epsilon^2 N} + \frac{1}{3}\frac{1}{\epsilon^3 N}\right]\frac{1}{N}\left|d_{n,\bar{n}}\left(x^n_{t-1}\right) - d_{n,\bar{n}}\left(\bar{x}^n_{t-1}\right)\right|^2
\end{aligned}
$$

where in the last step we used $\left|d_{n,\bar{n}}\left(x^n_{t-1}\right) - d_{n,\bar{n}}\left(\bar{x}^n_{t-1}\right)\right| < N$. We can then reformulate:

$$
\begin{aligned}
&\mathbf{KL}(p(\mathbf{y}^n_{[T]})||\tilde{p}(\mathbf{y}^n_{[T]})) \\
&\leq \sum_{t\in[T]} \sum_{x_{[0:t-1]}} \sum_{\bar{x}^n_{t-1}} p(\bar{x}^n_{t-1}|x^{\backslash n}_{[0:t-1]})p(x_{[0:t-1]}) \\
&\qquad \sum_{\bar{n}\in[N],\bar{n}\neq n} \left[\frac{1}{2}\frac{1}{\epsilon^2 N} + \frac{1}{3}\frac{1}{\epsilon^3 N}\right]\frac{1}{N}\left|d_{n,\bar{n}}\left(x^n_{t-1}\right) - d_{n,\bar{n}}\left(\bar{x}^n_{t-1}\right)\right|^2 \\
&\leq \left[\frac{1}{2}\frac{1}{\epsilon^2 N} + \frac{1}{3}\frac{1}{\epsilon^3 N}\right] \sum_{t\in[T]} \sum_{\bar{n}\in[N],\bar{n}\neq n} \frac{1}{N} \\
&\qquad \sum_{x_{[0:t-1]}} \sum_{\bar{x}^n_{t-1}} p(\bar{x}^n_{t-1}|x^{\backslash n}_{[0:t-1]})p(x_{[0:t-1]})\left|d_{n,\bar{n}}\left(x^n_{t-1}\right) - d_{n,\bar{n}}\left(\bar{x}^n_{t-1}\right)\right|^2.
\end{aligned}
$$

Now we need to work on:

$$
\sum_{x_{[0:t-1]}} \sum_{\bar{x}^n_{t-1}} p(\bar{x}^n_{t-1}|x^{\backslash n}_{[0:t-1]})p(x_{[0:t-1]})\left|d_{n,\bar{n}}\left(x^n_{t-1}\right) - d_{n,\bar{n}}\left(\bar{x}^n_{t-1}\right)\right|^2,
$$

which can be reformulated by expanding the square:

$$\sum_{x_{[0:t-1]}} \sum_{\bar{x}^n_{t-1}} p(\bar{x}^n_{t-1}|x^{\setminus n}_{[0:t-1]}) p(x_{[0:t-1]}) \left(d_{n,\bar{n}} \left(x^n_{t-1} \right)^2 - 2 d_{n,\bar{n}} \left(x^n_{t-1} \right) d_{n,\bar{n}} \left(\bar{x}^n_{t-1} \right) + d_{n,\bar{n}} \left(\bar{x}^n_{t-1} \right)^2 \right),$$

and we can work on three terms inside the parenthesis separately. Firstly, we have:

$$\begin{aligned}
\sum_{x_{[0:t-1]}} \sum_{\bar{x}^n_{t-1}} p(\bar{x}^n_{t-1}|x^{\setminus n}_{[0:t-1]}) p(x_{[0:t-1]}) d_{n,\bar{n}} \left(x^n_{t-1} \right)^2 &= \mathbb{E}\left[d_{n,\bar{n}} \left(\mathbf{x}^n_{t-1} \right)^2 \right] \\
&= \mathbb{E}\left\{ \mathbb{E}\left[d_{n,\bar{n}} \left(\mathbf{x}^n_{t-1} \right)^2 \middle| \mathbf{x}^{\setminus n}_{[0:t-1]} \right] \right\},
\end{aligned}$$

as we have no dependence on $\bar{x}^n_{t-1}$ and where the last step follows from the law of total expectations. Now we look at the other squared term:

$$\begin{aligned}
&\sum_{x_{[0:t-1]}} \sum_{\bar{x}^n_{t-1}} p(\bar{x}^n_{t-1}|x^{\setminus n}_{[0:t-1]}) p(x_{[0:t-1]}) d_{n,\bar{n}} \left(\bar{x}^n_{t-1} \right)^2 \\
&= \sum_{x^{\setminus n}_{[0:t-1]}} \sum_{\bar{x}^n_{t-1}} p(\bar{x}^n_{t-1}|x^{\setminus n}_{[0:t-1]}) p(x^{\setminus n}_{[0:t-1]}) d_{n,\bar{n}} \left(\bar{x}^n_{t-1} \right)^2 = \mathbb{E}\left\{ \mathbb{E}\left[d_{n,\bar{n}} \left(\mathbf{x}^n_{t-1} \right)^2 \middle| \mathbf{x}^{\setminus n}_{[0:t-1]} \right] \right\},
\end{aligned}$$

as again we have no dependence on $x^n_{[0:t-1]}$ and so we can marginilise them out from $p(x_{[0:t-1]})$. There is only the cross term left, and try to reformulate it as an expectation:

$$\begin{aligned}
&\sum_{x_{[0:t-1]}} \sum_{\bar{x}^n_{t-1}} p(\bar{x}^n_{t-1}|x^{\setminus n}_{[0:t-1]}) p(x_{[0:t-1]}) d_{n,\bar{n}} \left(x^n_{t-1} \right) d_{n,\bar{n}} \left(\bar{x}^n_{t-1} \right) \\
&= \sum_{x_{[0:t-2]}} \sum_{x^{\setminus n}_{t-1}} \sum_{x^n_{t-1}} \sum_{\bar{x}^n_{t-1}} p(x_{[0:t-1]}) p(\bar{x}^n_{t-1}|x^{\setminus n}_{[0:t-1]}) d_{n,\bar{n}} \left(x^n_{t-1} \right) d_{n,\bar{n}} \left(\bar{x}^n_{t-1} \right) \\
&= \sum_{x^{\setminus n}_{[0:t-2]}} \sum_{x^{\setminus n}_{t-1}} \sum_{x^n_{t-1}} \sum_{\bar{x}^n_{t-1}} p(x^{\setminus n}_{[0:t-2]}, x_{t-1}) p(\bar{x}^n_{t-1}|x^{\setminus n}_{[0:t-1]}) d_{n,\bar{n}} \left(x^n_{t-1} \right) d_{n,\bar{n}} \left(\bar{x}^n_{t-1} \right) \\
&= \sum_{x^{\setminus n}_{[0:t-1]}} p(x^{\setminus n}_{[0:t-1]}) \sum_{x^n_{t-1}} \sum_{\bar{x}^n_{t-1}} p(x^n_{t-1}|x^{\setminus n}_{[0:t-1]}) p(\bar{x}^n_{t-1}|x^{\setminus n}_{[0:t-1]}) d_{n,\bar{n}} \left(x^n_{t-1} \right) d_{n,\bar{n}} \left(\bar{x}^n_{t-1} \right) \\
&= \sum_{x^{\setminus n}_{[0:t-1]}} p(x^{\setminus n}_{[0:t-1]}) \left[\sum_{x^n_{t-1}} p(x^n_{t-1}|x^{\setminus n}_{[0:t-1]}) d_{n,\bar{n}} \left(x^n_{t-1} \right) \right] \left[\sum_{\bar{x}^n_{t-1}} p(\bar{x}^n_{t-1}|x^{\setminus n}_{[0:t-1]}) d_{n,\bar{n}} \left(\bar{x}^n_{t-1} \right) \right] \\
&= \sum_{x^{\setminus n}_{[0:t-1]}} p(x^{\setminus n}_{[0:t-1]}) \mathbb{E}\left[d_{n,\bar{n}} \left(\mathbf{x}^n_{t-1} \right) \middle| \mathbf{x}^{\setminus n}_{[0:t-1]} \right]^2 = \mathbb{E}\left\{ \mathbb{E}\left[d_{n,\bar{n}} \left(\mathbf{x}^n_{t-1} \right) \middle| \mathbf{x}^{\setminus n}_{[0:t-1]} \right]^2 \right\}.
\end{aligned}$$

From the three reformulations above it follows that:

$$
\begin{aligned}
&\mathbf{KL}(p(\mathbf{y}^n_{[T]})||\tilde{p}(\mathbf{y}^n_{[T]}))\\
&\leq \left[\frac{1}{2\epsilon^2 N} + \frac{1}{3\epsilon^3 N}\right]\\
&\quad \sum_{t\in[T]} \sum_{\bar{n}\in[N],\bar{n}\neq n} \frac{1}{N} \sum_{x_{[0:t-1]}} \sum_{\bar{x}^n_{t-1}} p(\bar{x}^n_{t-1}|x^{\setminus n}_{[0:t-1]})p(x_{[0:t-1]}) \left|d_{n,\bar{n}}\left(x^n_{t-1}\right) - d_{n,\bar{n}}\left(\bar{x}^n_{t-1}\right)\right|^2\\
&\leq \left[\frac{1}{2\epsilon^2 N} + \frac{1}{3\epsilon^3 N}\right]\\
&\quad \sum_{t\in[T]} \sum_{\bar{n}\in[N],\bar{n}\neq n} \frac{1}{N} \left[2\mathbb{E}\left\{\mathbb{E}\left[d_{n,\bar{n}}\left(\mathbf{x}^n_{t-1}\right)^2 \middle| \mathbf{x}^{\setminus n}_{[0:t-1]}\right]\right\} - 2\mathbb{E}\left\{\mathbb{E}\left[d_{n,\bar{n}}\left(\mathbf{x}^n_{t-1}\right) \middle| \mathbf{x}^{\setminus n}_{[0:t-1]}\right]^2\right\}\right]\\
&\leq 2\left[\frac{1}{2\epsilon^2 N} + \frac{1}{3\epsilon^3 N}\right] \sum_{\bar{n}\in[N],\bar{n}\neq n} \frac{1}{N} \left[\sum_{t\in[T]} \mathbb{E}\left\{\mathbb{V}ar\left[d_{n,\bar{n}}\left(\mathbf{x}^n_{t-1}\right) \middle| \mathbf{x}^{\setminus n}_{[0:t-1]}\right]\right\}\right]\\
&\leq 2\left[\frac{1}{2\epsilon^2 N} + \frac{1}{3\epsilon^3 N}\right] \sum_{t\in[T]} \mathbb{E}\left\{\frac{1}{N} \sum_{\bar{n}\in[N],\bar{n}\neq n} \mathbb{V}ar\left[d_{n,\bar{n}}\left(\mathbf{x}^n_{t-1}\right) \middle| \mathbf{x}^{\setminus n}_{[0:t-1]}\right]\right\},
\end{aligned}
$$

which completes the proof.

□

A.3 KL bounds for SimBa-CL for general partitions

As explained in the main paper we can generalize SimBa-CL with and without feedback to work on different factorizations of the dimensions. Precisely, if we have a general partition $\mathcal{K}$ of $[N]$, then we can define for each $K \in \mathcal{K}$:

$$
\begin{aligned}
p\left(y^K_{[T]}\right) &:= \sum_{x_{[0:T]}} p\left(x^{\setminus K}_{[0:T]}\right) p\left(x^K_{[0:T]}|x^{\setminus K}_{[0:T]}\right) \prod_{t\in[T]} \prod_{n\in K} p\left(y^n_t|x^n_t\right);\\
\tilde{p}_{\mathcal{K}}\left(y^K_{[T]}\right) &:= \sum_{x_{[0:T]}} p\left(x^{\setminus K}_{[0:T]}\right) \prod_{n\in K} p\left(x^n_0\right) \prod_{t\in[T]} p\left(x^n_t|x_{t-1}\right) p\left(y^n_t|x^n_t\right),
\end{aligned}
$$

and then combine them as $p_{\mathcal{K}}(y_{[T]}) := \prod_{K\in\mathcal{K}} p\left(y^K_{[T]}\right)$ and as $\tilde{p}_{\mathcal{K}}(y_{[T]}) := \prod_{K\in\mathcal{K}} \tilde{p}_{\mathcal{K}}\left(y^K_{[T]}\right)$ to provide an approximation of our likelihood $p(y_{[T]})$. In this case, we are computing the marginals on subsets of the state-space, which can be particularly suited when we expect strong dependence inside the elements of the partition, e.g. an agent-based model with individuals partitioned in households.

Again $p_{\mathcal{K}}(y_{[T]})$ can be seen as a SimBa-CL with feedback, where the likelihood is approximated with the product of the true marginals, and $\tilde{p}_{\mathcal{K}}(y_{[T]})$ can be seen as a SimBa-CL without feedback. As for the fully factorize case, we have a recursive

formula for $p(x^K_{[0:T]}|x^{\backslash K}_{[0:T]})$ given by:

$$p(x^K_{[0:T]}|x^{\backslash K}_{[0:T]}) = \prod_{n\in K} p(x^n_0) \prod_{t\in[T]} p(x^n_t|x_{t-1}) f_K(x^K_{t-1}, x^{\backslash K}_{[0:t]}),$$

where the simulation feedback is now:

$$f_K(x^K_{t-1}, x^{\backslash K}_{[0:t]}) := \frac{\prod_{\bar{n}\in[N]\backslash K} p(x^{\bar{n}}_t|x_{t-1})}{\sum_{\bar{x}^K_{t-1}} \prod_{\bar{n}\in[N]\backslash K} p(x^{\bar{n}}_t|\bar{x}^K_{t-1}, x^{\backslash K}_{[t-1]}) p(\bar{x}^K_{t-1}|x^{\backslash K}_0, x^{\backslash K}_{[t-1]})},$$

As for SimBa-CL with and without feedback, with these general terms referring to the fully factorized case, we can assess the degradation of approximation quality when excluding the feedback from the procedure. Naturally, we need a more general version of the previous assumptions, given that we are now dealing with changes across multiple dimensions. The assumptions have already been reported before and can be found in Assumption 3 and Assumption 4, however, we report them again here and comment on the interpretation.

Assumption 5. *For any* $K \in \mathcal{K}$ *and for any* $x^{\bar{n}}_t \in \mathcal{X}$ *with* $\bar{n} \notin K$, *if* $x_{t-1}, \bar{x}_{t-1} \in \mathcal{X}^N$ *are such that* $x^{\backslash K}_{t-1} = \bar{x}^{\backslash K}_{t-1}$ *then:*

$$\left|p\left(x^{\bar{n}}_t|x_{t-1}\right) - p\left(x^{\bar{n}}_t|\bar{x}_{t-1}\right)\right| \leq \frac{1}{N}\left|d_{K,\bar{n}}\left(x^n_{t-1}\right) - d_{K,\bar{n}}\left(\bar{x}^n_{t-1}\right)\right|.$$

where $d_{K,\bar{n}} : \mathcal{X}^K \to \mathbb{R}_+$.

Assumption 6. *For any* $K, \bar{K} \in \mathcal{K}$, *if* $x_{t-1}, \bar{x}_{t-1} \in \mathcal{X}^N$ *are such that* $x^{\backslash \bar{K}}_{t-1} = \bar{x}^{\backslash \bar{K}}_{t-1}$ *then there exists* $0 < \epsilon < 1$ *such that:*

$$\sum_{x^n_t} p\left(x^n_t|x_{t-1}\right) \frac{1}{p\left(x^n_t|\bar{x}_{t-1}\right)^2} \leq \frac{1}{\epsilon^2}, \quad \text{and} \quad \sum_{x^n_t} p\left(x^n_t|x_{t-1}\right) \frac{1}{p\left(x^n_t|\bar{x}_{t-1}\right)^3} \leq \frac{1}{\epsilon^3}.$$

Assumption 5 and Assumption 6 provide the required generalizations. Assumption 5 is measuring the interactions on $K \in \mathcal{K}$ and ensuring that changes on K have a limited impact on the dynamic outside K. Meanwhile, Assumption 6 formulates a stronger version of Assumption 2.

As for the fully factorized case, given the assumptions, we can bound the **KL**-divergence between our SimBa-CL with and without feedback on general partitions. As it can be noticed from the statement of Theorem 4, there are a couple of deviations from the fully factorize case.

Theorem 4. *If* $\left|d_{K,\bar{n}}\left(x^K\right) - d_{K,\bar{n}}\left(\bar{x}^K\right)\right| < N$ *for any* $x^K, \bar{x}^K \in \mathcal{X}^K$ *and for any* $K \in \mathcal{K}$, *and assumptions 6-5 hold, then for any* $K \in \mathcal{K}$*:*

$$\mathbf{KL}\left[p\left(\mathbf{y}^K_{[T]}\right) || \tilde{p}_{\mathcal{K}}\left(\mathbf{y}^K_{[T]}\right)\right] \leq \frac{a(\epsilon)}{N} \sum_{t\in[T]} \mathbb{E}\left\{\frac{1}{N} \sum_{\bar{n}\in[N]\backslash K} \mathbb{V}ar\left[d_{K,\bar{n}}\left(\mathbf{x}^K_{t-1}\right) \middle| \mathbf{x}^{\backslash K}_{[0:t-1]}\right]\right\},$$

where $a(\epsilon) := 2\left[\frac{1}{2\epsilon^2} + \frac{1}{3\epsilon^3}\right]$.

Once more, we can interpret the bound as the one from Theorem 1, suggesting to exclude the feedback whenever the dimension N is large and when the process noise is contained.

Given assumptions 3-4 we can prove bounds on the KL-divergence between the with feedback case and the without feedback case.

proof of Theorem 5. The proof of Theorem 2 follows the same steps as the proof of Theorem 1, with K instead of n. However, the last steps are slightly different due to a different assumption to apply. We report them here for completeness.

Precisely after proving that our KL is well-defined, applying the data processing inequality, exploiting the factorizations in the model, and applying Jensen, we reach the point of using Corollary 3 and get:

$$
\begin{aligned}
&\mathbf{KL}(p(\mathbf{y}^K_{[T]})||\tilde{p}(\mathbf{y}^K_{[T]})) \\
&\leq \sum_{t\in[T]} \sum_{x_{[0:t-1]}} \sum_{\bar{x}^K_{t-1}} p(\bar{x}^K_{t-1}|x^{\setminus K}_{[0:t-1]})p(x_{[0:t-1]}) \sum_{\bar{n}\in[N]\setminus K} \mathbf{KL}\left(p(\mathbf{x}^{\bar{n}}_t|\mathbf{x}_{t-1})||p(\mathbf{x}^{\bar{n}}_t|\bar{\mathbf{x}}_{t-1})\right) \\
&\leq \sum_{t\in[T]} \sum_{x_{[0:t-1]}} \sum_{\bar{x}^K_{t-1}} p(\bar{x}^K_{t-1}|x^{\setminus K}_{[0:t-1]})p(x_{[0:t-1]}) \\
&\qquad \sum_{\bar{n}\in[N]\setminus K} \frac{1}{2}\left[\sum_{x^{\bar{n}}_t} p(x^{\bar{n}}_t|x_{t-1})\frac{1}{p(x^{\bar{n}}_t|x_{t-1})^2}\left(p(x^{\bar{n}}_t|x_{t-1}) - p(x^{\bar{n}}_t|\bar{x}_{t-1})\right)^2\right] \\
&\qquad\qquad + \frac{1}{3}\left[\sum_{x^{\bar{n}}_t} p(x^{\bar{n}}_t|x_{t-1})\frac{1}{p(x^{\bar{n}}_t|\bar{x}_{t-1})^3}\left|p(x^{\bar{n}}_t|x_{t-1}) - p(x^{\bar{n}}_t|\bar{x}_{t-1})\right|^3\right].
\end{aligned}
$$

We can then bound the differences between the kernels with our Assumption 3:

$$
\begin{aligned}
&\mathbf{KL}(p(\mathbf{y}^K_{[T]})||\tilde{p}(\mathbf{y}^K_{[T]})) \\
&\leq \sum_{t\in[T]} \sum_{x_{[0:t-1]}} \sum_{\bar{x}^K_{t-1}} p(\bar{x}^K_{t-1}|x^{\setminus K}_{[0:t-1]})p(x_{[0:t-1]}) \\
&\qquad \sum_{\bar{n}\in[N]\setminus K} \frac{1}{2}\left[\sum_{x^{\bar{n}}_t} p(x^{\bar{n}}_t|x_{t-1})\frac{1}{p(x^{\bar{n}}_t|x_{t-1})^2}\frac{1}{N^2}\left|d_{K,\bar{n}}\left(x^K_{t-1}\right) - d_{K,\bar{n}}\left(\bar{x}^K_{t-1}\right)\right|^2\right] \\
&\qquad + \frac{1}{3}\left[\sum_{x^{\bar{n}}_t} p(x^{\bar{n}}_t|x_{t-1})\frac{1}{p(x^{\bar{n}}_t|\bar{x}_{t-1})^3}\frac{1}{N^3}\left|d_{K,\bar{n}}\left(x^K_{t-1}\right) - d_{K,\bar{n}}\left(\bar{x}^K_{t-1}\right)\right|^3\right],
\end{aligned}
$$

and Assumption 4:

$$
\begin{aligned}
&\mathbf{KL}(p(\mathbf{y}^K_{[T]})||\tilde{p}(\mathbf{y}^K_{[T]})) \\
&\leq \sum_{t\in[T]} \sum_{x_{[0:t-1]}} \sum_{\bar{x}^K_{t-1}} p(\bar{x}^K_{t-1}|x^{\backslash K}_{[0:t-1]})p(x_{[0:t-1]}) \\
&\qquad \sum_{\bar{n}\in[N]\backslash K} \frac{1}{2}\frac{1}{\epsilon^2 N^2}\left|d_{K,\bar{n}}\left(x^K_{t-1}\right) - d_{K,\bar{n}}\left(\bar{x}^K_{t-1}\right)\right|^2 + \frac{1}{3}\frac{1}{\epsilon^3 N^3}\left|d_{K,\bar{n}}\left(x^K_{t-1}\right) - d_{K,\bar{n}}\left(\bar{x}^K_{t-1}\right)\right|^3 \\
&\leq \sum_{t\in[T]} \sum_{x_{[0:t-1]}} \sum_{\bar{x}^K_{t-1}} p(\bar{x}^K_{t-1}|x^{\backslash K}_{[0:t-1]})p(x_{[0:t-1]}) \\
&\qquad \sum_{\bar{n}\in[N]\backslash K} \left[\frac{1}{2}\frac{1}{\epsilon^2 N} + \frac{1}{3}\frac{1}{\epsilon^3 N}\right]\frac{1}{N}\left|d_{K,\bar{n}}\left(x^K_{t-1}\right) - d_{K,\bar{n}}\left(\bar{x}^K_{t-1}\right)\right|^2
\end{aligned}
$$

where in the last step we used $\left|d_{K,\bar{n}}\left(x^K_{t-1}\right) - d_{K,\bar{n}}\left(\bar{x}^K_{t-1}\right)\right| < N$. Similarly to the proof of Theorem 1, we can reformulate in terms of expected variance but this time we will work on $d_{K,\bar{n}}(\mathbf{x}^K_{t-1})$ and so:

$$
\begin{aligned}
&\mathbf{KL}(p(\mathbf{y}^K_{[T]})||\tilde{p}(\mathbf{y}^K_{[T]})) \\
&\leq \left[\frac{1}{2\epsilon^2 N} + \frac{1}{3\epsilon^3 N}\right] \\
&\qquad \sum_{t\in[T]} \sum_{\bar{n}\in[N]\backslash K} \frac{1}{N} \sum_{x_{[0:t-1]}} \sum_{\bar{x}^K_{t-1}} p(\bar{x}^K_{t-1}|x^{\backslash K}_{[0:t-1]})p(x_{[0:t-1]})\left|d_{K,\bar{n}}\left(x^K_{t-1}\right) - d_{K,\bar{n}}\left(\bar{x}^K_{t-1}\right)\right|^2 \\
&\leq \left[\frac{1}{2\epsilon^2 N} + \frac{1}{3\epsilon^3 N}\right] \\
&\qquad \sum_{t\in[T]} \sum_{\bar{n}\in[N]\backslash K} \frac{1}{N}\left[2\mathbb{E}\left\{\mathbb{E}\left[d_{K,\bar{n}}\left(\mathbf{x}^K_{t-1}\right)^2 \middle| \mathbf{x}^{\backslash K}_{[0:t-1]}\right]\right\} - 2\mathbb{E}\left\{\mathbb{E}\left[d_{K,\bar{n}}\left(\mathbf{x}^K_{t-1}\right) \middle| \mathbf{x}^{\backslash K}_{[0:t-1]}\right]^2\right\}\right] \\
&\leq 2\left[\frac{1}{2\epsilon^2 N} + \frac{1}{3\epsilon^3 N}\right] \sum_{\bar{n}\in[N]\backslash K} \frac{1}{N}\left[\sum_{t\in[T]} \mathbb{E}\left\{\mathbb{V}ar\left[d_{K,\bar{n}}\left(\mathbf{x}^K_{t-1}\right) \middle| \mathbf{x}^{\backslash K}_{[0:t-1]}\right]\right\}\right] \\
&\leq 2\left[\frac{1}{2\epsilon^2 N} + \frac{1}{3\epsilon^3 N}\right] \sum_{t\in[T]} \mathbb{E}\left\{\frac{1}{N}\sum_{\bar{n}\in[N]\backslash K} \mathbb{V}ar\left[d_{K,\bar{n}}\left(\mathbf{x}^K_{t-1}\right) \middle| \mathbf{x}^{\backslash K}_{[0:t-1]}\right]\right\},
\end{aligned}
$$

which completes the proof.

□

Appendix B SimBa-CL as composite likelihood

Here we discuss further SimBa-CL as a composite likelihood method, with a particular focus on the estimation of the sensitivity matrix and variability matrix and the observed information.

B.1 Observed information

In the main paper, we reported $S(\theta)$ and $V(\theta)$ as our sensitivity matrix and variability matrix, which we defined as:

$$S(\theta) = \mathbb{E}_\theta \left\{ -\operatorname{Hess}_\theta \left[\log \tilde{p}_{\mathcal{K}} \left(\mathbf{y}_{[T]}^K | \theta \right) \right] \right\} \text{ and } V(\theta) = \mathbb{V}\mathrm{ar}_\theta \left\{ \nabla_\theta \left[\log \tilde{p}_{\mathcal{K}} \left(\mathbf{y}_{[T]}^K | \theta \right) \right] \right\}.$$

This formulation suggests a bootstrap approach where the expectations are estimated by simulating from the model and computing the Hessian and gradient of the composite likelihood in the simulated data accordingly. However, it is often common to use an observed information approach, where the observed data are plugged-in our instead of simulated data. It is worth noting that for the above formulation of variability and specificity matrix, this is not ideal as the observed data are not independent and identically distributed, meaning that we do not have a way to estimate the mean and the variance in the equations above.

In the main paper we also reported another formulation of the sensitivity matrix and variability matrix, where approximate Bartlett identities are used:

$$S(\theta) \approx \sum_{K \in \mathcal{K}} \mathbb{E}_\theta \left\{ \nabla_\theta \left[\log \tilde{p}_{\mathcal{K}} \left(\mathbf{y}_{[T]}^K | \theta \right) \right] \nabla_\theta \left[\log \tilde{p}_{\mathcal{K}} \left(\mathbf{y}_{[T]}^K | \theta \right) \right]^\top \right\},$$

$$V(\theta) \approx \sum_{K, \tilde{K} \in \mathcal{K}} \mathbb{E}_\theta \left\{ \nabla_\theta \left[\log \tilde{p}_{\mathcal{K}} \left(\mathbf{y}_{[T]}^K | \theta \right) \right] \nabla_\theta \left[\log \tilde{p}_{\mathcal{K}} \left(\mathbf{y}_{[T]}^{\tilde{K}} | \theta \right) \right]^\top \right\}.$$

These alternative formulations are more suited to the observed information approach as we could assume independence across blocks and identical distribution of the blocks and get:

$$S(\theta) \approx \sum_{K \in \mathcal{K}} \left\{ \nabla_\theta \left[\log \tilde{p}_{\mathcal{K}} \left(y_{[T]}^K | \theta \right) \right] \nabla_\theta \left[\log \tilde{p}_{\mathcal{K}} \left(y_{[T]}^K | \theta \right) \right]^\top \right\},$$

$$V(\theta) \approx \sum_{K, \tilde{K} \in \mathcal{K}} \left\{ \nabla_\theta \left[\log \tilde{p}_{\mathcal{K}} \left(y_{[T]}^K | \theta \right) \right] \nabla_\theta \left[\log \tilde{p}_{\mathcal{K}} \left(y_{[T]}^{\tilde{K}} | \theta \right) \right]^\top \right\}.$$

It is surely not surprising that this approach will not perform well when the blocks are highly correlated and different in distribution.

For completeness, we report the experiment table from the main paper where empirical coverage when learning the full model is measured. Here we add an extra row showing the resulting coverage across parameters when using the observed information. As clear from the bad coverage, the observed information might lead to overconfident

sets, this is probably due to the challenge of identifying the parameters and so the optimizer gets stuck in local maxima of the likelihood.

Parameter	β_0	β_λ	β_γ	q	ι
Without Bartlett	0.17 and 0.05	0.61 and 0.87	0.8 and 1.	0.87 and 0.5	0.02
With Bartlett	0.98 and 0.89	0.99 and 0.75	0.97 and 0.97	1. and 0.98	0.92
With Bartlett OI	0.48 and 0.30	0.51 and 0.31	0.01 and 0.06	0.62 and 0.02	0.03

Table B1: Empirical coverage per each parameter when computing the Godambe information matrix with and without the approximate Bartlett identities and when using the observed information. Whenever the parameter is bi-dimensional the coverage per each component is reported in the same cell separated by "and". "OI" refers to observed information.

B.2 Estimating the sensitivity and variability matrix

Bootstrap sampling seems like a good strategy to compute an estimate of the variability and sensitivity matrix, precisely given P samples from the model:

$$y_{[T]}^{i,} \sim p(y_{[T]}|\hat{\theta}_{CL}) \text{ for } i \in [P],$$

where $\hat{\theta}_{CL}$ is our maximum SimBa-CL estimator, can be used to get:

$$S\left(\hat{\theta}_{CL}\right) \approx \frac{1}{P} \sum_{i \in [P]} \left\{ -\operatorname{Hess}_\theta \left[\log \tilde{p}_{\mathcal{K}} \left(y_{[T]}^K | \hat{\theta}_{CL} \right) \right] \right\} \text{ and}$$

$$V\left(\hat{\theta}_{CL}\right) \approx \frac{1}{P-1} \sum_{i \in [P]} \left[\nabla_\theta \left[\log \tilde{p}_{\mathcal{K}} \left(y_{[T]}^K | \hat{\theta}_{CL} \right) \right] - \frac{1}{P} \sum_{i \in [P]} \left\{ \nabla_\theta \left[\log \tilde{p}_{\mathcal{K}} \left(y_{[T]}^K | \hat{\theta}_{CL} \right) \right] \right\} \right]^2 .$$

Similarly when invoking the approximate first and second Bartlett identities we have:

$$S\left(\hat{\theta}_{CL}\right) \approx \sum_{K \in \mathcal{K}} \frac{1}{P} \sum_{i \in [P]} \left\{ \nabla_\theta \left[\log \tilde{p}_{\mathcal{K}} \left(y_{[T]}^K | \hat{\theta}_{CL} \right) \right] \nabla_\theta \left[\log \tilde{p}_{\mathcal{K}} \left(y_{[T]}^K | \hat{\theta}_{CL} \right) \right]^\top \right\},$$

$$V\left(\hat{\theta}_{CL}\right) \approx \sum_{K, \tilde{K} \in \mathcal{K}} \frac{1}{P} \sum_{i \in [P]} \left\{ \nabla_\theta \left[\log \tilde{p}_{\mathcal{K}} \left(y_{[T]}^K | \hat{\theta}_{CL} \right) \right] \nabla_\theta \left[\log \tilde{p}_{\mathcal{K}} \left(y_{[T]}^{\tilde{K}} | \hat{\theta}_{CL} \right) \right]^\top \right\}.$$

Note that all the differentiation operations can be run in parallel on P, making the bootstrap approach relatively cheap when consistent computational resources are available.

B.3 Approximate first and second Bartlett identities

In this section, we motivate the use of the Bartlett identities in our computations. The first Bartlett identity states:

$$\mathbb{E}_\theta\left[\nabla_\theta \log\left(p\left(\mathbf{y}_{[T]}|\theta\right)\right)\right] = 0,$$

which we want to discuss for the SimBa-CL case.

For SimBa-CL we have:

$$\begin{aligned}
\mathbb{E}_\theta\left[\nabla_\theta \log\left(\tilde{p}_{\mathcal{K}}\left(\mathbf{y}_{[T]}|\theta\right)\right)\right] &= \sum_{K\in\mathcal{K}} \mathbb{E}_\theta\left[\nabla_\theta \log\left(\tilde{p}_{\mathcal{K}}\left(\mathbf{y}^K_{[T]}|\theta\right)\right)\right] = \sum_{K\in\mathcal{K}} \mathbb{E}_\theta\left[\frac{\nabla_\theta\left(\tilde{p}_{\mathcal{K}}\left(\mathbf{y}^K_{[T]}|\theta\right)\right)}{\tilde{p}_{\mathcal{K}}\left(\mathbf{y}^K_{[T]}|\theta\right)}\right] \\
&= \sum_{K\in\mathcal{K}} \sum_{y^K_{[T]}} \left[\nabla_\theta\left(\tilde{p}_{\mathcal{K}}\left(y^K_{[T]}|\theta\right)\right)\frac{p\left(y^K_{[T]}|\theta\right)}{\tilde{p}_{\mathcal{K}}\left(y^K_{[T]}|\theta\right)}\right] \\
&\approx \sum_{K\in\mathcal{K}} \sum_{y^K_{[T]}} \left[\nabla_\theta\left(\tilde{p}_{\mathcal{K}}\left(y^K_{[T]}|\theta\right)\right)\right] = 0,
\end{aligned}$$

where we use the $\tilde{p}_{\mathcal{K}}\left(y^K_{[T]}|\theta\right) \approx p\left(y^K_{[T]}|\theta\right)$, i.e. a simulation feedback close to 1, and the fact that $\tilde{p}_{\mathcal{K}}$ is still a proper probability distribution and so sum up to 1. Obviously, if we consider SimBa-CL with feedback the first Bartlett identity holds exactly cause we are targeting the true marginals.

We now discuss the second Bartlett identity:

$$\mathbb{E}_\theta\left[\text{Hess}_\theta \log\left(p\left(\mathbf{y}_{[T]}|\theta\right)\right)\right] = -\mathbb{E}_\theta\left\{\nabla_\theta\left[\log p\left(\mathbf{y}_{[T]}|\theta\right)\right]\nabla_\theta\left[\log p\left(\mathbf{y}_{[T]}|\theta\right)\right]^\top\right\}.$$

Note that for SimBa-CL we have:

$$\mathbb{E}_\theta\left[\text{Hess}_\theta \log\left(\tilde{p}_\mathcal{K}\left(\mathbf{y}_{[T]}|\theta\right)\right)\right] = \sum_{K\in\mathcal{K}} \mathbb{E}_\theta\left[\text{Hess}_\theta \log\left(\tilde{p}_\mathcal{K}\left(\mathbf{y}^K_{[T]}|\theta\right)\right)\right]$$

$$= \sum_{K\in\mathcal{K}} \mathbb{E}_\theta\left[\nabla_\theta\nabla_\theta \log\left(\tilde{p}_\mathcal{K}\left(\mathbf{y}^K_{[T]}|\theta\right)\right)\right] = \sum_{K\in\mathcal{K}} \mathbb{E}_\theta\left[\nabla_\theta\left(\frac{\nabla_\theta \tilde{p}_\mathcal{K}\left(\mathbf{y}^K_{[T]}|\theta\right)}{\tilde{p}_\mathcal{K}\left(\mathbf{y}^K_{[T]}|\theta\right)}\right)\right] =$$

$$= \sum_{K\in\mathcal{K}} \mathbb{E}_\theta\left[\left(\frac{\text{Hess}_\theta\, \tilde{p}_\mathcal{K}\left(\mathbf{y}^K_{[T]}|\theta\right)}{\tilde{p}_\mathcal{K}\left(\mathbf{y}^K_{[T]}|\theta\right)}\right) - \left(\frac{\nabla_\theta \tilde{p}_\mathcal{K}\left(\mathbf{y}^K_{[T]}|\theta\right)\nabla_\theta \tilde{p}_\mathcal{K}\left(\mathbf{y}^K_{[T]}|\theta\right)^\top}{\tilde{p}_\mathcal{K}\left(\mathbf{y}^K_{[T]}|\theta\right)^2}\right)\right]$$

$$= \sum_{K\in\mathcal{K}}\sum_{y^K_{[T]}} \left[\left(\frac{\text{Hess}_\theta\, \tilde{p}_\mathcal{K}\left(y^K_{[T]}|\theta\right)}{\tilde{p}_\mathcal{K}\left(y^K_{[T]}|\theta\right)}\right) - \left(\nabla_\theta \log \tilde{p}_\mathcal{K}\left(y^K_{[T]}|\theta\right)\nabla_\theta \log \tilde{p}_\mathcal{K}\left(y^K_{[T]}|\theta\right)^\top\right)\right] p(y^K_{[T]}|\theta)$$

$$\approx -\sum_{K\in\mathcal{K}}\sum_{y^K_{[T]}} \nabla_\theta\left[\log \tilde{p}_\mathcal{K}\left(y^K_{[T]}|\theta\right)\right]\nabla_\theta\left[\log \tilde{p}_\mathcal{K}\left(y^K_{[T]}|\theta\right)\right]^\top p(y^K_{[T]}|\theta)$$

$$= -\sum_{K\in\mathcal{K}} \mathbb{E}_\theta\left\{\nabla_\theta\left[\log \tilde{p}_\mathcal{K}\left(y^K_{[T]}|\theta\right)\right]\nabla_\theta\left[\log \tilde{p}_\mathcal{K}\left(y^K_{[T]}|\theta\right)\right]^\top\right\},$$

where again we used our simulation feedback being approximatively 1.

Combining the definitions of mean and variance with these approximate Bartlett identities leads to our proposed estimates of the sensitivity and variability matrix.

Appendix C Experiments

Here, we provide additional details on the experiments reported in the main paper, as well as some extra experiments that were excluded to keep the paper concise. For experiments from the main paper, the model, model parameters, SimBa-CL versions, and P remain the same as described in the main text.

C.1 Empirical evaluation of the KL-divergence

In this section, we expand more on the empirical KL-divergence, which is used as an evaluation metric to measure the similarities between different SimBa-CL.

It is essential to recognise that any distribution $q(x)$ can be approximated empirically as $q(x) \approx \sum_{z\in[E]} q(z)\delta_z(x)$, where E corresponds to the number of evaluations of x. While this approximation provides a sparse representation of $p(x)$, it offers nonetheless a means to estimate the KL-divergence as follows:

$$\mathbf{KL}\left[p(\mathbf{x})\,||q(\mathbf{x})\right] \approx \sum_{e\in[E]} p(x^e)\log\left(\frac{p(x^e)}{q(x^e)}\right).$$

We can then simulate multiple times from the model to have some approximate evaluation of the chosen SimBa-CL, and we can then compare different SimBa-CL using the above evaluation metric.

Note that after simulating multiple times from the model we have a sample per each SimBa-CL and we can create multiple samples by leaving one simulation out at a time. This will allow us to produce a sample of KL-divergences from which we can compute mean and variance. The table with mean and variance for our experiments is reported in the main paper. However, as we are comparing distributions another valid comparison is to graphically look at the boxplots of the log SimBa-CL, which we report here.

Figure C1 depicts a comparison between the logarithm of the fully factorized SimBa-CL with and without feedback. The distribution of the two log-likelihoods appears similar, indicating that excluding the feedback does not result in a significant loss of precision. Figure C2 compares the logarithm of the fully factorized SimBa-CL without feedback with the logarithm of the coupled SimBa-CL without feedback. Again we can observe that the two distributions become more and more similar when we increase N and reduce the variance in the system.

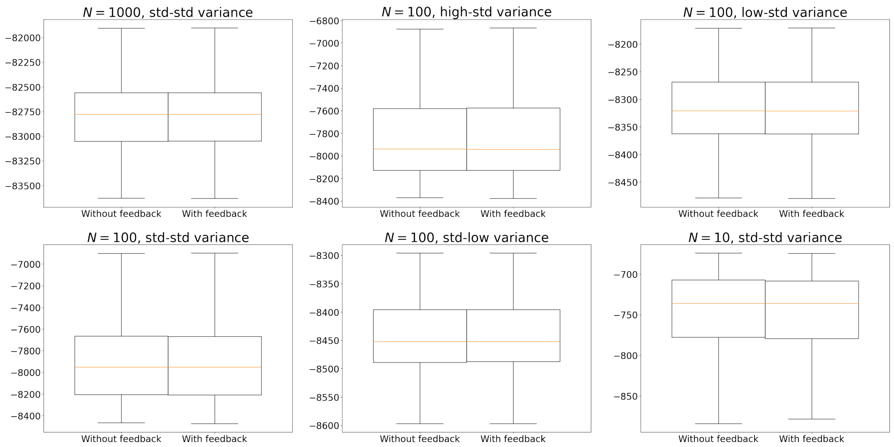

Fig. C1: Comparing empirical KL between fully factorized SimBa-CL with feedback and fully factorized SimBa-CL without feedback under different scenarios.

C.2 Optimization of the parameters

To optimize the parameters of our models and compute the maximum SimBa-CL estimator we generally used Adam optimizer (Kingma and Ba, 2014). Precisely, we provide gradient via automatic differentiation in TensorFlow and use the built-in optimizer. As a loss function we used $-\sum_{n\in[N]}\log(\tilde{p}(y^n_{[T]}|\theta))/(NT)$ with initial learning rate for Adam given by 0.1. We run 500 optimization steps for the 9-dimensional synthetic data scenario and 3000 for the FM data. We check convergence via the stability of the loss function. We also evaluate SimBa-CL using 500 simulations, but just 100 for

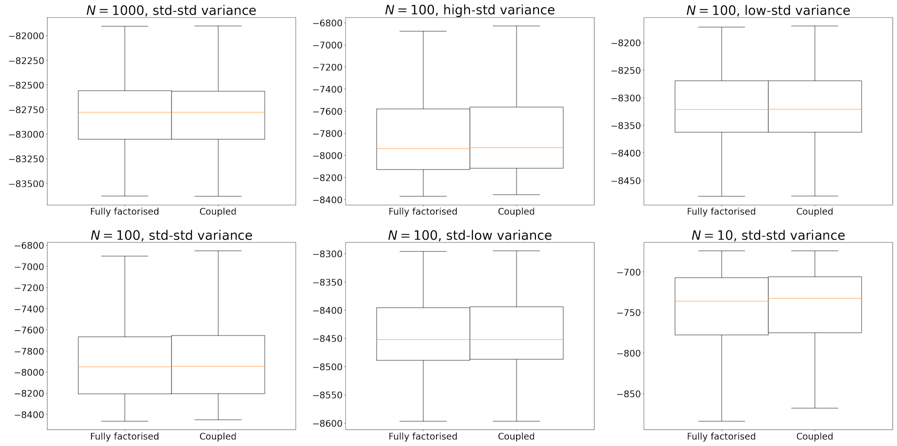

Fig. C2: Comparing empirical KL between fully factorized SimBa-CL without feedback and coupled SimBa-CL without feedback under different scenarios.

the FM data to meet the memory constraints. For the 2-dimensional synthetic data scenario, we use vanilla gradient descent with learning rate 100 and 200 optimization steps, again we check convergence via the stability of the loss function.

Convergence plots are reported below for the different scenarios. We can always notice that the optimization process can find a local minimum of the loss function and that we stop our optimization after convergence is reached.

It is important to comment on the optimization for the FM experiment. In the figure we can spot multiple drops while performing the optimization, these drops refer to the failure of the optimization procedure where the gradient has pushed the parameters in regions with zero likelihood. To solve this issue we reset the optimization by sampling a new random initial condition and restart the optimization from there, resulting in the vertical drops in Figure C3. Even though this might lead to some optimizations not converging, remark that we run 100 optimization procedures in parallel and choose the best one in terms of SimBa-CL score.

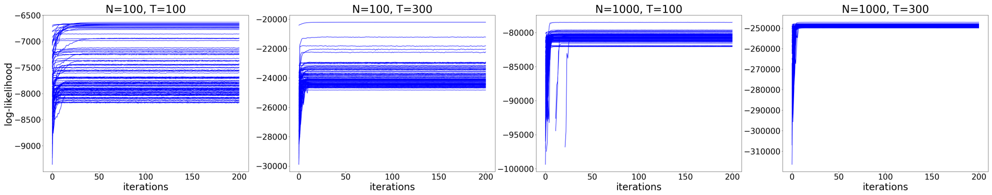

Fig. C3: Log-likelihood convergence during the optimization of β_λ, when studying the asymptotic properties of SimBa-CL.

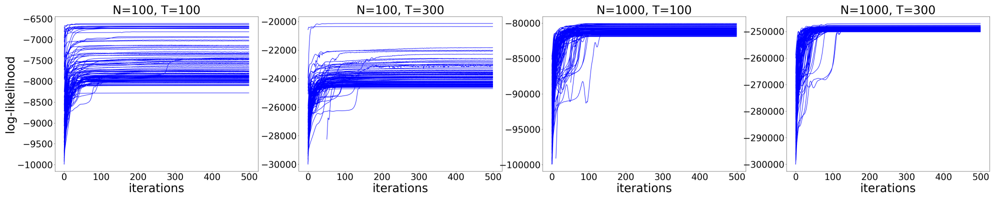

Fig. C4: Log-likelihood convergence during the optimization of all the nine parameters, when studying the asymptotic properties of SimBa-CL.

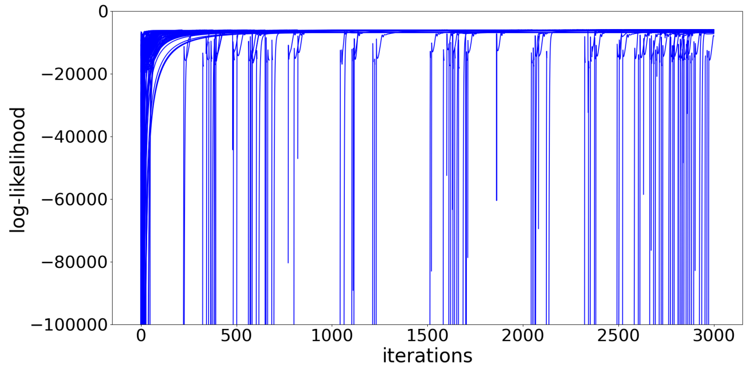

Fig. C5: Log-likelihood convergence during the optimization for the FM experiment.

C.3 Asymptotic properties of SimBa-CL

In this section, we provide some additional details on the asymptotic properties of SimBa-CL. Specifically, we provide a graphical representation of the empirical coverage for both the 2-dimensional and 9-dimensional case. For the 2 dimensional case, we also provide a graphical representation of the confidence sets, which are ellipsoids.

It is obvious from Figure C7 that the noise is dominating our computations, meaning that getting good estimates of the expectation and variance in $S(\theta)$ and $V(\theta)$ is not easy, as they refer to the full space $\mathcal{Y}^{TN}$. However, using the Bartlett identities lead to less noisy estimates as now $S(\theta)$ and $V(\theta)$ are expressed as sums of expectation on the space $\mathcal{Y}^T$. This computational trick along with SimBa-CL without feedback being close in the large population limit to SimBa-CL with feedback, where the Bartlett identities hold exactly, can explain the better coverage experienced in Figure C8. Further theoretical studies are needed to provide a more formal justification.

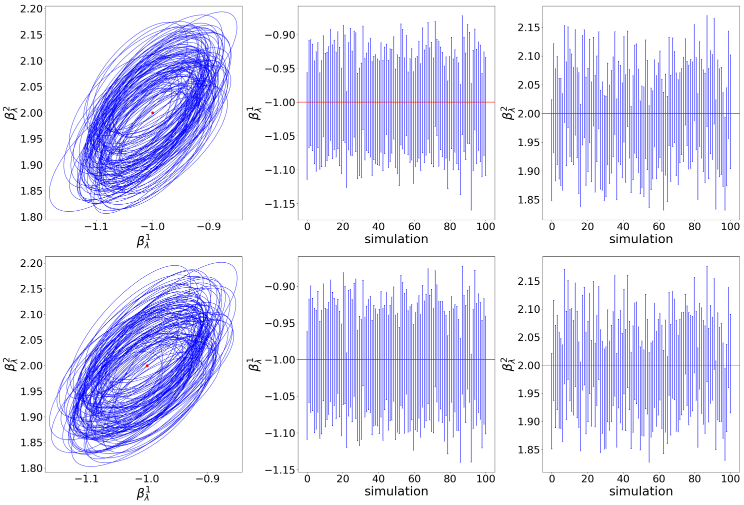

Fig. C6: Coverage of the confidence sets with and without Bartlett identities. The first row is for the latter and the second row is for the former. From left to right, graphical coverage of β_λ, β_λ^1 (marginally) and β_λ^2 (marginally). Dots or solid lines are used for the true parameters.

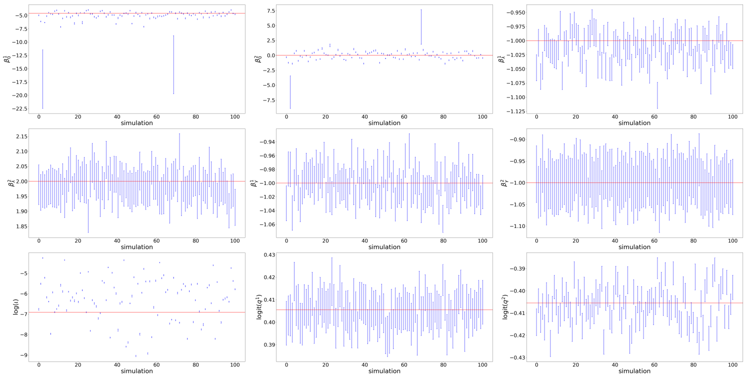

Fig. C7: Coverage of the confidence intervals from the diagonal of the Godambe information matrix estimated without the approximate Bartlett identities. Parameter labels are displayed on the x-axis. Solid lines are used for the true parameters.

C.4 Spatial SIS

Consider an individual-based susceptible-infected-susceptible (SIS) model as the one from previous experiments. Suppose however that the spatial interaction is not homogeneous, and that a spatial kernel is measuring the infection pressure from one individual to the other. We then have an initial probability of infection $\left(1/1+\exp\left(-\beta_0^\top w_n\right)\right)$ and a transition kernel with a probability of transitioning from

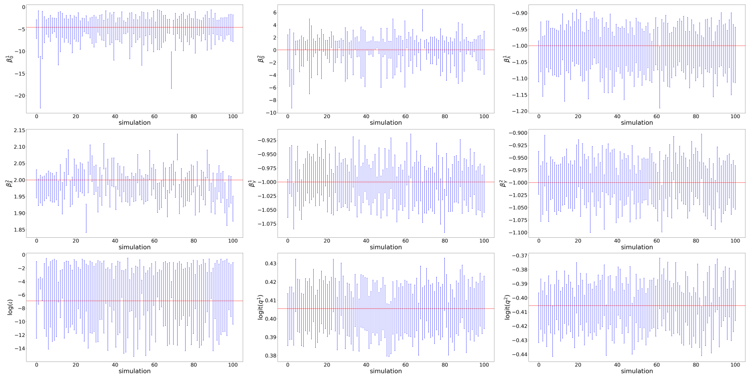

Fig. C8: Coverage of the confidence intervals from the diagonal of the Godambe information matrix estimated with the approximate Bartlett identities. Parameter labels are displayed on the x-axis. Solid lines are used for the true parameters.

S to I of:

$$1 - e^{-\lambda_n \left(\frac{\sum_{\bar{n} \in [N]} s(n,\bar{n},\psi) \mathbb{I}\left(x_{t-1}^{\bar{n}}=2\right)}{N} + \iota \right)},$$

and from I to S of $1 - e^{-\gamma_n}$, where $\lambda_n = \left(1/1 + \exp\left(-\beta_\lambda^\top w_n\right)\right)$ and $\gamma_n = \left(1/1 + \exp\left(-\beta_\gamma^\top w_n\right)\right)$ and $s(n, \bar{n}, \psi) := \exp\left(-E_{n,\bar{n}}^2/(2\psi^2)\right)$ with $E_{n,\bar{n}}$ euclidean distance between n and $\bar{n}$ and ψ positive parameters. We then consider the same emission distribution of our baseline SIS. For this model we set our baseline to $N = 1000$, $T = 100$, w_n to be such that $w_n^1 = 1$ and $w_n^2 \sim \textbf{Normal}\,(0, 1)$, and the data generating parameters $\beta_0 = [0.1, 0]^\top$, $\beta_\lambda = [-1, 2]^\top$, $\beta_\gamma = [-1, -1]^\top$, $q = [0.6, 0.4]^\top$, $\iota = 0.01$ and $\psi = 1$.

Given the above model, we can repeat a similar experiment to the one from the main paper and learn the parameters on a grid to study the shape of the SimBa-CL surfaces. In this study, we consider only fully factorized SimBa-CL with and without feedback and we have excluded the general partition case. As for the main paper we set $P = 1024$.

The comments on $\beta_0, \beta_\lambda, \beta_\gamma, q$ are the same as for the homogeneous scenario. ι and ψ needs some additional attention. Firstly notice that the more we increase ψ the more the spatial effect is strong and we highly penalise infected that are far away. This automatically tells us that over a certain threshold, it will be useless to increase ψ as the penalization is already very strong. Secondly, there is an obvious identifiability issue when looking at ι and ψ together. Indeed, increasing ψ and decreasing ι will lead to similar epidemics, where the difference is that most of the infections are either coming from the spatial interaction or the environment. This "banana" shape makes these two parameters hard to learn and we have to be careful when reporting uncertainty around them to avoid being overconfident on a local maxima.

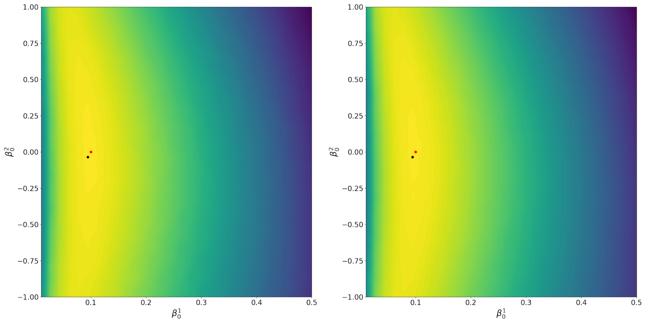

Fig. C9: Profile likelihood for β_0 in spatial SIS.

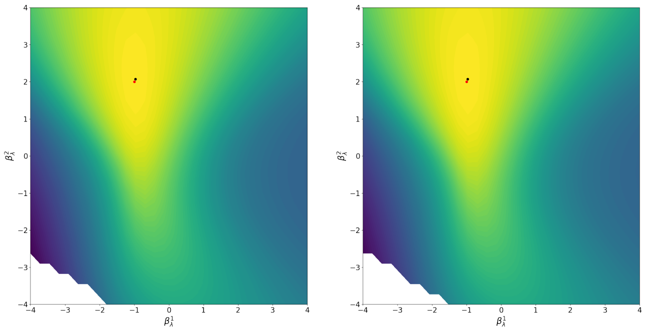

Fig. C10: Profile likelihood for β_λ in spatial SIS.

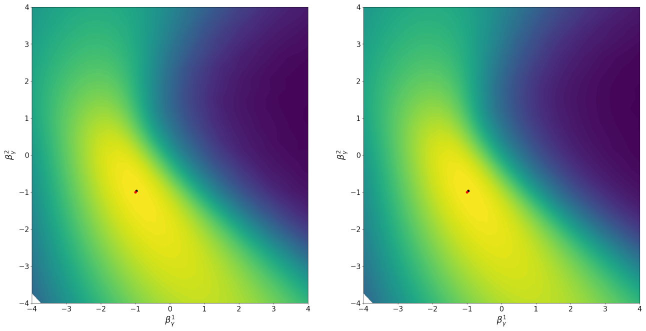

Fig. C11: Profile likelihood for β_γ in spatial SIS.

C.5 FM modelling choices

When working with the foot and mouth data we can have different modelling choices. One option is to learn the initial probability of infection as a common parameter across

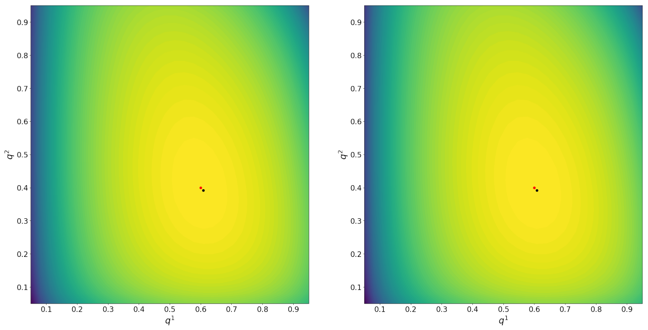

Fig. C12: Profile likelihood for q in spatial SIS.

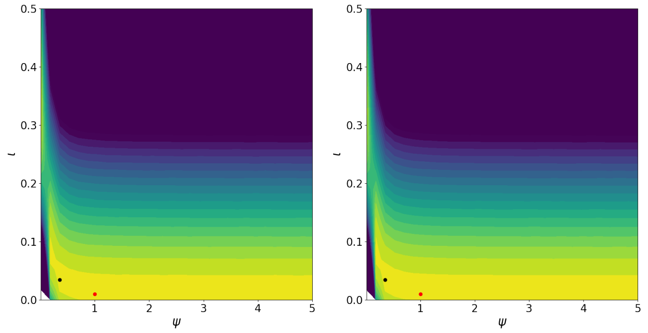

Fig. C13: Profile likelihood for ι and ψ in spatial SIS.

infected farms:

$$p(x_0^n|\theta) = \begin{bmatrix} 1 - p_0\mathbb{I}(n \in F_I) \\ p_0\mathbb{I}(n \in F_I) \\ 0 \\ 0 \end{bmatrix},$$

where F_I is the set of infected farms over time and so n belongs to F_I if it will be observed infected at some time t. This leads to satisfying likelihood scores, but to unreal epidemics, as it sets $p_0 \approx 1$ meaning that all the epidemics observed in the future are already infected at time 0 and no spatial interaction is learned.

Another option is to reverse engineer the infection process and use a geometric distribution for the infection time:

$$p(x_0^n|\theta) = \begin{bmatrix} 1 - \left(1 - e^{-\gamma}\right) e^{-\gamma\tau_n} \\ \left(1 - e^{-\gamma}\right) e^{-\gamma\tau_n} \\ 0 \\ 0 \end{bmatrix},$$

where τ_n is the infection time of individual n. This again leads to satisfying likelihood scores, but it is susceptible to criticism given that we are informing the initial distribution with future observation.

More discussion can follow regarding the choices of the spatial kernel and covariates. We decided to not normalize the covariates and follow the approach of Jewell et al. (2009), even though normalization of the covariates might lead to better results as explained in Jewell et al. (2013). In terms of the spatial kernel, an obvious choice is the use of a Gaussian spatial kernel, where the decay is modeled through a Gaussian function.

Even though optimal modeling choices for the FM data is an interesting problem, this is beyond the scope of this experiment, which is included as a pure real data application.

C.6 Limitations and extensions

In this section, we provide some experiments on the limitations and extensions of SimBa-CL.

C.6.1 Conditional SimBa-CL

As unconditional simulations from the model could lead to inconsistencies with the data, we try to test a conditional version of SimBa-CL without feedback. Specifically, at each time step, we simulate from $\tilde{p}\left(x_t^n | y_{[t]}^n, x_{[0:t-1]}^{\setminus n}\right)$ instead from the model and use these samples for the Monte Carlo estimate.

We compare the unconditional and the conditional SimBa-CL in terms of log-likelihood mean and variance, and average filtering performance. The former is self-explanatory, the latter is considering the ground truth for the latent process and computing the average prediction probability of the correct state over time and population. We consider our baseline individual-based SIS model with a varying population size $N = 100, 1000, 2000$, a varying environmental effect $\iota = 0.00001, 0.001, 0.1$ corresponding to the low, medium, high scenarios, and a varying $q = [0.2, 0.1], [0.6, 0.4], [0.9, 0.9]$ corresponding to the low, medium, high scenarios. We also set $P = 500$. Table C2 shows that the conditional SimBa-CL is generally associated with a lower variance in both the log-likelihood and the filtering performance. This effect is less evident for large population sizes and large environmental effects.

Simba-CL	N	ϵ	q	log-likelihood	filtering performance
Unconditional	100	low	low	-5131.96(2.6794)	0.84(0.0005827)
Conditional	100	low	low	-5100.07(0.0432)	0.83(6.01e-05)
Unconditional	100	low	medium	-6785.45(3.4968)	0.97(0.0018434)
Conditional	100	low	medium	-6738.07(0.0425)	1.0(6.9e-06)
Unconditional	100	low	high	-3340.12(3.4506)	1.0(0.00021)
Conditional	100	low	high	-3295.73(0.0156)	1.0(3e-07)
Unconditional	100	medium	low	-5179.42(1.8061)	0.85(0.0010506)
Conditional	100	medium	low	-5143.67(0.2772)	0.87(0.0002244)
Unconditional	100	medium	medium	-8313.9(2.3432)	0.91(0.0001687)
Conditional	100	medium	medium	-8276.77(0.0822)	0.91(6.2e-06)
Unconditional	100	medium	high	-5117.49(4.3901)	0.99(8.89e-05)
Conditional	100	medium	high	-5013.39(0.0461)	0.99(5e-07)
Unconditional	100	high	low	-4913.78(0.0408)	0.76(2.04e-05)
Conditional	100	high	low	-4913.82(0.0245)	0.76(9e-06)
Unconditional	100	high	medium	-8461.96(0.1054)	0.89(2.59e-05)
Conditional	100	high	medium	-8462.15(0.0443)	0.89(5.8e-06)
Unconditional	100	high	high	-6001.89(0.1919)	0.98(4.7e-06)
Conditional	100	high	high	-5997.51(0.024)	0.98(5e-07)
Unconditional	1000	low	low	-50951.32(1.1928)	0.82(0.0001415)
Conditional	1000	low	low	-50947.66(0.0539)	0.82(2.23e-05)
Unconditional	1000	low	medium	-82876.23(0.6251)	0.91(7.08e-05)
Conditional	1000	low	medium	-82876.2(0.0689)	0.91(2.9e-06)
Unconditional	1000	low	high	-53233.93(1.8217)	0.99(1.39e-05)
Conditional	1000	low	high	-53204.11(0.0351)	0.99(2e-07)
Unconditional	1000	medium	low	-50693.04(1.0425)	0.8(7.96e-05)
Conditional	1000	medium	low	-50681.28(0.064)	0.8(1.34e-05)
Unconditional	1000	medium	medium	-81994.59(0.4083)	0.91(4.73e-05)
Conditional	1000	medium	medium	-81992.01(0.0737)	0.91(3e-06)
Unconditional	1000	medium	high	-55111.02(1.5721)	0.99(9.2e-06)
Conditional	1000	medium	high	-55098.72(0.0323)	0.99(2e-07)
Unconditional	1000	high	low	-50277.48(0.0492)	0.77(7.6e-06)
Conditional	1000	high	low	-50277.68(0.0271)	0.77(3.1e-06)
Unconditional	1000	high	medium	-84675.37(0.0926)	0.89(4.8e-06)
Conditional	1000	high	medium	-84674.12(0.0404)	0.89(1.2e-06)
Unconditional	1000	high	high	-61529.04(0.3132)	0.98(1.5e-06)
Conditional	1000	high	high	-61511.64(0.0268)	0.98(1e-07)
Unconditional	2000	low	low	-101230.21(1.987)	0.81(0.0001159)
Conditional	2000	low	low	-101203.51(0.1243)	0.81(2e-05)
Unconditional	2000	low	medium	-163284.12(2.081)	0.91(3.94e-05)
Conditional	2000	low	medium	-163266.38(0.0707)	0.91(2.1e-06)
Unconditional	2000	low	high	-110632.57(3.8001)	0.99(5.5e-06)
Conditional	2000	low	high	-110567.61(0.0306)	0.99(1e-07)
Unconditional	2000	medium	low	-101850.23(0.3624)	0.81(7.66e-05)
Conditional	2000	medium	low	-101849.97(0.057)	0.82(1.44e-05)
Unconditional	2000	medium	medium	-163018.38(0.1773)	0.91(3.05e-05)
Conditional	2000	medium	medium	-163021.54(0.0658)	0.91(2e-06)
Unconditional	2000	medium	high	-107468.3(7.3799)	0.99(7.3e-06)
Conditional	2000	medium	high	-107230.03(0.0386)	0.99(1e-07)
Unconditional	2000	high	low	-99940.46(0.0389)	0.76(5e-06)
Conditional	2000	high	low	-99940.57(0.0199)	0.76(2.2e-06)
Unconditional	2000	high	medium	-172386.18(0.1524)	0.89(4.1e-06)
Conditional	2000	high	medium	-172383.19(0.0434)	0.89(1e-06)
Unconditional	2000	high	high	-121700.89(0.124)	0.98(1e-06)
Conditional	2000	high	high	-121698.41(0.02)	0.98(1e-07)

Table C2: Log-likelihood and filtering performance for unconditional and conditional SimBa-CL across different scenarios.

C.6.2 Gradient considerations

We are differentiate quantities of the form $\sum_{n\in[N]} \log\left(p(y^n_{[T]}|\theta)\right)$, with $\tilde{p}$ for SimBa-CL without feedback. This reduces to differentiating $p(y^n_{[T]}|\theta)$:

$$\frac{\partial p(y^n_{[T]}|\theta)}{\partial\theta} = \sum_{x^{\setminus n}_{[0:T-1]}} p\left(x^{\setminus n}_{[0:T-1]}|\theta\right) \frac{\partial p\left(y^n_{[T]}|x^{\setminus n}_{[0:T-1]},\theta\right)}{\partial\theta} + \sum_{x^{\setminus n}_{[0:T-1]}} p\left(y^n_{[T]}|x^{\setminus n}_{[0:T-1]},\theta\right) \frac{\partial p\left(x^{\setminus n}_{[0:T-1]}|\theta\right)}{\partial\theta}.$$

In our experiments, we consider TensorFlow's default automatic differentiation, which consists of the approximation:

$$\frac{\partial p(y^n_{[T]}|\theta)}{\partial\theta} \approx \frac{1}{P}\sum_{i\in[P]} \frac{\partial p\left(y^n_{[T]}|x^{(i),\setminus n}_{[0:T-1]},\theta\right)}{\partial\theta}.$$

As discussed in the main paper, we could also use the Gumbel-Softmax trick (Gumbel, 1954; Maddison et al., 2016; Jang et al., 2016), which approximates the one-hot encoding representation of categorical random variable with a probability vector that converges to the one-hot encoding as the temperature parameter goes to zero. Indeed, suppose we have an M-dimensional categorical distribution $X \sim Cat(\pi)$, with X one-hot encoding representation. We can simulate it using the Gumbel distribution as follows:

$$X = \text{one-hot}\left(\arg\max_{k=1,\dots,M}\{\log(\pi^{(k)}) + G_k\}_k\right), \qquad \text{with } G_k \sim Gumb(0,1) \text{ i.i.d.},$$

with one-hot(k) being the vector of all zeros with 1 in the kth position. Now, the arg max can be substituted with a Softmax to produce a continuous function:

$$X^{(k)} \approx \frac{\exp\left(\frac{\log(\pi^{(k)})+G_k}{\tau}\right)}{\sum_j \exp\left(\frac{\log(\pi^{(j)})+G_j}{\tau}\right)} \quad \text{for } j = 1,\dots,M,$$

which converges to the arg max, i.e. a one-hot encoding vector, when $\tau \to 0$. We are now considering a continuous function of i.i.d. random variables that are independent of the parameters of the categorical distribution, meaning that we can apply the reparameterization trick (Blundell et al., 2015), and exchange expectations and derivatives.

	N	Without τ	$\tau = 0.1$	$\tau = 0.01$	$\tau = 0.0001$
At DGP (0)	100	-0.0023(8e-05)	-0.0042(0.00436)	-0.0038(0.00941)	-0.0026(0.00044)
Not at DGP (0)	100	0.0093(0.00021)	0.0183(0.00278)	0.0163(0.00938)	0.012(0.00324)
At DGP (0)	1000	-0.0006(8e-05)	-0.0011(0.00116)	-0.0019(0.00794)	-0.0009(0.00113)
Not at DGP (0)	1000	0.0289(0.00027)	0.057(0.0051)	0.0521(0.01858)	0.0394(0.01097)
At DGP (1)	100	0.0005(2e-05)	1e-04(0.00179)	0.0013(0.00679)	0.0005(0.00033)
Not at DGP (1)	100	-0.0011(0.00017)	-0.0024(0.00125)	-0.0028(0.00369)	-0.0022(0.00426)
At DGP (1)	1000	-0.0002(2e-05)	-0.0003(0.00054)	-0.0005(0.00223)	-0.0003(0.00055)
Not at DGP (1)	1000	-0.0188(9e-05)	-0.0187(0.00179)	-0.0197(0.01062)	-0.0204(0.00806)

Table C3: Gradients estimates for β_λ in different scenarios. (0) refers to the first component. (1) refers to the second component.

Applying this to our scenario we get something along the lines of:

$$\begin{aligned}
\frac{\partial p(y^n_{[T]}|\theta)}{\partial\theta} &= \frac{\partial}{\partial\theta}\mathbb{E}_{x_{[0:T-1]}}\left[p\left(y^n_{[T]}|x^{\backslash n}_{[0:T-1]},\theta\right)\right] \\
&\approx \frac{\partial}{\partial\theta}\mathbb{E}_{G_{[N],[0:T-1]}}\left[p\left(y^n_{[T]}|s(G_{[N]\backslash n,[0:T-1]},\theta,\tau),\theta\right)\right] \\
&= \mathbb{E}_{G_{[N],[0:T-1]}}\left[\frac{\partial}{\partial\theta}p\left(y^n_{[T]}|s(G_{[N]\backslash n,[0:T-1]},\theta,\tau),\theta\right)\right] \\
&= \mathbb{E}_{G_{[N],[0:T-1]}}\Bigg[\left\{\frac{\partial s(G_{[N]\backslash n,[0:T-1]},\theta,\tau)}{\partial\theta}\frac{\partial p\left(y^n_{[T]}|s,\theta\right)}{\partial s}\right\}_{s=s(G_{[N]\backslash n,[0:T-1]},\theta,\tau)} \\
&\qquad\qquad + \left\{\frac{\partial p\left(y^n_{[T]}|s,\theta\right)}{\partial\theta}\right\}_{s=s(G_{[N]\backslash n,[0:T-1]},\theta,\tau)}\Bigg],
\end{aligned}$$

where $G_{[N],[0:T-1]} := (G_{n,t})_{n\in[N],t\in[0:T-1]}$ are i.i.d. Gumbel random variables, and s is a function of the Gumbel random variables, the parameter θ, and the temperature parameter τ, which is a composition of continuous functions. The Gumbel-Softmax relaxation and the reparameterization trick are implemented in tensorflow_probability via the distribution *RelaxedOneHotCategorical*.

In this section, we compare the default *OneHotCategorical*, which we used in our experiments, with the *RelaxedOneHotCategorical*, in terms of gradients estimate and computational cost for our baseline SIS model.

Starting from gradient estimates, we consider our baseline SIS model for a population size $N = 100, 1000$ and we compute the gradient of β_λ both at the data-generating parameter DGP and in $\beta_\lambda = [0,0]^\top$. To compute the gradient we consider both *OneHotCategorical* which we refer to as the "Without τ" scenario and *RelaxedOneHotCategorical* for a $\tau = 0.1, 0.01, 0.0001$. We also set $P = 500$. Table C3 shows that all gradient estimates have the same sign in all scenarios, with these estimates being particularly close for large populations. It is also important to observe that TensorFlow's default implementation underestimates the variance of the gradient.

	N	T=10, P=300	P=500	T=50, P=100	P=300	P=500	T=100, P=100
Without τ	10	0.16(0.07)	0.16(0.06)	0.15(0.07)	0.28(0.06)	0.28(0.06)	0.3(0.07)
With τ		0.23(0.08)	0.23(0.22)	0.22(0.08)	0.38(0.08)	0.38(0.08)	0.39(0.08)
Without τ	100	0.16(0.07)	0.15(0.07)	0.15(0.07)	0.3(0.07)	0.3(0.07)	0.29(0.07)
With τ		0.21(0.08)	0.21(0.08)	0.21(0.08)	0.39(0.08)	0.39(0.08)	0.39(0.08)
Without τ	500	0.16(0.14)	0.17(0.07)	0.2(0.07)	0.31(0.07)	0.33(0.07)	0.39(0.07)
With τ		0.2(0.08)	0.24(0.08)	0.3(0.08)	0.39(0.08)	0.46(0.08)	0.58(0.08)
Without τ	1000	0.16(0.07)	0.22(0.07)	0.32(0.07)	0.29(0.07)	0.42(0.07)	0.62(0.08)
With τ		0.21(0.08)	0.33(0.09)	0.48(0.09)	0.41(0.08)	0.65(0.09)	OOM
Without τ	1500	0.18(0.08)	0.3(0.09)	0.46(0.09)	OOM	OOM	OOM
With τ		0.25(0.08)	0.45(0.09)	0.7(0.1)	OOM	OOM	OOM
Without τ	2000	0.19(0.07)	0.39(0.2)	0.59(0.09)	OOM	OOM	OOM
With τ		0.27(0.08)	0.57(0.08)	OOM	OOM	OOM	OOM
Without τ	2500	0.2(0.07)	0.45(0.08)	OOM	OOM	OOM	OOM
With τ		OOM	OOM	OOM	OOM	OOM	OOM

Table C4: Time in seconds in different scenarios. "OOM" stands for out-of-memory. Columns that are grouped together have the same T.

Moving to the computational considerations, we again consider our baseline SIS model for a population size $N = 10, 100, 500, 1000, 1500, 2000, 2500$, a time horizon $T = 10, 50, 100$, and a number of particles $P = 100, 300, 500$. In Table C4 we report the time in seconds of running a gradient computation of TensorFlow's default implementation, "Without τ", and the continuous relaxation, "With τ". To fit within the margin we have reported only a few columns of all the simulations. The standard deviation of the computational time is reported in brackets and computed across 100 replicates. Overall we observe that the "Without τ" is 1.5 times faster than the "With τ" approach. Moreover, it is more frequent for the "With τ" approach to go out-of-memory (OOM). For instance, we were not able to run a single gradient computation with $N = 2500$. This is due to the heavier computational graph required by the "With τ" approach.

C.7 The effect of P

In this section, we graphically explore the effect of increasing P. We consider our baseline SIS model with $P = 10, 100, 1000$ and simulate data from it. Next, we define a grid for the parameter β_λ and compute SimBa-CL without feedback for each element of the grid. Specifically, we set β_λ to each grid value while keeping the true values for the other parameters. The resulting log-likelihood surfaces are shown in Figure C14.

We observe that the primary effect of P is on the noisiness of the log-likelihood surface. For small values of P (left-hand side of the figure), the log-likelihood surface is less smooth compared to that for larger values of P (right-hand side of the figure). This effect is particularly noticeable in regions with low log-likelihood values. However, the recovery of the parameter does not appear to be significantly affected.

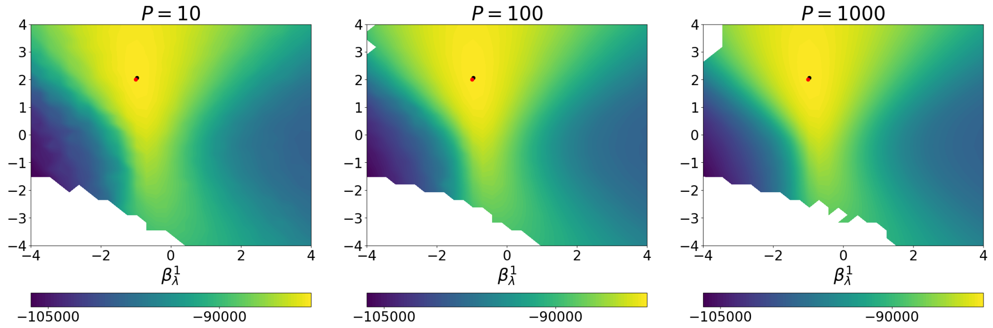

Fig. C14: Profile log-likelihood surfaces for β_λ obtained from fully factorized SimBa-CL without feedback for different values of P. The red dot indicates the data-generating parameter, and the black dot denotes the maximum on the grid.

References

Blundell, C., Cornebise, J., Kavukcuoglu, K., Wierstra, D.: Weight uncertainty in neural network. In: Bach, F., Blei, D. (eds.) Proceedings of the 32nd International Conference on Machine Learning. Proceedings of Machine Learning Research, vol. 37, pp. 1613–1622. PMLR, Lille, France (2015). https://proceedings.mlr.press/v37/blundell15.html

Gumbel, E.J.: Statistical Theory of Extreme Values and Some Practical Applications: a Series of Lectures vol. 33. US Government Printing Office, New York (1954)

Jewell, C., Brown, J., Keeling, M., Green, L., Roberts, G., *et al.*: Bayesian epidemic risk prediction-knowledge transfer and usability at all levels. In: Society for Veterinary Epidemiology and Preventive Medicine. Proceedings of a Meeting Held in Madrid, Spain, 20-22 March 2013, pp. 127–142 (2013). Society for Veterinary Epidemiology and Preventive Medicine

Jang, E., Gu, S., Poole, B.: Categorical reparameterization with gumbel-softmax. arXiv preprint arXiv:1611.01144 (2016)

Jewell, C.P., Kypraios, T., Neal, P., Roberts, G.O.: Bayesian analysis for emerging infectious diseases. Bayesian Analysis **4**, 465–496 (2009)

Kingma, D.P., Ba, J.: Adam: A method for stochastic optimization. arXiv preprint arXiv:1412.6980 (2014)

Maddison, C.J., Mnih, A., Teh, Y.W.: The concrete distribution: A continuous relaxation of discrete random variables. arXiv preprint arXiv:1611.00712 (2016)
